# Evaluation of the Psoriasis Transcriptome across Different Studies by Gene Set Enrichment Analysis (GSEA)

**DOI:** 10.1371/journal.pone.0010247

**Published:** 2010-04-20

**Authors:** Mayte Suárez-Fariñas, Michelle A. Lowes, Lisa C. Zaba, James G. Krueger

**Affiliations:** 1 Laboratory for Investigative Dermatology, The Rockefeller University, New York, New York, United States of America; 2 Center for Clinical and Translational Science, The Rockefeller University, New York, New York, United States of America; The University of Queensland, Australia

## Abstract

**Background:**

Our objective was to develop a consistent molecular definition of psoriasis. There have been several published microarray studies of psoriasis, and we compared disease-related genes identified across these different studies of psoriasis with our own in order to establish a consensus.

**Methodology/Principal Findings:**

We present a psoriasis transcriptome from a group of 15 patients enrolled in a clinical study, and assessed its biological validity using a set of important pathways known to be involved in psoriasis. We also identified a key set of cytokines that are now strongly implicated in driving disease-related pathology, but which are not detected well on gene array platforms and require more sensitive methods to measure mRNA levels in skin tissues. Comparison of our transcriptome with three other published lists of psoriasis genes showed apparent inconsistencies based on the number of overlapping genes. We extended the well-established approach of Gene Set Enrichment Analysis (GSEA) to compare a new study with these other published list of differentially expressed genes (DEG) in a more comprehensive manner. We applied our method to these three published psoriasis transcriptomes and found them to be in good agreement with our study.

**Conclusions/Significance:**

Due to wide variability in clinical protocols, platform and sample handling, and subtle disease-related signals, intersection of published DEG lists was unable to establish consensus between studies. In order to leverage the power of multiple transcriptomes reported by several laboratories using different patients and protocols, more sophisticated methods like the extension of GSEA presented here, should be used in order to overcome the shortcomings of overlapping individual DEG approach.

## Introduction

The study of human diseases such as psoriasis has benefited significantly from analysis of the transcriptome, the global gene expression of a diseased tissue compared to its healthy counterpart. However, as more studies are carried out independently in multiple laboratories, effective methodology to leverage these multiple studies becomes necessary. Such methodologies have significant hurdles to overcome: first, multiple studies are likely to use different platforms, different sample dissection, handling and preparation, and, especially, different definition of the non-diseased counterpart, resulting in different physical samples being hybridized against different platforms [1], [2], [3]. Second, computational analysis and statistical treatment required to assess the transcriptome are just as likely to be considerably different.

In many instances, all that is available from published studies are lists of differentially expressed genes (DEG). It is tempting to evaluate the agreement between studies simply by evaluating the intersection between the published lists, the “Venn diagram approach”. However, such an approach suffers serious methodological shortcomings [4], [5], [6]. Use of the original raw data of the studies has shown that studies which are apparently discordant in terms of their overlapping individual DEG lists are, in fact, both concordant and predictive [4], [5]. However, most of the time the original raw data is unavailable, and furthermore a complete reanalysis of all data is needlessly laborious. In such cases use of the published lists of DEG is a necessity. Here we present an extension to the widely used Gene Set Enrichment Analysis (GSEA) method, where it suffices to have full access to the complete list of gene expression values for a single study, while the remaining studies only require the DEG list.

In the last few years, the use of Gene-Sets approach had emerged as a powerful tool to identify sets of functionally related genes or pathways that are associated with a disease phenotype [7], [8]. Gene-Sets based methods were designed to address limitations of conventional single gene methods [6] by evaluating differential expression patterns of gene groups instead of individual genes. GSEA, introduced by Mootha *et al* [9] and further developed by Subramanian *et al* [10], was one of the first method using the Gene-Sets approach, and is arguably the most widely used of such methods. Here we use GSEA as a conventional approach to identify pathways related to the psoriatic phenotype. Furthermore, we propose to extend the use of GSEA as a tool to easily cross-compare prior lists of DEG genes.

We developed this method specifically to compare several high-quality studies that defined the psoriasis transcriptome by identifying DEG between psoriatic lesions and non-lesional tissue from the same patients [11], [12], [13], [14], [15]. Those studies had identified key genes involved in psoriasis pathogenesis, using a non-biased approach. Because the genomic data for more recent studies is more comprehensive than in the earlier studies due to the larger number of genes represented in the latest Affymetrix chips, we chose to compare the transcriptomes for studies published since 2003 [12], [14], [15].

We recently conducted a clinical trial of 15 psoriasis patients with the TNF inhibitor etanercept [16], and performed a time-course experiment using HGU 133 2.0 microarray chips [17]. By analyzing the baseline data from this experiment, we generated our psoriasis transcriptome comparing baseline-paired values of lesional versus non-lesional skin. We have compared our transcriptome with the three others described above, and introduce the concept of using GSEA as a more robust way of comparing genomic data.

## Results

### Disease-modulated genes

The analysis of our data identified a psoriasis transcriptome composed of 732 up-regulated probesets (representing 579 genes with unique ENTREZ identifier) and 890 down-regulated probesets (703 genes) with fold change (FCH) greater than 2 and false discovery rate (FDR) less than 0.05 (Table 1, and Table S1). Certain genes with low expression on the Affymetrix chip were confirmed by RT-PCR, and will be discussed in the next section.

**Table 1:** Description of studies.

	Zhou	Yao	Gudhjonsson	Suárez-Fariñas
Platform/chips	hgu95 a,b,c,d,e	hgu133plus2	hgu133plus2	hgu133a2
Sample size	16	26	58	15
Expression Algorithm	dChip	GCRMA	RMA	GCRMA
Statistical test	t-test	Sam (paired) t-test	Paired t-test	Moderated paired t-test
Multiple Hypothesis correction	none	FDR through permutations	FDR through permutations	FDR Benjamini-Hochberg
Cut-off	FCH>2, p<0.05	FCH>2, q-value<0.05	FCH>2, FDR<0.05	FCH>2, FDR<0.05
# Up-regulated probesets (genes)	397 ps (270 genes)	1408 ps. (974 genes)	721 ps (508 genes)	732 ps (579 genes)
# of Down-regulated probesets (genes)	613 ps (397 genes)	1465 ps (853 genes)	364 ps (248 genes)	890 ps (703 genes)

To further consider the biological significance of our data, we used GSEA in the classical manner, to identify pathways that correlate with the psoriatic phenotype [10], [18]. GSEA evaluates how genes in queried pathways are distributed in the fold change (lesional versus non-lesional) ordered list generated by our data (all probesets included). This is quantified by using the Enrichment Score (ES), a weighted Kolmogorov-Smirnov-like statistic that evaluates if the members of the pathway are randomly distributed or found at the extremes (top or bottom) of the list. If genes in a pathway rank at the top of the new fold change list, *ie*. they are overrepresented at the top, then the enrichment score (ES) will be close to 1. Conversely if the ES = −1, then genes are overrepresented at the bottom of our fold change data. A perfect agreement is reached if ES = 1 for the up-regulated genes and ES = −1 for the down-regulated genes. A normalized enrichment score (NES) takes into account the number of genes in the pathway. A positive NES indicates that the list of genes is enriched at the “top” of the ordered fold change list, and a negative NES indicates that the list in question is enriched at the “bottom” of the list.

GSEA may be used with well known “canonical” pathways and Gene ontology categories, but also with sets that contain genes sharing the same transcription factor binding site, the same microRNA binding motif or the same cis-regulatory motif. It can also be used with curated collections such as GeneSigDb (http://compbio.dfci.harvard.edu/genesigdb), which contain gene signatures of cancer, viral and stem cell biology, and the CGP collection of the MSigDb (http://www.broadinstitute.org/gsea/msigdb/) that contains gene expression signatures of genetic and chemical perturbations, or computational derived sets such as cancer modules (presented in [19]).

We queried our psoriasis transcriptome with a set of cytokine-treated keratinocyte pathways for IL-17, TNF, IL-17+TNF, IFNγ, and IL-22, reported in [17], [20] and IL-1α [21]. GSEA showed that those pathways were enriched in lesional skin of psoriasis patients (Table 2). We used a list (“IL-17 Gaffen”) considered to be IL-17-gene targets defined by Shen *et al* [22]. This was also significantly enriched in our psoriasis transcriptome. An IFNα-keratinocyte pathway [14] was also significantly enriched in lesional skin, supporting the potential role for IFNα discussed by Yao *et al* [14]. A list of genes representing the transcriptome of inflammatory myeloid DCs [23] was also significantly enriched in lesional skin. In addition, the cell cycle and TLR signaling from the collection of canonical pathways (C2 CP) available at the Molecular Signature Database (MSigDb) were enriched in psoriasis lesional skin. Gudjonsson *et al* reported lack of evidence of enrichment of the Hedghog Signalling Pathways in psoriasis [24]. GSEA analysis did not detect any significant enrichment of this pathway with the psoriasis phenotype. (ES = 0.37, NES = 1.05 and p = 0.39).

**Table 2:** Pathways enriched in Psoriasis lesions by using GSEA.

PATHWAYS	No. of genes in pathway	ES	NES	FDR
IFNα Up in KC (Yao)	28	0.91	2.74	<10^−4^
IL17 and TNF Up in KC	30	0.89	2.69	<10^−4^
IL17 Up in KC	46	0.87	2.86	<10^−4^
IL1 Up in KC	34	0.85	2.66	<10^−4^
IL17 GAFFEN	27	0.81	2.43	<10^−4^
IFNγ Up in KC	872	0.47	2.31	<10^−4^
TNF Up in KC	472	0.46	2.14	<10^−4^
IL22 Up in KC	10	0.89	2.07	<10^−4^
Terminal Differentiation	33	0.69	2.10	<10^−4^
Inflammatory myeloid DCs (psoriasis)	121	0.42	1.68	<10^−4^
Cell Cycle (KEGG)	64	0.58	2.06	<10^−4^
TLR signaling Pathway (KEGG)	58	0.59	2.05	<10^−4^
Cytokine-Cytokine receptor interaction (KEGG)	111	0.44	1.71	0.02

### Comparison with other published studies

We next performed a comparative analysis of the DEG lists of these three studies [12], [14], [15] with our transcriptome. Table 1 summarizes the main characteristics of the four studies. Zhou's study which uses early hgu95 (a,b,c,d,e) chips, reported 397 up-regulated and 613 down-regulated probesets representing 270 and 397 unique genes respectively [15]. Yao *et al* conducted a study on hgu133plus2 chips and reported 1408 up-regulated and 1465 down-regulated probesets (974 and 853 genes respectively) [14]. More recently, Gudjonsson *et al* also using hgu133plus2 chips with a large sample size [12] reported a set of 721 up-regulated and 364 down-regulated probesets (508 genes and 248 genes).

**Figure 1:**
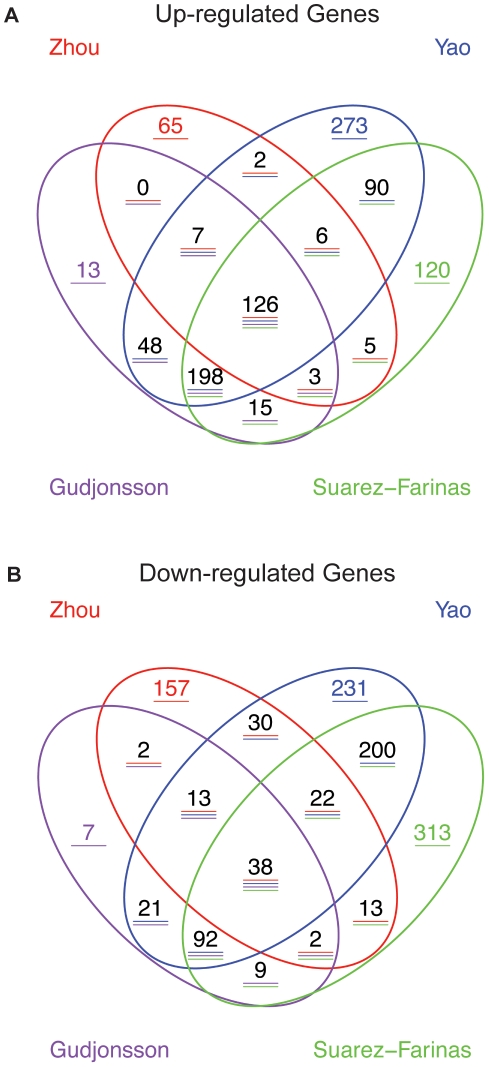
Comparison of four psoriasis transcriptomes. **A.** Venn diagram showing the comparison of the number of up-regulated genes in common and distinct for the four studies. **B.** Venn diagram showing the comparison of the number of down-regulated genes in common and distinct for the four studies.

Figure 1 shows a Venn diagram illustrating the intersection between the four studies. There were approximately 11,000 ENTREZ identifiers common to the 4 chip series, but only 126 genes were up-regulated (Figure 1A) and 38 down-regulated (Figure 1B) in all four studies. The numbers of upregulated genes in our transcriptome were similar to the Gudhjonsson group, but less than Yao's. The number of down-regulated genes were greater than the Gudhjonsson group, and again, less than Yao's.

The list of the DEG in common in the 4 studies (Table S2) contains many genes known to be upregulated in psoriasis, such as IFNγ-regulated genes STAT-1, STAT-3 and MX1; antimicrobial peptides (beta defensin 4, lipocalin 2, S100A7); and pro-inflammatory proteins such as IL-8, CXCL1, IL-1F9, TNFSF10/TRAIL. However, some genes known to be upregulated in psoriasis were only identified in one study. For example STAT2 was only identified in our study, JAK3 only in Yao's, and IL-12RB1 only in Zhou's study. The set of genes that were down-regulated in all 4 studies include CCL27, also called Cutaneous T cell –attracting chemokine (CTACK), which has a role in memory T cell homing to the skin [25], and Aquaporin 9, a member of a family of proteins that form water channels across membranes [26].

Some well-recognized inflammatory genes involved in psoriasis were not detected by most of the four studies, for example IFNγ, IL-17, iNOS. This is due to the fact that the expression of these genes is usually low on the Affymetrix gene array platform (0–4 range of expression in log_2_-scale) and hence fold change is not accurately measured. Most analysis pipelines filter out low abundance genes so they may be excluded from the statistical analysis, or the resultant fold change is very low, albeit significant. This is a major limitation of the use of these arrays for the study of these genes.

To confirm the role of these genes in psoriasis, we analyzed RT-PCR data of many of these inflammatory genes. We used data from the same clinical trial of etanercept treatment of psoriasis [16], however, we compared only baseline lesional skin and non-lesional skin. The fold change and p-value for each gene by RT-PCR is presented in Table 3, and all of these genes except LTA and p35 were significantly different in lesional skin (p<0.05). We also present the fold change of any of these genes that were detectable in any of the microarray studies. It can be seen that IL-23p19, IL-12/23p40, IL-22, IFNγ, IL-6 were not found to be differentially expressed by any of the four studies, but the fold change by RT-PCR was greater than 4. Furthermore, IL-17, IL-20, CCL4, iNOS and CCL3 were detected in only one study, and with a lower fold change than detected by RT-PCR (which was greater than 5.5). We also included the percentage of samples in our study with low intensity, as defined as expression values less than 4. For example, p19 has expression of less than 4. It can be seen that the genes that were not detected by any or only one microarray study had low abundance in more than 87% of the samples.

**Table 3:** RT-PCR validation.

		RT-PCR			Microarray FCH (log2)^1^				
Gene	Symbol	FCH (log2)	FCH	p.value	Suarez-Farinas	Zou	Yao	Gudjonsson	% Low Int^2^
p19	IL23A	2.66	6.34	0.011					100
p40	IL12B	4.03	16.32	1.8×10^−05^					97
LTA1	LTA	0.83	1.77	0.302					100
IL22	IL22	3.96	15.53	1.5×10^−4^					100
IFNγ	IFNg	2.28	4.85	2.8×10^−4^					100
IL4	IL4	−1.45	0.36	0.034					100
IL6	IL6	2.66	6.33	7.1×10^−4^					100
IL17	IL17A	6.17	71.87	3.8×10^−5^			1.15		93
IL20	IL20	3.95	15.48	1.4×10^−5^				1.03	
CCL4	CCL4	2.83	7.12	9.2×10^−5^	1.38				87
iNOS	NOS2	6.37	82.91	1.7×10^−9^		1.11			100
p35	IL12A	−1.58	0.34	0.186					100
CCL3	CCL3	2.46	5.52	6.2×10^−3^			1.07		
AREG	AREG	2.05	4.15	1.2×10^−3^	2.54		2.15	1.35	7
CCL20	CCL20	3.08	8.45	4.7×10^−5^	2.79		3.64	2.90	67
IL19	IL19	5.45	43.57	2.0×10^−5^	1.43		2.21	1.88	8
IL1β	IL1B	3.66	12.66	6.8×10^−6^	2.61		1.97	1.11	83
K16	KRT16	5.10	34.34	8.3×10^−9^	3.68	1.99	4.57	4.11	0
MX1	MX1	3.56	11.83	4.1×10^−5^	3.23	3.24	3.24	2.32	0
IL8	IL8	6.19	72.82	1.0×10^−7^	5.85	2.96	4.67	4.03	47
β Defensin	DEFB4	4.12	17.42	6.5×10^−8^	5.96	1.96	7.34	7.07	1

1 The largest fold change (FCH) was reported when there were several probesets representing the same gene.

2 Percentage of samples with low intensity as defined by having expression smaller that 4.

### Correlation of other published psoriasis transcriptomes compared to our transcriptome using Gene Set Enrichment Analysis (GSEA)

A more robust approach beyond a Venn diagram was required to overcome these limitations. We propose to use a Gene-Sets approach to analyze how published DEG rank in our fold change data. This reduces the bias due to preprocessing steps, statistical protocols and stringency of cut-offs. This approach is successfully used in the Connectivity Map [27], a pattern recognition instance that correlates disease signatures (based on gene expression of any platform) with drugs. The idea is to use the GSEA framework [10] by considering the published DEG as a pathway or gene set, and quantify how well the up (and down) regulated genes rank in the ordered fold change for all genes in our data. This will generate an ES for up-regulated genes and one for the down-regulated genes. The connectivity score (CS) can be used to give a measure of agreement between studies, by combining the two ES into one final value, as used in the connectivity map to rank drugs that better correlate with disease. A value of CS near 1 would indicate perfect agreement between a study list and our analysis, whereas 0 would indicate no agreement, and −1 a negative correlation.

**Figure 2:**
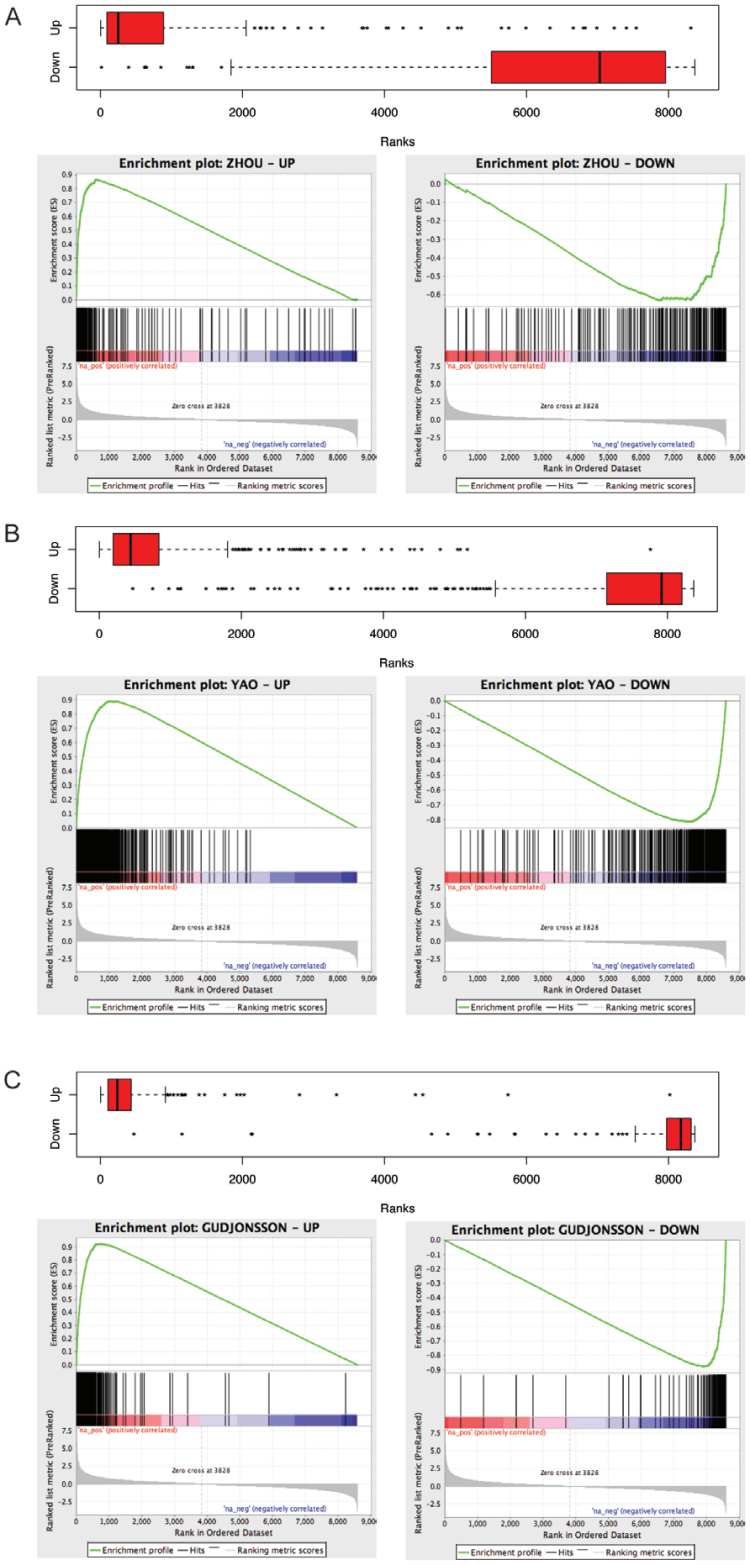
Rank of the published DEG lists in our fold change data. **A**. Rank of Zhou's genes in our data set ordered by fold change expression, with Enrichment plots for the up-regulated and downregulated genes data below. **B**. As A but for Yao's study. **C**. As A but for Gudjonsson's study. A detailed explanation of Enrichment plots can be found in Figure 1 of original Subramanian *et al* [10].

GSEA showed that there was highly significant enrichment of psoriasis DEGs, both up- and down-regulated genes, from the three studies compared to our data (Table 4). The GSEA plot for up and down-regulated genes in Zhou's, Yao's and Gudjonsson's lists is shown in Figure 2. P-values for ES and CS were calculated using 10,000 simulations. For Zhou's list, the ES for the up-regulated genes was 0.86 (p<0.0001) and −0.63 (p<0.0001) for the down-regulated genes (Figure 2A). The CS = 0.75 (p<0.0001) indicates a positive significant agreement between Zhou's signature and our data. For Yao's transcriptome (Figure 2B), the ES = 0.90 (p<0.0001) for the up-regulated genes and ES = −0.86 (p<0.0001) for the down–regulated genes, for CS = 0.88 (p<0.0001), which also indicates a positive significant agreement. For Gudjonsson's transcriptome (Figure 2C), the ES = 0.93 (p<0.0001) for the up-regulated genes and ES = −0.91 (p<0.0001) for the down–regulated genes, for CS = 0.92 (p<0.0001), which also indicates a positive significant agreement. In general, a better agreement was observed among up-regulated genes for all studies. In addition, Yao's transcriptome correlated better with our study than Zhou's, which is not surprising since the array series and the statistical protocols used in Yao's and ours were more similar than those in Zhou's.

**Table 4:** GESA analysis of published transcriptomes with our data.

Gene Set	No. of genes in pathway	ES	NES	FDR	CS
Gudjonsson - UP	386	0.93	4.25	<10^−4^	0.92
Gudjonsson - Down	153	−0.91	−3.77	<10^−4^	
Yao - UP	670	0.90	4.31	<10^−4^	0.88
Yao – Down	487	−0.86	−4.06	<10^−4^	
Zhou - UP	199	0.86	3.69	<10^−4^	0.75
Zhou - Down	227	−0.63	−2.71	<10^−4^	
SCC LSvsNL UP	859	0.69	3.37	<10^−4^	0.69
SCC LSvsNL Down	655	−0.70	−3.41	<10^−4^	
BCC LS vs Normal UP	191	0.38	1.61	<10^−4^	0.42
BCC LS vs Normal Down	326	−0.46	−2.09	<10^−4^	

ES: Enrichment Score; NES: Normalized Enrichment Score; FDR: False Discovery Rate.

We used the same approach to compare two published DEGs of other skin diseases produced by own group: squamous cell carcinoma (SCC) [28] and basal cell carcinoma (BCC) [29]. The CS for SCC was 0.69 and the CS of BCC was 0.42, considerably lower than in psoriasis (Table 4). This degree of enrichment is most likely reflects the origin of this data from our own lab, and the cutaneous nature of the specimens, as well as epidermal hyperproliferation and inflammation in all these three diseases.

## Discussion

Investigators might be surprised at the lack of overlap between DEG lists, as shown in the Venn diagram (Figure 1). However, if one considers all the variables involved in the four studies, summarized in Table 1, it is not that surprising. Although all the studies were conducted using Affymetrix platform, they used different array series, which may contribute to variability in results. Besides the obvious laboratory effect due to sample preparation, technician experience, equipment calibration, and the use of different preprocessing algorithms [30], alternative statistical tests and stringent cut-offs also contribute to different results [3], [31]. Measuring agreement of microarray studies by overlap of DEG lists generated by individual studies has been largely criticized [5] because it is highly inconsistent, even in the presence of small variation in the data as in the case of technical replicates [32], [33]. A low overlap between DEG does not directly indicate low agreement between studies [3], [31], [33].

Here we present our new data on the psoriasis transcriptome from our patients, as well as a comparison of our data with three published DEG lists for psoriasis. We find only 164 genes in common for the four lists. However by changing the focus of the single-gene approach behind the intersection of DEG involved in psoriasis and using a gene set approach, a closer biological similarity between the studies is revealed. The extension of GSEA presented here enables us to see that cellular processes and molecular signature involved in psoriasis is very robust across the studies. In this paper, we extended the use of GSEA to compare new expression data with previously published DEG lists in order to validate psoriasis disease-related gene profiles. It is worth noting that this approach is applicable to expression data obtained through deep sequencing (potentially improving sensitivity for low abundance genes and cross-hybridization problems of current microarray technology). Moreover, this approach is easily extendable to other *omics* applications and more complex phenotypes. Since the method is based on ranking a list according to a phenotype, the ranked list can be derived from other measures besides gene expression fold changes from microarray chips or deep sequencing; such phenotype measures may include odd ratios of single nucleotide polymorphisms (SNPs), or a microRNA profile assay derived from microarray technologies or deep sequencing.

The use of GSEA as a gene set approach is not unique: extensions to GSEA [34] and other Gene Set methods and statistics have also been proposed, and could also be used to compare transcriptomes. Efron and Tibshirani [35] proposed the MaxMean statistic instead of the weighted Kolmogorov Smirnov statistics used in the classical GSEA. Dinu *et al* [36] extended the single gene analysis SAM and proposed SAM-GS. See [7], [37] for a comparative study of different gene set enrichment methods. Extensions other than the classical difference between two phenotypes have also been reported. For example, we used the time slope of a mixed effect model as a phenotype to evaluate the time-response of cytokines pathways to psoriasis treatment with TNF inhibitor [17].

In this report, we show an excellent and simple method for researchers seeking validation of their own expression data with published lists from different studies.

## Materials and Methods

### Patients

Twenty adult patients with moderate to severe psoriasis were treated with etanercept 50 mg subcutaneously twice weekly for 12 weeks (clinical trial no. NCT00116181). The clinical and histological response of patients in this trial was previously published [16]. The gene array was performed on samples from 15 sequential patients [17]. RT-PCR was performed on samples from all 20 patients.

### Ethics Statement

The clinical trial (no. NCT00116181) was conducted according to the principles expressed in the Declaration of Helsinki and informed consent for their information to be stored in the hospital database and used for research was obtained from all patients in written form. This research was conducted under protocol JKR-0542 approved by the Rockefeller University Institutional Review Board.

### Sample Preparation and Hybridization

RNA was extracted from skin biopsies and hybridized to Affymetrix hgu133a2 chips as described in [17].

### RTPCR

Primers and Probes for TaqMan RT-PCR assays had been previously described [16]. New primers used in this study were CCL3 (Hs00234142_m1), CCL4 (Hs99999148_m1), AREG (Hs00950669_m1), IL-19 (Hs00604657_m1). All assays were obtained from Applied Biosystems. Data was normalized using human acidic ribosomal protein as a housekeeping gene [16].

### Statistical Analysis

Expression values were obtained using *gcrma* algorithm. Samples were filtered for unreliable low expression value and low variability. To compare lesional with non-lesional values, the moderated t-test available at *limma* package was used. P-values were adjusted for multiple hypothesis correction using the Benjamini-Hochberg approach, which controls the false discovery rate (FDR). Probesets with FDR<0.05 and more than 2 fold change (FCH) were considered differentially expressed. All analysis was carried out using R programming language (www.R-project.org) and Bioconductor packages (www.bioconductor.org).

Annotations were obtained by using Bioconductor hgu133a2.db and hgu133plus2.db packages version 2.3.5. Mappings were based on ENTREZ identifiers, provided by Entrez Gene fftp://ftp.ncbi.nlm.nih.gov/gene/DATA with a data stamp of Sept 1, 2009.

Gene Set Enrichment Analysis (GSEA) was conducted using GSEA software [18].

### Data Repository

This data has been deposited at the public repository Gene Expression Omnibus (GEO) (http://www.ncbi.nlm.nih.gov/geo/) with accession number GSE11903.

## Supporting Information

Table S1 DEG genes identified in this study (FDR<0.05, FCH>2).
(0.52 MB PDF)

Table S2 Genes consistently identified by the four studies.
(0.08 MB PDF)
