# Supplementary material for: Evaluation of the Psoriasis Transcriptome across Different Studies by Gene Set Enrichment Analysis (GSEA)
Source: PLoS One. 2010 Apr 20;5(4):e10247. doi: 10.1371/journal.pone.0010247 (PMC2857878; doi:10.1371/journal.pone.0010247)
Supplement: Table S1 — DEG genes identified in this study (FDR<0.05, FCH>2). (0.52 MB PDF) [file pone.0010247.s001.pdf]

	Probe	Symbol	Description	lgFCH	FCH	p	FDR		ENTREZ	UniGene
1	205863_at	S100A12	S100 calcium binding protein A12	7.58	191.11	4.02E-14	1.05E-11		6283	Hs.19413
2	205513_at	TCN1	transcobalamin I (vitamin B12 binding pr	7.45	174.63	3.07E-11	1.45E-09		6947	Hs.2012
3	204733_at	KLK6	kallikrein-related peptidase 6	6.63	98.77	6.19E-11	2.62E-09		5653	Hs.79361
4	210663_s_at	KYNU	kynureninase (L-kynurenine hydrolase)	6.16	71.36	5.28E-18	1.70E-14		8942	Hs.470126
5	211906_s_at	SERPINB4	serpin peptidase inhibitor, clade B (ovalb	6.09	68	1.45E-10	5.18E-09		6318	Hs.123035
6	217388_s_at	KYNU	kynureninase (L-kynurenine hydrolase)	6.04	65.62	4.60E-18	1.70E-14		8942	Hs.470126
7	207356_at	DEFB4	defensin, beta 4	5.96	62.12	3.19E-09	6.50E-08		1673	Hs.105924
8	219554_at	RHCG	Rh family, C glycoprotein	5.9	59.74	1.77E-11	9.35E-10		51458	Hs.459284
9	202859_x_at	IL8	interleukin 8	5.85	57.72	7.27E-09	1.29E-07		3576	Hs.624
10	207367_at	ATP12A	ATPase, H+/K+ transporting, nongastric,	5.71	52.46	1.79E-15	1.27E-12		479	Hs.147111
11	212531_at	LCN2	lipocalin 2	5.65	50.14	3.18E-12	2.48E-10		3934	Hs.204238
12	205660_at	OASL	2'-5'-oligoadenylate synthetase-like	5.65	50.11	1.17E-11	6.85E-10		8638	Hs.118633
13	206561_s_at	AKR1B10	aldo-keto reductase family 1, member B	5.25	37.96	3.56E-15	1.83E-12		57016	Hs.116724
14	220664_at	SPRR2C	small proline-rich protein 2C (pseudoger	5.2	36.68	2.75E-14	7.69E-12		6702	Hs.2421
15	211362_s_at	SERPINB13	serpin peptidase inhibitor, clade B (ovalb	5.19	36.49	7.52E-17	1.07E-13		5275	Hs.241407
16	210413_x_at			5.16	35.73	1.03E-11	6.33E-10			
17	203691_at	PI3	peptidase inhibitor 3, skin-derived	5.11	34.46	8.93E-12	5.63E-10		5266	Hs.112341
18	207602_at	TMPRSS11D	transmembrane protease, serine 11D	5.02	32.5	2.29E-12	1.94E-10		9407	Hs.132195
19	210797_s_at	OASL	2'-5'-oligoadenylate synthetase-like	5	31.92	4.52E-12	3.21E-10		8638	Hs.118633
20	216258_s_at	SERPINB13	serpin peptidase inhibitor, clade B (ovalb	4.97	31.29	1.90E-18	1.22E-14		5275	Hs.241407
21	205783_at	KLK13	kallikrein-related peptidase 13	4.96	31.04	2.24E-14	6.65E-12		26085	Hs.165296
22	41469_at	PI3	peptidase inhibitor 3, skin-derived	4.76	27.05	6.35E-11	2.67E-09		5266	Hs.112341
23	33646_g_at	GM2A	GM2 ganglioside activator	4.68	25.68	1.00E-12	1.01E-10		2760	Hs.483873
24	215891_s_at	GM2A	GM2 ganglioside activator	4.66	25.24	1.78E-12	1.58E-10		2760	Hs.483873
25	209719_x_at	SERPINB3	serpin peptidase inhibitor, clade B (ovalb	4.62	24.67	1.19E-13	2.07E-11		6317	Hs.227948
26	204385_at	KYNU	kynureninase (L-kynurenine hydrolase)	4.56	23.61	1.13E-13	2.07E-11		8942	Hs.470126
27	202018_s_at	LTF	lactotransferrin	4.56	23.59	1.15E-07	1.27E-06		4057	Hs.529517
28	204972_at	OAS2	2'-5'-oligoadenylate synthetase 2, 69/71I	4.5	22.58	1.15E-11	6.80E-10		4939	Hs.414332
29	217315_s_at	KLK13	kallikrein-related peptidase 13	4.49	22.52	7.70E-13	8.25E-11		26085	Hs.165296
30	219403_s_at	HPSE	heparanase	4.43	21.62	1.71E-13	2.76E-11		10855	Hs.44227
31	220322_at	IL1F9	interleukin 1 family, member 9	4.37	20.73	1.50E-12	1.38E-10		56300	Hs.211238
32	209720_s_at	SERPINB3	serpin peptidase inhibitor, clade B (ovalb	4.37	20.63	9.15E-12	5.71E-10		6317	Hs.227948
33	211788_s_at	TREX2	three prime repair exonuclease 2	4.28	19.39	1.88E-13	2.95E-11		11219	Hs.644635
34	213060_s_at	CHI3L2	chitinase 3-like 2	4.27	19.31	1.24E-11	7.05E-10		1117	Hs.514840
35	221107_at	CHRNA9	cholinergic receptor, nicotinic, alpha 9	4.11	17.27	1.89E-16	1.73E-13		55584	Hs.272278
36	219352_at	HERC6	hect domain and RLD 6	4.08	16.85	2.34E-10	7.83E-09		55008	Hs.529317
37	214490_at	ARSF	arylsulfatase F	4.05	16.55	1.97E-14	6.17E-12		416	Hs.101674
38	203535_at	S100A9	S100 calcium binding protein A9	3.9	14.88	1.46E-12	1.38E-10		6280	Hs.112405
39	219850_s_at	EHF	ets homologous factor	3.77	13.64	4.29E-12	3.08E-10		26298	Hs.653859
40	215808_at	KLK10	kallikrein-related peptidase 10	3.74	13.4	2.74E-12	2.22E-10		5655	Hs.275464
41	202869_at	OAS1	2',5'-oligoadenylate synthetase 1, 40/46k	3.7	12.97	9.00E-12	5.65E-10		4938	Hs.524760
42	209800_at	KRT16	keratin 16	3.68	12.86	3.39E-11	1.58E-09		3868	Hs.655160
43	218810_at	ZC3H12A	zinc finger CCCH-type containing 12A	3.68	12.86	1.17E-13	2.07E-11		80149	Hs.656294
44	203779_s_at	MPZL2	myelin protein zero-like 2	3.65	12.58	2.01E-13	3.04E-11		10205	Hs.116651
45	204470_at	CXCL1	chemokine (C-X-C motif) ligand 1 (melar	3.6	12.13	6.90E-06	4.11E-05		2919	Hs.789
46	211506_s_at	IL8	interleukin 8	3.57	11.86	7.63E-06	4.47E-05		3576	Hs.624
47	213797_at	RSAD2	radical S-adenosyl methionine domain c	3.48	11.12	9.52E-07	7.68E-06		91543	Hs.17518
48	219795_at	SLC6A14	solute carrier family 6 (amino acid transp	3.46	10.98	4.92E-10	1.41E-08		11254	Hs.522109
49	215223_s_at	SOD2	superoxide dismutase 2, mitochondrial	3.44	10.88	5.38E-11	2.32E-09		6648	Hs.487046
50	205990_s_at	WNT5A	wingless-type MMTV integration site fam	3.44	10.86	1.14E-10	4.31E-09		7474	Hs.696364
51	203699_s_at	DIO2	deiodinase, iodothyronine, type II	3.43	10.81	2.05E-10	7.00E-09		1734	Hs.202354
52	203127_s_at	SPTLC2	serine palmitoyltransferase, long chain b	3.43	10.8	8.22E-14	1.72E-11		9517	Hs.435661
53	215977_x_at	GK	glycerol kinase	3.39	10.51	1.31E-11	7.36E-10		2710	Hs.1466
54	216202_s_at	SPTLC2	serine palmitoyltransferase, long chain b	3.39	10.5	2.27E-14	6.65E-12		9517	Hs.435661
55	206134_at	ADAMDEC1	ADAM-like, decysin 1	3.39	10.45	5.23E-10	1.47E-08		27299	Hs.521459
56	204439_at	IFI44L	interferon-induced protein 44-like	3.38	10.44	4.99E-08	6.38E-07		10964	Hs.389724
57	206133_at	XAF1	XIAP associated factor 1	3.35	10.18	1.97E-13	3.01E-11		54739	Hs.441975
58	209727_at	GM2A	GM2 ganglioside activator	3.34	10.15	1.05E-11	6.45E-10		2760	Hs.483873
59	205552_s_at	OAS1	2',5'-oligoadenylate synthetase 1, 40/46k	3.34	10.11	7.07E-12	4.59E-10		4938	Hs.524760
60	209773_s_at	RRM2	ribonucleotide reductase M2	3.33	10.03	1.30E-10	4.77E-09		6241	Hs.226390
61	204858_s_at	TYMP	thymidine phosphorylase	3.31	9.92	1.50E-12	1.38E-10		1890	Hs.592212
62	204751_x_at	DSC2	desmocollin 2	3.27	9.64	1.73E-11	9.24E-10		1824	Hs.95612
63	219691_at	SAMD9	sterile alpha motif domain containing 9	3.24	9.46	5.04E-11	2.23E-09		54809	Hs.65641
64	202086_at	MX1	myxovirus (influenza virus) resistance 1,	3.23	9.36	1.14E-11	6.79E-10		4599	Hs.517307
65	214453_s_at	IFI44	interferon-induced protein 44	3.22	9.29	8.55E-10	2.20E-08		10561	Hs.82316

	Probe	Symbol	Description	lgFCH	FCH	p	FDR		ENTREZ	UniGene
66	203946_s_at	ARG2	arginase, type II	3.18	9.07	1.43E-09	3.35E-08		384	Hs.708024
67	204415_at	IFI6	interferon, alpha-inducible protein 6	3.18	9.05	1.30E-09	3.10E-08		2537	Hs.511731, Hs.
68	209969_s_at	STAT1	signal transducer and activator of transc	3.16	8.97	1.66E-11	8.91E-10		6772	Hs.642990, Hs.
69	209772_s_at	CD24	CD24 molecule	3.15	8.87	4.50E-16	3.62E-13		1E+08	Hs.644105, Hs.
70	219722_s_at	GDPD3	glycerophosphodiester phosphodiestera	3.14	8.83	3.46E-12	2.65E-10		79153	Hs.289015
71	207387_s_at	GK	glycerol kinase	3.14	8.83	6.37E-13	7.30E-11		2710	Hs.1466
72	220658_s_at	ARNTL2	aryl hydrocarbon receptor nuclear transl	3.11	8.63	7.16E-15	2.88E-12		56938	Hs.434269, Hs.
73	217028_at	CXCR4	chemokine (C-X-C motif) receptor 4	3.05	8.28	3.30E-10	1.04E-08		7852	Hs.593413
74	204750_s_at	DSC2	desmocollin 2	3.01	8.07	1.13E-08	1.87E-07		1824	Hs.95612
75	205844_at	VNN1	vanin 1	3.01	8.07	1.44E-10	5.18E-09		8876	Hs.12114
76	221698_s_at	CLEC7A	C-type lectin domain family 7, member A	3	7.98	4.90E-15	2.25E-12		64581	Hs.143929
77	210119_at	KCNJ15	potassium inwardly-rectifying channel, su	2.99	7.96	6.07E-15	2.69E-12		3772	Hs.411299
78	218400_at	OAS3	2'-5'-oligoadenylate synthetase 3, 100kD	2.99	7.94	6.12E-12	4.08E-10		4940	Hs.528634
79	206628_at	SLC5A1	solute carrier family 5 (sodium/glucose c	2.97	7.84	1.19E-13	2.07E-11		6523	Hs.1964
80	203234_at	UPP1	uridine phosphorylase 1	2.96	7.8	1.30E-11	7.36E-10		7378	Hs.488240
81	201860_s_at	PLAT	plasminogen activator, tissue	2.93	7.61	4.47E-06	2.86E-05		5327	Hs.491582
82	202134_s_at	WWTR1	WW domain containing transcription reg	2.93	7.61	8.68E-12	5.53E-10		25937	Hs.719249
83	205483_s_at	ISG15	ISG15 ubiquitin-like modifier	2.92	7.56	7.01E-08	8.30E-07		9636	Hs.458485
84	208539_x_at			2.91	7.52	3.69E-13	4.80E-11			
85	209514_s_at	RAB27A	RAB27A, member RAS oncogene family	2.91	7.51	7.69E-14	1.68E-11		5873	Hs.654978
86	211361_s_at	SERPINB13	serpin peptidase inhibitor, clade B (ovalb	2.9	7.44	1.41E-12	1.37E-10		5275	Hs.241407
87	219270_at	CHAC1	ChaC, cation transport regulator homolo	2.88	7.37	6.26E-09	1.13E-07		79094	Hs.155569
88	200796_s_at	MCL1	myeloid cell leukemia sequence 1 (BCL2	2.88	7.35	1.22E-09	2.95E-08		4170	Hs.719112
89	214059_at	IFI44	interferon-induced protein 44	2.86	7.24	1.50E-08	2.36E-07		10561	Hs.82316
90	220187_at	STEAP4	STEAP family member 4	2.85	7.19	1.34E-09	3.20E-08		79689	Hs.521008
91	204580_at	MMP12	matrix metallopeptidase 12 (macrophage	2.83	7.14	5.23E-05	0.0002311		4321	Hs.1695
92	213425_at	WNT5A	wingless-type MMTV integration site fam	2.83	7.1	1.76E-10	6.10E-09		7474	Hs.696364
93	216834_at	RGS1	regulator of G-protein signaling 1	2.83	7.09	2.38E-08	3.47E-07		5996	Hs.75256
94	211429_s_at	SERPINA1	serpin peptidase inhibitor, clade A (alpha	2.82	7.05	5.33E-10	1.50E-08		5265	Hs.525557
95	205476_at	CCL20	chemokine (C-C motif) ligand 20	2.79	6.91	4.50E-06	2.87E-05		6364	Hs.75498
96	215150_at	YOD1	YOD1 OTU deubiquinating enzyme 1 ho	2.77	6.8	8.30E-07	6.84E-06		55432	Hs.567533, Hs.
97	221305_s_at			2.76	6.76	2.44E-07	2.38E-06			
98	217272_s_at	SERPINB13	serpin peptidase inhibitor, clade B (ovalb	2.75	6.72	4.21E-16	3.61E-13		5275	Hs.241407
99	210138_at	RGS20	regulator of G-protein signaling 20	2.73	6.62	2.41E-13	3.48E-11		8601	Hs.368733
100	217167_x_at	GK	glycerol kinase	2.71	6.53	8.00E-09	1.40E-07		2710	Hs.1466
101	202376_at	SERPINA3	serpin peptidase inhibitor, clade A (alpha	2.69	6.47	3.38E-05	0.000159		12	Hs.534293, Hs.
102	217497_at	TYMP	thymidine phosphorylase	2.67	6.35	4.12E-12	2.99E-10		1890	Hs.592212
103	218960_at	TMPRSS4	transmembrane protease, serine 4	2.65	6.29	1.93E-09	4.30E-08		56649	Hs.161985
104	218888_s_at	NETO2	neuropilin (NRP) and tolloid (TLL)-like 2	2.64	6.25	1.52E-09	3.54E-08		81831	Hs.444046
105	201490_s_at	PPIF	peptidylprolyl isomerase F	2.64	6.24	1.54E-10	5.46E-09		10105	Hs.381072
106	39402_at	IL1B	interleukin 1, beta	2.61	6.12	2.58E-07	2.49E-06		3553	Hs.126256
107	203798_s_at	VSNL1	visinin-like 1	2.6	6.05	2.79E-12	2.24E-10		7447	Hs.444212
108	215966_x_at	GK3P	glycerol kinase 3 pseudogene	2.59	6.02	3.14E-13	4.21E-11		2713	Hs.654557
109	220330_s_at	SAMSN1	SAM domain, SH3 domain and nuclear l	2.57	5.93	2.00E-08	2.99E-07		64092	Hs.473341, Hs.
110	214446_at	ELL2	elongation factor, RNA polymerase II, 2	2.55	5.85	3.45E-15	1.83E-12		22936	Hs.192221, Hs.
111	214710_s_at	CCNB1	cyclin B1	2.55	5.84	1.91E-07	1.94E-06		891	Hs.23960
112	205239_at	AREG	amphiregulin	2.54	5.8	6.74E-09	1.21E-07		374	Hs.270833
113	203153_at	IFIT1	interferon-induced protein with tetratricop	2.53	5.77	6.93E-08	8.22E-07		3434	Hs.20315
114	208650_s_at	CD24	CD24 molecule	2.53	5.77	1.87E-15	1.27E-12		1E+08	Hs.644105, Hs.
115	208436_s_at	IRF7	interferon regulatory factor 7	2.52	5.75	2.26E-11	1.14E-09		3665	Hs.166120
116	203764_at	DLGAP5	discs, large (Drosophila) homolog-assoc	2.52	5.72	6.25E-08	7.64E-07		9787	Hs.77695
117	221765_at	UGCG	UDP-glucose ceramide glucosyltransfera	2.51	5.71	2.25E-09	4.91E-08		7357	Hs.304249, Hs.
118	210904_s_at	IL13RA1	interleukin 13 receptor, alpha 1	2.51	5.68	1.57E-12	1.43E-10		3597	Hs.496646
119	211834_s_at	TP63	tumor protein p63	2.5	5.67	9.52E-08	1.08E-06		8626	Hs.137569
120	202833_s_at	SERPINA1	serpin peptidase inhibitor, clade A (alpha	2.5	5.64	1.31E-08	2.11E-07		5265	Hs.525557
121	210652_s_at	TTC39A	tetratricopeptide repeat domain 39A	2.49	5.62	1.60E-11	8.67E-10		22996	Hs.112949
122	209921_at	SLC7A11	solute carrier family 7, (cationic amino ac	2.48	5.58	1.75E-08	2.68E-07		23657	Hs.390594
123	218384_at	CARHSP1	calcium regulated heat stable protein 1,	2.47	5.54	4.34E-14	1.12E-11		23589	Hs.632184
124	216841_s_at	SOD2	superoxide dismutase 2, mitochondrial	2.47	5.54	2.09E-09	4.60E-08		6648	Hs.487046
125	220249_at	HYAL4	hyaluronoglucosaminidase 4	2.46	5.51	2.21E-08	3.26E-07		23553	Hs.28673
126	210951_x_at	RAB27A	RAB27A, member RAS oncogene family	2.45	5.46	6.40E-11	2.68E-09		5873	Hs.654978
127	206008_at	TGM1	transglutaminase 1 (K polypeptide epide	2.45	5.46	8.24E-14	1.72E-11		7051	Hs.508950
128	210873_x_at	APOBEC3A	apolipoprotein B mRNA editing enzyme,	2.45	5.45	8.09E-06	4.70E-05		200315	Hs.348983
129	204995_at	CDK5R1	cyclin-dependent kinase 5, regulatory su	2.41	5.32	7.03E-11	2.87E-09		8851	Hs.500015
130	216316_x_at			2.41	5.32	1.13E-08	1.86E-07			

	Probe	Symbol	Description	lgFCH	FCH	p	FDR		ENTREZ	UniGene
131	219956_at	GALNT6	UDP-N-acetyl-alpha-D-galactosamine:po	2.4	5.28	3.37E-09	6.82E-08		11226	Hs.505575
132	203180_at	ALDH1A3	aldehyde dehydrogenase 1 family, meml	2.4	5.27	1.39E-05	7.40E-05		220	Hs.459538
133	212268_at	SERPINB1	serpin peptidase inhibitor, clade B (ovalb	2.36	5.13	1.99E-09	4.42E-08		1992	Hs.381167
134	211919_s_at	CXCR4	chemokine (C-X-C motif) receptor 4	2.35	5.1	2.47E-06	1.72E-05		7852	Hs.593413
135	32128_at	CCL18	chemokine (C-C motif) ligand 18 (pulmor	2.35	5.08	8.58E-05	0.0003555		6362	Hs.143961
136	214580_x_at			2.33	5.02	9.23E-10	2.35E-08			
137	207008_at	IL8RB	interleukin 8 receptor, beta	2.25	4.75	7.16E-14	1.61E-11		3579	Hs.846
138	209882_at	RIT1	Ras-like without CAAX 1	2.25	4.74	1.79E-09	4.08E-08		6016	Hs.491234
139	210087_s_at	MPZL1	myelin protein zero-like 1	2.24	4.73	2.19E-11	1.12E-09		9019	Hs.493919, Hs.
140	219684_at	RTP4	receptor (chemosensory) transporter pro	2.24	4.72	3.13E-07	2.95E-06		64108	Hs.43388
141	202575_at	CRABP2	cellular retinoic acid binding protein 2	2.24	4.71	2.71E-13	3.78E-11		1382	Hs.405662
142	202411_at	IFI27	interferon, alpha-inducible protein 27	2.24	4.71	1.25E-12	1.23E-10		3429	Hs.532634
143	211612_s_at	IL13RA1	interleukin 13 receptor, alpha 1	2.23	4.71	3.06E-10	9.75E-09		3597	Hs.496646
144	214549_x_at	SPRR1A	small proline-rich protein 1A	2.22	4.67	8.29E-14	1.72E-11		6698	Hs.46320
145	214226_at	POL3S	polyserase 3	2.21	4.62	8.49E-08	9.77E-07		339105	Hs.569575
146	218990_s_at	SPRR3	small proline-rich protein 3	2.21	4.61	6.19E-06	3.77E-05		6707	Hs.139322
147	202831_at	GPX2	glutathione peroxidase 2 (gastrointestina	2.2	4.59	2.72E-08	3.88E-07		2877	Hs.2704
148	202870_s_at	CDC20	cell division cycle 20 homolog (S. cerevis	2.19	4.58	1.29E-06	9.94E-06		991	Hs.524947
149	205681_at	BCL2A1	BCL2-related protein A1	2.18	4.55	1.20E-06	9.38E-06		597	Hs.227817
150	209792_s_at	KLK10	kallikrein-related peptidase 10	2.16	4.47	1.93E-13	2.99E-11		5655	Hs.275464
151	218943_s_at	DDX58	DEAD (Asp-Glu-Ala-Asp) box polypeptid	2.16	4.47	9.63E-09	1.65E-07		23586	Hs.190622
152	211561_x_at	MAPK14	mitogen-activated protein kinase 14	2.14	4.42	1.63E-07	1.69E-06		1432	Hs.485233
153	35820_at	GM2A	GM2 ganglioside activator	2.14	4.41	1.09E-14	3.80E-12		2760	Hs.483873
154	205554_s_at	DNASE1L3	deoxyribonuclease I-like 3	2.14	4.4	3.96E-09	7.69E-08		1776	Hs.476453
155	204941_s_at	ALDH3B2	aldehyde dehydrogenase 3 family, meml	2.14	4.4	1.33E-07	1.42E-06		222	Hs.87539
156	220865_s_at	PDSS1	prenyl (decaprenyl) diphosphate synthas	2.14	4.4	4.04E-08	5.33E-07		23590	Hs.558468
157	218340_s_at	UBA6	ubiquitin-like modifier activating enzyme	2.14	4.4	3.10E-06	2.09E-05		55236	Hs.719086
158	211016_x_at	HSPA4	heat shock 70kDa protein 4	2.13	4.39	2.18E-14	6.65E-12		3308	Hs.90093
159	205729_at	OSMR	oncostatin M receptor	2.13	4.38	8.47E-09	1.47E-07		9180	Hs.120658
160	201489_at	PPIF	peptidylprolyl isomerase F	2.13	4.38	1.33E-14	4.40E-12		10105	Hs.381072
161	214007_s_at	TWF1	twinfilin, actin-binding protein, homolog 1	2.13	4.38	1.25E-06	9.67E-06		5756	Hs.189075
162	209774_x_at	CXCL2	chemokine (C-X-C motif) ligand 2	2.13	4.38	2.90E-05	0.00014		2920	Hs.75765
163	208079_s_at	AURKA	aurora kinase A	2.12	4.36	2.93E-09	6.08E-08		6790	Hs.250822
164	214681_at	GK	glycerol kinase	2.12	4.35	1.66E-10	5.78E-09		2710	Hs.1466
165	209126_x_at	KRT6B	keratin 6B	2.11	4.31	5.31E-11	2.30E-09		3854	Hs.709235
166	209546_s_at	APOL1	apolipoprotein L, 1	2.11	4.31	4.78E-09	9.09E-08		8542	Hs.114309
167	210915_x_at			2.11	4.3	5.44E-05	0.0002391			
168	209125_at	KRT6A	keratin 6A	2.1	4.27	9.81E-11	3.86E-09		3853	Hs.700779
169	201195_s_at	SLC7A5	solute carrier family 7 (cationic amino ac	2.1	4.27	1.26E-07	1.36E-06		8140	Hs.513797
170	205157_s_at	KRT17	keratin 17	2.09	4.27	1.51E-08	2.37E-07		3872	Hs.2785
171	209626_s_at	OSBPL3	oxysterol binding protein-like 3	2.09	4.25	5.08E-07	4.48E-06		26031	Hs.520259
172	217192_s_at	PRDM1	PR domain containing 1, with ZNF doma	2.08	4.24	2.89E-15	1.77E-12		639	Hs.436023
173	207018_s_at	RAB27B	RAB27B, member RAS oncogene family	2.07	4.21	5.18E-11	2.28E-09		5874	Hs.25318
174	218900_at	CNNM4	cyclin M4	2.07	4.2	3.21E-12	2.49E-10		26504	Hs.175043
175	205822_s_at	HMGCS1	3-hydroxy-3-methylglutaryl-Coenzyme A	2.07	4.2	2.43E-05	0.0001202		3157	Hs.397729
176	200730_s_at	PTP4A1	protein tyrosine phosphatase type IVA, n	2.06	4.18	4.13E-06	2.67E-05		7803	Hs.227777, Hs.
177	205241_at	SCO2	SCO cytochrome oxidase deficient homo	2.06	4.17	7.85E-10	2.05E-08		9997	Hs.718545
178	204351_at	S100P	S100 calcium binding protein P	2.06	4.16	1.72E-06	1.26E-05		6286	Hs.2962
179	222223_s_at	IL1F5	interleukin 1 family, member 5 (delta)	2.05	4.14	3.20E-13	4.24E-11		26525	Hs.516301
180	210449_x_at	MAPK14	mitogen-activated protein kinase 14	2.03	4.09	1.08E-08	1.81E-07		1432	Hs.485233
181	219799_s_at	DHRS9	dehydrogenase/reductase (SDR family)	2.02	4.06	8.54E-10	2.20E-08		10170	Hs.179608
182	219630_at	PDZK1IP1	PDZK1 interacting protein 1	2.01	4.03	1.16E-13	2.07E-11		10158	Hs.431099
183	212737_at	GM2A	GM2 ganglioside activator	2	4	3.47E-15	1.83E-12		2760	Hs.483873
184	203214_x_at	CDC2	cell division cycle 2, G1 to S and G2 to M	2	3.99	3.14E-06	2.11E-05		983	Hs.334562
185	214581_x_at	TNFRSF21	tumor necrosis factor receptor superfam	2	3.99	3.89E-09	7.59E-08		27242	Hs.443577
186	221962_s_at	UBE2H	ubiquitin-conjugating enzyme E2H (UBC	1.99	3.97	5.35E-11	2.31E-09		7328	Hs.643548
187	205246_at	PEX13	peroxisomal biogenesis factor 13	1.98	3.95	1.34E-10	4.89E-09		5194	Hs.161377
188	209093_s_at			1.98	3.93	2.19E-12	1.91E-10			
189	209723_at	SERPINB9	serpin peptidase inhibitor, clade B (ovalb	1.97	3.92	1.19E-05	6.55E-05		5272	Hs.104879
190	205569_at	LAMP3	lysosomal-associated membrane proteir	1.97	3.92	3.54E-07	3.30E-06		27074	Hs.518448
191	218295_s_at	NUP50	nucleoporin 50kDa	1.97	3.91	5.04E-10	1.44E-08		10762	Hs.715672
192	202626_s_at	LYN	v-yes-1 Yamaguchi sarcoma viral relatec	1.96	3.9	8.15E-08	9.41E-07		4067	Hs.699154
193	206643_at	HAL	histidine ammonia-lyase	1.96	3.89	1.79E-16	1.73E-13		3034	Hs.190783
194	205242_at	CXCL13	chemokine (C-X-C motif) ligand 13	1.95	3.86	0.00071663	0.0022118		10563	Hs.100431
195	206303_s_at	NUDT4	nudix (nucleoside diphosphate linked mo	1.95	3.85	5.12E-10	1.44E-08		11163	Hs.506325

	Probe	Symbol	Description	lgFCH	FCH	p	FDR		ENTREZ	UniGene
196	217173_s_at	LDLR	low density lipoprotein receptor	1.94	3.84	8.14E-08	9.41E-07		3949	Hs.213289, Hs.
197	205114_s_at			1.94	3.83	0.00257459	0.0066951			
198	210834_s_at	PTGER3	prostaglandin E receptor 3 (subtype EP3	1.93	3.81	1.19E-07	1.29E-06		5733	Hs.445000
199	201695_s_at	NP	nucleoside phosphorylase	1.93	3.81	1.88E-08	2.85E-07		4860	Hs.75514
200	218817_at	SPCS3	signal peptidase complex subunit 3 hom	1.92	3.79	2.19E-07	2.18E-06		60559	Hs.42194
201	203256_at	CDH3	cadherin 3, type 1, P-cadherin (placenta	1.92	3.79	5.18E-11	2.28E-09		1001	Hs.191842
202	214697_s_at	ROD1	ROD1 regulator of differentiation 1 (S. po	1.92	3.78	2.76E-06	1.90E-05		9991	Hs.269988
203	204533_at	CXCL10	chemokine (C-X-C motif) ligand 10	1.92	3.77	0.00033084	0.0011274		3627	Hs.632586
204	219225_at	PGBD5	piggyBac transposable element derived	1.91	3.77	3.78E-06	2.47E-05		79605	Hs.520463
205	208126_s_at	CYP2C18	cytochrome P450, family 2, subfamily C,	1.91	3.75	3.35E-06	2.23E-05		1562	Hs.511872
206	213562_s_at	SQLE	squalene epoxidase	1.9	3.73	3.42E-09	6.91E-08		6713	Hs.71465
207	206553_at	OAS2	2'-5'-oligoadenylate synthetase 2, 69/71	1.89	3.71	3.05E-08	4.25E-07		4939	Hs.414332
208	209555_s_at	CD36	CD36 molecule (thrombospondin recepto	1.89	3.71	7.77E-07	6.46E-06		948	Hs.120949
209	216244_at	IL1RN	interleukin 1 receptor antagonist	1.89	3.7	1.47E-08	2.33E-07		3557	Hs.81134
210	220318_at	EPN3	epsin 3	1.88	3.68	5.03E-13	6.17E-11		55040	Hs.670090
211	212902_at	SEC24A	SEC24 family, member A (S. cerevisiae)	1.88	3.67	1.42E-08	2.25E-07		10802	Hs.595540
212	213696_s_at	MED8	mediator complex subunit 8	1.88	3.67	6.82E-10	1.86E-08		112950	Hs.301756
213	202357_s_at			1.87	3.66	5.21E-08	6.59E-07			
214	220944_at	PGLYRP4	peptidoglycan recognition protein 4	1.87	3.65	7.95E-10	2.07E-08		57115	Hs.58356
215	206004_at	TGM3	transglutaminase 3 (E polypeptide, prote	1.87	3.65	2.30E-09	5.00E-08		7053	Hs.2022
216	219358_s_at	ADAP2	ArfGAP with dual PH domains 2	1.87	3.64	3.28E-14	8.79E-12		55803	Hs.514063
217	204641_at	NEK2	NIMA (never in mitosis gene a)-related k	1.86	3.62	1.30E-06	1.00E-05		4751	Hs.153704
218	203148_s_at	TRIM14	tripartite motif-containing 14	1.86	3.62	2.25E-11	1.14E-09		9830	Hs.575631
219	217521_at			1.85	3.61	1.27E-10	4.71E-09			
220	203915_at	CXCL9	chemokine (C-X-C motif) ligand 9	1.85	3.6	0.00462098	0.011133		4283	Hs.77367
221	210852_s_at	AASS	aminoadipate-semialdehyde synthase	1.84	3.58	9.92E-14	1.96E-11		10157	Hs.156738
222	209456_s_at	FBXW11	F-box and WD repeat domain containing	1.84	3.57	2.21E-09	4.82E-08		23291	Hs.484138
223	219099_at	C12orf5	chromosome 12 open reading frame 5	1.83	3.55	2.90E-10	9.43E-09		57103	Hs.504545
224	214168_s_at	TJP1	tight junction protein 1 (zona occludens	1.82	3.53	1.40E-08	2.23E-07		7082	Hs.719113
225	211796_s_at			1.82	3.53	1.76E-06	1.29E-05			
226	217739_s_at	NAMPT	nicotinamide phosphoribosyltransferase	1.82	3.52	1.28E-10	4.72E-09		10135	Hs.489615
227	218454_at	PLBD1	phospholipase B domain containing 1	1.81	3.52	1.76E-16	1.73E-13		79887	Hs.131933
228	201884_at	CEACAM5	carcinoembryonic antigen-related cell ad	1.81	3.51	1.01E-05	5.63E-05		1048	Hs.709196
229	210357_s_at	SMOX	spermine oxidase	1.81	3.51	3.50E-11	1.61E-09		54498	Hs.433337
230	206211_at	SELE	selectin E	1.8	3.49	4.61E-05	0.000207		6401	Hs.82848
231	213796_at	SPRR1A	small proline-rich protein 1A	1.8	3.49	6.18E-09	1.12E-07		6698	Hs.46320
232	204747_at	IFIT3	interferon-induced protein with tetratrico	1.8	3.49	1.43E-07	1.52E-06		3437	Hs.714337
233	219369_s_at	OTUB2	OTU domain, ubiquitin aldehyde binding	1.79	3.46	6.13E-11	2.60E-09		78990	Hs.278815
234	201888_s_at	IL13RA1	interleukin 13 receptor, alpha 1	1.79	3.46	1.46E-12	1.38E-10		3597	Hs.496646
235	205847_at	PRSS22	protease, serine, 22	1.78	3.44	1.54E-09	3.58E-08		64063	Hs.459709
236	209231_s_at	DCTN5	dynactin 5 (p25)	1.78	3.43	6.77E-10	1.85E-08		84516	Hs.435941
237	205916_at	S100A7	S100 calcium binding protein A7	1.78	3.43	1.06E-09	2.64E-08		6278	Hs.112408
238	209201_x_at	CXCR4	chemokine (C-X-C motif) receptor 4	1.78	3.43	4.91E-05	0.0002188		7852	Hs.593413
239	210833_at	PTGER3	prostaglandin E receptor 3 (subtype EP3	1.77	3.42	3.08E-08	4.29E-07		5733	Hs.445000
240	204034_at	ETHE1	ethylmalonic encephalopathy 1	1.77	3.41	7.56E-07	6.30E-06		23474	Hs.7486
241	217678_at	SLC7A11	solute carrier family 7, (cationic amino ac	1.77	3.41	9.35E-08	1.06E-06		23657	Hs.390594
242	210827_s_at	ELF3	E74-like factor 3 (ets domain transcriptio	1.77	3.4	1.11E-09	2.73E-08		1999	Hs.67928
243	215465_at	ABCA12	ATP-binding cassette, sub-family A (ABC	1.76	3.39	1.25E-11	7.06E-10		26154	Hs.134585
244	219555_s_at	CENPN	centromere protein N	1.76	3.38	6.59E-07	5.63E-06		55839	Hs.55028
245	208596_s_at			1.76	3.38	3.32E-07	3.12E-06			
246	204702_s_at	NFE2L3	nuclear factor (erythroid-derived 2)-like 3	1.75	3.37	1.06E-08	1.79E-07		9603	Hs.404741, Hs.
247	218349_s_at	ZWILCH	Zwilch, kinetochore associated, homolog	1.75	3.37	3.88E-09	7.59E-08		55055	Hs.21331
248	207381_at	ALOX12B	arachidonate 12-lipoxygenase, 12R type	1.75	3.37	4.02E-13	5.12E-11		242	Hs.136574
249	211075_s_at	CD47	CD47 molecule	1.75	3.37	2.78E-17	5.97E-14		961	Hs.446414
250	210164_at	GZMB	granzyme B (granzyme 2, cytotoxic T-lym	1.75	3.36	4.43E-05	0.0002004		3002	Hs.1051
251	210732_s_at	LGALS8	lectin, galactoside-binding, soluble, 8	1.75	3.36	6.78E-09	1.22E-07		3964	Hs.4082, Hs.70
252	213457_at	MFHAS1	malignant fibrous histiocytoma amplified	1.74	3.35	1.23E-13	2.09E-11		9258	Hs.379414
253	203780_at	MPZL2	myelin protein zero-like 2	1.74	3.35	1.39E-10	5.00E-09		10205	Hs.116651
254	206177_s_at	ARG1	arginase, liver	1.74	3.35	5.85E-11	2.49E-09		383	Hs.440934
255	208651_x_at	CD24	CD24 molecule	1.74	3.34	3.01E-14	8.24E-12		1E+08	Hs.644105, Hs.
256	205488_at	GZMA	granzyme A (granzyme 1, cytotoxic T-lym	1.73	3.33	4.03E-08	5.33E-07		3001	Hs.90708
257	215125_s_at			1.73	3.32	3.33E-05	0.000157			
258	202917_s_at	S100A8	S100 calcium binding protein A8	1.73	3.31	4.63E-10	1.34E-08		6279	Hs.416073
259	217785_s_at	YKT6	YKT6 v-SNARE homolog (S. cerevisiae)	1.73	3.31	2.97E-10	9.54E-09		10652	Hs.520794
260	202503_s_at	KIAA0101	KIAA0101	1.72	3.3	2.28E-07	2.24E-06		9768	Hs.81892

	Probe	Symbol	Description	lgFCH	FCH	p	FDR		ENTREZ	UniGene
261	210754_s_at	LYN	v-yes-1 Yamaguchi sarcoma viral related	1.72	3.29	4.40E-10	1.30E-08		4067	Hs.699154
262	201890_at	RRM2	ribonucleotide reductase M2	1.72	3.29	9.86E-06	5.53E-05		6241	Hs.226390
263	213523_at	CCNE1	cyclin E1	1.71	3.28	7.49E-10	1.98E-08		898	Hs.244723
264	204465_s_at	INA	internexin neuronal intermediate filamen	1.71	3.27	1.14E-10	4.31E-09		9118	Hs.500916
265	201130_s_at	CDH1	cadherin 1, type 1, E-cadherin (epithelial	1.71	3.27	6.67E-06	4.01E-05		999	Hs.461086
266	206667_s_at	SCAMP1	secretory carrier membrane protein 1	1.71	3.26	0.00057351	0.0018131		9522	Hs.482587
267	210608_s_at	FUT2	fucosyltransferase 2 (secretor status inc	1.7	3.25	4.59E-07	4.11E-06		2524	Hs.579928
268	202269_x_at	GBP1	guanylate binding protein 1, interferon-in	1.7	3.25	3.92E-10	1.19E-08		2633	Hs.62661
269	206113_s_at	RAB5A	RAB5A, member RAS oncogene family	1.69	3.24	2.77E-07	2.66E-06		5868	Hs.475663
270	206302_s_at			1.69	3.22	8.49E-10	2.19E-08			
271	216243_s_at	IL1RN	interleukin 1 receptor antagonist	1.68	3.21	8.07E-07	6.67E-06		3557	Hs.81134
272	210845_s_at	PLAUR	plasminogen activator, urokinase recepto	1.68	3.2	3.81E-06	2.49E-05		5329	Hs.466871
273	214076_at	GFOD2	glucose-fructose oxidoreductase domain	1.68	3.2	7.75E-10	2.03E-08		81577	Hs.461030, Hs.
274	202934_at	HK2	hexokinase 2	1.68	3.2	1.22E-13	2.09E-11		3099	Hs.406266, Hs.
275	208965_s_at	IFI16	interferon, gamma-inducible protein 16	1.67	3.18	2.06E-07	2.07E-06		3428	Hs.380250
276	202270_at	GBP1	guanylate binding protein 1, interferon-in	1.67	3.18	2.37E-08	3.47E-07		2633	Hs.62661
277	204601_at	N4BP1	NEDD4 binding protein 1	1.67	3.17	6.54E-13	7.30E-11		9683	Hs.511839
278	219978_s_at	NUSAP1	nucleolar and spindle associated protein	1.66	3.17	2.08E-06	1.49E-05		51203	Hs.615092
279	220528_at	VNN3	vanin 3	1.66	3.17	7.03E-06	4.17E-05		55350	Hs.183656
280	212022_s_at	MKI67	antigen identified by monoclonal antibod	1.66	3.16	4.53E-09	8.61E-08		4288	Hs.689823, Hs.
281	217763_s_at	RAB31	RAB31, member RAS oncogene family	1.66	3.16	5.68E-10	1.59E-08		11031	Hs.714730, Hs.
282	266_s_at	CD24	CD24 molecule	1.66	3.15	2.93E-13	4.01E-11		1E+08	Hs.644105, Hs.
283	204393_s_at	ACPP	acid phosphatase, prostate	1.65	3.14	4.97E-09	9.41E-08		55	Hs.433060
284	202604_x_at	ADAM10	ADAM metallopeptidase domain 10	1.65	3.13	7.62E-09	1.34E-07		102	Hs.578508
285	219209_at	IFIH1	interferon induced with helicase C doma	1.64	3.12	2.57E-08	3.69E-07		64135	Hs.163173
286	204170_s_at	CKS2	CDC28 protein kinase regulatory subuni	1.64	3.12	1.48E-05	7.84E-05		1164	Hs.83758
287	206932_at	CH25H	cholesterol 25-hydroxylase	1.64	3.11	1.91E-08	2.88E-07		9023	Hs.47357
288	212236_x_at	KRT17	keratin 17	1.64	3.11	5.33E-08	6.70E-07		3872	Hs.2785
289	214975_s_at	MTMR1	myotubularin related protein 1	1.64	3.11	1.58E-05	8.29E-05		8776	Hs.347187
290	204825_at	MELK	maternal embryonic leucine zipper kinas	1.64	3.11	4.40E-08	5.75E-07		9833	Hs.184339
291	219181_at	LIPG	lipase, endothelial	1.63	3.1	0.00573338	0.013405		9388	Hs.465102
292	37152_at	PPARD	peroxisome proliferator-activated recepto	1.63	3.1	2.19E-12	1.91E-10		5467	Hs.696032
293	219079_at	CYB5R4	cytochrome b5 reductase 4	1.63	3.09	3.99E-07	3.62E-06		51167	Hs.5741
294	214070_s_at	ATP10B	ATPase, class V, type 10B	1.63	3.09	5.82E-13	7.00E-11		23120	Hs.109358
295	219695_at	SMPD3	sphingomyelin phosphodiesterase 3, neu	1.63	3.09	6.40E-15	2.74E-12		55512	Hs.368421, Hs.
296	203740_at	MPHOSPH6	M-phase phosphoprotein 6	1.63	3.09	9.02E-15	3.41E-12		10200	Hs.344400
297	203499_at	EPHA2	EPH receptor A2	1.62	3.06	7.13E-09	1.27E-07		1969	Hs.171596
298	203665_at	HMOX1	heme oxygenase (decycling) 1	1.61	3.06	5.32E-13	6.45E-11		3162	Hs.517581
299	204475_at	MMP1	matrix metallopeptidase 1 (interstitial col	1.61	3.06	0.00016063	0.0006085		4312	Hs.83169
300	211801_x_at	MFN1	mitofusin 1	1.61	3.05	1.85E-07	1.89E-06		55669	Hs.478383
301	212021_s_at	MKI67	antigen identified by monoclonal antibod	1.6	3.04	2.03E-05	0.0001025		4288	Hs.689823, Hs.
302	209714_s_at	CDKN3	cyclin-dependent kinase inhibitor 3	1.6	3.03	0.00010593	0.0004237		1033	Hs.84113
303	205595_at	DSG3	desmoglein 3 (pemphigus vulgaris antige	1.6	3.03	1.45E-12	1.38E-10		1830	Hs.1925
304	218392_x_at	SFXN1	sideroflexin 1	1.6	3.02	1.03E-09	2.58E-08		94081	Hs.369440
305	207038_at	SLC16A6	solute carrier family 16, member 6 (mono	1.59	3.01	8.31E-07	6.85E-06		9120	Hs.42645
306	202446_s_at	PLSCR1	phospholipid scramblase 1	1.59	3.01	2.48E-09	5.30E-08		5359	Hs.130759
307	218542_at	CEP55	centrosomal protein 55kDa	1.59	3.01	2.39E-06	1.68E-05		55165	Hs.14559
308	205666_at	FMO1	flavin containing monooxygenase 1	1.59	3.01	8.48E-06	4.89E-05		2326	Hs.1424
309	217924_at	C6orf106	chromosome 6 open reading frame 106	1.59	3.01	6.25E-08	7.64E-07		64771	Hs.643498
310	218755_at	KIF20A	kinesin family member 20A	1.59	3	1.10E-08	1.83E-07		10112	Hs.718626
311	205170_at	STAT2	signal transducer and activator of transc	1.59	3	1.09E-06	8.64E-06		6773	Hs.530595
312	205067_at	IL1B	interleukin 1, beta	1.59	3	0.00011307	0.000448		3553	Hs.126256
313	204698_at	ISG20	interferon stimulated exonuclease gene	1.59	3	2.04E-05	0.0001033		3669	Hs.459265
314	201211_s_at	DDX3X	DEAD (Asp-Glu-Ala-Asp) box polypeptid	1.58	3	0.0001019	0.0004096		1654	Hs.719127
315	205829_at	HSD17B1	hydroxysteroid (17-beta) dehydrogenase	1.58	3	3.44E-05	0.0001617		3292	Hs.654385, Hs.
316	201048_x_at	RAB6A	RAB6A, member RAS oncogene family	1.58	2.99	2.84E-09	5.94E-08		5870	Hs.503222, Hs.
317	206166_s_at	CLCA2	chloride channel accessory 2	1.58	2.99	1.01E-14	3.72E-12		9635	Hs.241551
318	219095_at			1.58	2.99	1.22E-10	4.57E-09			
319	204638_at	ACP5	acid phosphatase 5, tartrate resistant	1.57	2.97	3.65E-12	2.73E-10		54	Hs.1211
320	201858_s_at	SRGN	serglycin	1.57	2.96	0.00019564	0.0007199		5552	Hs.1908
321	204162_at	NDC80	NDC80 homolog, kinetochore complex c	1.56	2.96	8.32E-07	6.85E-06		10403	Hs.414407
322	212769_at	TLE3	transducin-like enhancer of split 3 (E(sp1	1.56	2.96	6.41E-13	7.30E-11		7090	Hs.287362
323	216379_x_at	CD24	CD24 molecule	1.56	2.95	3.73E-15	1.85E-12		1E+08	Hs.644105, Hs.
324	210559_s_at	CDC2	cell division cycle 2, G1 to S and G2 to M	1.56	2.95	1.21E-06	9.43E-06		983	Hs.334562
325	213975_s_at	LYZ	lysozyme (renal amyloidosis)	1.56	2.95	8.84E-09	1.53E-07		4069	Hs.524579

	Probe	Symbol	Description	lgFCH	FCH	p	FDR		ENTREZ	UniGene
326	213572_s_at	SERPINB1	serpin peptidase inhibitor, clade B (ovalb	1.56	2.94	1.16E-06	9.11E-06		1992	Hs.381167
327	205724_at	PKP1	plakophilin 1 (ectodermal dysplasia/skin	1.55	2.93	4.50E-05	0.0002029		5317	Hs.497350
328	216615_s_at	HTR3A	5-hydroxytryptamine (serotonin) recepto	1.55	2.93	0.00105817	0.0031157		3359	Hs.413899
329	218856_at	TNFRSF21	tumor necrosis factor receptor superfam	1.54	2.91	4.51E-11	2.02E-09		27242	Hs.443577
330	203936_s_at	MMP9	matrix metallopeptidase 9 (gelatinase B,	1.54	2.91	0.00050668	0.0016287		4318	Hs.297413
331	201732_s_at	CLCN3	chloride channel 3	1.54	2.9	5.42E-09	1.02E-07		1182	Hs.481186
332	203418_at	CCNA2	cyclin A2	1.53	2.89	6.61E-08	7.96E-07		890	Hs.58974
333	204818_at	HSD17B2	hydroxysteroid (17-beta) dehydrogenase	1.53	2.89	0.00030389	0.0010495		3294	Hs.162795
334	209589_s_at	EPHB2	EPH receptor B2	1.53	2.88	1.01E-07	1.13E-06		2048	Hs.523329
335	200727_s_at	ACTR2	ARP2 actin-related protein 2 homolog (y	1.53	2.88	0.00130009	0.003724		10097	Hs.643727, Hs.
336	213872_at	C6orf62	chromosome 6 open reading frame 62	1.53	2.88	0.00014347	0.0005508		81688	Hs.519930
337	206472_s_at	TLE3	transducin-like enhancer of split 3 (E(sp1	1.52	2.88	4.10E-11	1.84E-09		7090	Hs.287362
338	205401_at	AGPS	alkylglycerone phosphate synthase	1.52	2.87	5.74E-06	3.54E-05		8540	Hs.516543
339	213680_at	KRT6B	keratin 6B	1.52	2.87	1.97E-07	2.00E-06		3854	Hs.709235
340	218719_s_at	GINS3	GINS complex subunit 3 (Psf3 homolog)	1.52	2.87	2.21E-12	1.91E-10		64785	Hs.47125, Hs.6
341	204057_at	IRF8	interferon regulatory factor 8	1.52	2.87	5.99E-08	7.36E-07		3394	Hs.137427
342	205419_at	GPR183	G protein-coupled receptor 183	1.52	2.87	6.60E-07	5.64E-06		1880	Hs.784
343	204527_at	MYO5A	myosin VA (heavy chain 12, myoxin)	1.51	2.86	5.87E-08	7.25E-07		4644	Hs.21213, Hs.5
344	207238_s_at	PTPRC	protein tyrosine phosphatase, receptor ty	1.51	2.85	1.22E-05	6.66E-05		5788	Hs.654514
345	205831_at	CD2	CD2 molecule	1.51	2.85	0.00150469	0.0042152		914	Hs.523500
346	201487_at	CTSC	cathepsin C	1.51	2.84	3.39E-15	1.83E-12		1075	Hs.128065, Hs.
347	210020_x_at	CALML3	calmodulin-like 3	1.5	2.83	1.35E-10	4.89E-09		810	Hs.239600
348	204420_at	FOSL1	FOS-like antigen 1	1.5	2.83	0.00056164	0.0017813		8061	Hs.283565
349	204532_x_at			1.5	2.83	1.23E-06	9.55E-06			
350	220800_s_at	TMOD3	tropomodulin 3 (ubiquitous)	1.5	2.82	3.78E-05	0.0001748		29766	Hs.4998
351	204822_at	TTK	TTK protein kinase	1.5	2.82	6.01E-06	3.67E-05		7272	Hs.169840
352	219015_s_at	ALG13	asparagine-linked glycosylation 13 homo	1.49	2.81	9.81E-06	5.50E-05		79868	Hs.443061
353	212659_s_at	IL1RN	interleukin 1 receptor antagonist	1.49	2.81	4.46E-10	1.30E-08		3557	Hs.81134
354	219316_s_at	FLVCR2	feline leukemia virus subgroup C cellular	1.49	2.81	1.82E-10	6.28E-09		55640	Hs.509966, Hs.
355	202705_at	CCNB2	cyclin B2	1.49	2.81	1.02E-05	5.66E-05		9133	Hs.194698
356	203128_at	SPTLC2	serine palmitoyltransferase, long chain b	1.49	2.81	6.88E-11	2.83E-09		9517	Hs.435661
357	213293_s_at	TRIM22	tripartite motif-containing 22	1.49	2.81	1.22E-09	2.95E-08		10346	Hs.501778, Hs.
358	204967_at	SHROOM2	shroom family member 2	1.49	2.81	4.88E-05	0.0002177		357	Hs.567236
359	214829_at	AASS	aminoadipate-semialdehyde synthase	1.49	2.8	8.90E-09	1.54E-07		10157	Hs.156738
360	205543_at	HSPA4L	heat shock 70kDa protein 4-like	1.49	2.8	3.52E-05	0.0001647		22824	Hs.135554
361	221009_s_at	ANGPTL4	angiopoietin-like 4	1.49	2.8	1.63E-07	1.69E-06		51129	Hs.9613
362	202069_s_at	IDH3A	isocitrate dehydrogenase 3 (NAD+) alph	1.49	2.8	1.35E-10	4.89E-09		3419	Hs.591110
363	207463_x_at	PRSS3	protease, serine, 3	1.48	2.8	3.62E-10	1.12E-08		5646	Hs.654513
364	205394_at	CHEK1	CHK1 checkpoint homolog (S. pombe)	1.48	2.79	1.68E-06	1.24E-05		1111	Hs.24529
365	203700_s_at	DIO2	deiodinase, iodothyronine, type II	1.48	2.79	1.14E-10	4.31E-09		1734	Hs.202354
366	216470_x_at	PRSS1	protease, serine, 1 (trypsin 1)	1.48	2.79	2.48E-10	8.21E-09		5644	Hs.622865, Hs.
367	204007_at	FCGR3B	Fc fragment of IgG, low affinity IIIb, rece	1.48	2.79	0.00015438	0.0005881		2215	Hs.694258
368	203974_at	HDHD1A	haloacid dehalogenase-like hydrolase do	1.48	2.78	5.54E-08	6.90E-07		8226	Hs.185910
369	209924_at	CCL18	chemokine (C-C motif) ligand 18 (pulmor	1.48	2.78	0.00250383	0.0065466		6362	Hs.143961
370	209771_x_at	CD24	CD24 molecule	1.47	2.77	2.43E-14	6.95E-12		1E+08	Hs.644105, Hs.
371	206653_at	POLR3G	polymerase (RNA) III (DNA directed) pol	1.47	2.77	4.06E-10	1.22E-08		10622	Hs.282387
372	211195_s_at	TP63	tumor protein p63	1.47	2.76	1.77E-05	9.12E-05		8626	Hs.137569
373	207655_s_at	BLNK	B-cell linker	1.47	2.76	2.07E-10	7.01E-09		29760	Hs.665244
374	218355_at	KIF4A	kinesin family member 4A	1.47	2.76	8.16E-06	4.73E-05		24137	Hs.648326
375	203797_at	VSNL1	visinin-like 1	1.47	2.76	1.11E-11	6.71E-10		7447	Hs.444212
376	214838_at	SFT2D2	SFT2 domain containing 2	1.46	2.75	2.12E-11	1.09E-09		375035	Hs.645435
377	212942_s_at	KIAA1199	KIAA1199	1.46	2.75	4.21E-08	5.54E-07		57214	Hs.459088
378	203821_at	HBEGF	heparin-binding EGF-like growth factor	1.46	2.75	4.44E-06	2.84E-05		1839	Hs.592942, Hs.
379	202067_s_at	LDLR	low density lipoprotein receptor	1.46	2.75	3.77E-05	0.0001745		3949	Hs.213289, Hs.
380	210589_s_at	GBAP	glucosidase, beta; acid, pseudogene	1.46	2.75	4.38E-12	3.13E-10		2630	Hs.282997
381	205159_at	CSF2RB	colony stimulating factor 2 receptor, beta	1.46	2.75	0.00012053	0.0004736		1439	Hs.592192
382	200776_s_at			1.46	2.74	6.07E-09	1.11E-07			
383	201169_s_at	BHLHE40	basic helix-loop-helix family, member e4	1.45	2.74	0.00037829	0.0012614		8553	Hs.719093
384	209464_at	AURKB	aurora kinase B	1.45	2.74	7.55E-07	6.30E-06		9212	Hs.442658
385	214583_at	RSC1A1	regulatory solute carrier protein, family 1	1.45	2.73	5.15E-08	6.54E-07		6248	Hs.145049, Hs.
386	221245_s_at	FZD5	frizzled homolog 5 (Drosophila)	1.45	2.73	4.92E-09	9.32E-08		7855	Hs.17631
387	221803_s_at	NRBF2	nuclear receptor binding factor 2	1.44	2.71	1.64E-09	3.78E-08		29982	Hs.449628
388	202236_s_at	SLC16A1	solute carrier family 16, member 1 (mono	1.44	2.71	2.50E-07	2.43E-06		6566	Hs.75231
389	220066_at	NOD2	nucleotide-binding oligomerization doma	1.44	2.71	1.19E-11	6.89E-10		64127	Hs.592072
390	201101_s_at	BCLAF1	BCL2-associated transcription factor 1	1.44	2.71	1.28E-05	6.95E-05		9774	Hs.486542

	Probe	Symbol	Description	lgFCH	FCH	p	FDR		ENTREZ	UniGene
391	202543_s_at	GMFB	glia maturation factor, beta	1.43	2.7	0.00136873	0.0038826		2764	Hs.151413
392	206488_s_at	CD36	CD36 molecule (thrombospondin recepto	1.43	2.7	3.08E-07	2.91E-06		948	Hs.120949
393	220599_s_at	CARD14	caspase recruitment domain family, mem	1.43	2.7	3.18E-08	4.40E-07		79092	Hs.675480, Hs.
394	218880_at	FOSL2	FOS-like antigen 2	1.43	2.7	5.80E-10	1.62E-08		2355	Hs.220971, Hs.
395	203767_s_at	STS	steroid sulfatase (microsomal), isozyme	1.43	2.69	5.12E-07	4.50E-06		412	Hs.522578, Hs.
396	220745_at	IL19	interleukin 19	1.43	2.69	0.00176373	0.0048264		29949	Hs.661017
397	33304_at	ISG20	interferon stimulated exonuclease gene	1.43	2.69	1.25E-07	1.35E-06		3669	Hs.459265
398	217738_at	NAMPT	nicotinamide phosphoribosyltransferase	1.42	2.68	8.31E-08	9.57E-07		10135	Hs.489615
399	207455_at	P2RY1	purinergic receptor P2Y, G-protein coupl	1.42	2.68	8.03E-07	6.65E-06		5028	Hs.654526
400	205016_at	TGFA	transforming growth factor, alpha	1.42	2.68	5.22E-07	4.57E-06		7039	Hs.170009
401	203362_s_at	MAD2L1	MAD2 mitotic arrest deficient-like 1 (yeas	1.42	2.67	0.00018456	0.0006852		4085	Hs.591697
402	208934_s_at	LGALS8	lectin, galactoside-binding, soluble, 8	1.42	2.67	9.50E-13	9.75E-11		3964	Hs.4082, Hs.70
403	203087_s_at	KIF2A	kinesin heavy chain member 2A	1.42	2.67	8.50E-05	0.000353		3796	Hs.558351
404	208290_s_at	EIF5	eukaryotic translation initiation factor 5	1.41	2.66	5.09E-10	1.44E-08		1983	Hs.433702
405	217094_s_at	ITCH	itchy E3 ubiquitin protein ligase homolog	1.41	2.66	2.37E-10	7.90E-09		83737	Hs.632272
406	202805_s_at	ABCC1	ATP-binding cassette, sub-family C (CFT	1.41	2.66	3.97E-10	1.20E-08		4363	Hs.709181
407	214008_at	TWF1	twinfilin, actin-binding protein, homolog 1	1.41	2.66	0.00056819	0.0017994		5756	Hs.189075
408	202687_s_at	TNFSF10	tumor necrosis factor (ligand) superfamil	1.41	2.65	1.54E-10	5.45E-09		8743	Hs.478275
409	221220_s_at	SCYL2	SCY1-like 2 (S. cerevisiae)	1.41	2.65	1.42E-05	7.55E-05		55681	Hs.506481
410	201733_at	CLCN3	chloride channel 3	1.41	2.65	5.73E-08	7.11E-07		1182	Hs.481186
411	200887_s_at	STAT1	signal transducer and activator of transc	1.41	2.65	9.93E-11	3.88E-09		6772	Hs.642990, Hs.
412	221779_at	MICALL1	MICAL-like 1	1.41	2.65	3.54E-11	1.63E-09		85377	Hs.517610
413	213537_at	HLA-DPA1	major histocompatibility complex, class I	1.4	2.65	0.00078684	0.0023963		3113	Hs.347270
414	208992_s_at	STAT3	signal transducer and activator of transc	1.4	2.65	1.95E-10	6.68E-09		6774	Hs.463059
415	212365_at	MYO1B	myosin IB	1.4	2.64	4.68E-07	4.17E-06		4430	Hs.439620
416	212460_at	C14orf147	chromosome 14 open reading frame 147	1.4	2.64	4.82E-09	9.14E-08		171546	Hs.269909
417	201523_x_at	UBE2N	ubiquitin-conjugating enzyme E2N (UBC	1.4	2.63	1.15E-07	1.27E-06		7334	Hs.524630
418	201123_s_at	EIF5A	eukaryotic translation initiation factor 5A	1.4	2.63	0.02165148	0.0425618		1984	Hs.534314
419	205064_at	SPRR1B	small proline-rich protein 1B (cornifin)	1.4	2.63	1.03E-13	2.00E-11		6699	Hs.1076
420	209520_s_at	NCBP1	nuclear cap binding protein subunit 1, 80	1.4	2.63	1.09E-06	8.60E-06		4686	Hs.595669, Hs.
421	202712_s_at			1.39	2.63	2.10E-11	1.08E-09			
422	208097_s_at	TMX1	thioredoxin-related transmembrane prote	1.39	2.62	0.00028482	0.0009929		81542	Hs.125221
423	222262_s_at	ETNK1	ethanolamine kinase 1	1.39	2.62	2.70E-07	2.60E-06		55500	Hs.29464
424	220608_s_at	ZNF770	zinc finger protein 770	1.39	2.61	0.00804104	0.0180052		54989	Hs.718498
425	203476_at	TPBG	trophoblast glycoprotein	1.38	2.61	4.01E-15	1.91E-12		7162	Hs.82128
426	204994_at	MX2	myxovirus (influenza virus) resistance 2	1.38	2.61	0.0008463	0.002555		4600	Hs.926
427	32069_at	N4BP1	NEDD4 binding protein 1	1.38	2.61	1.79E-12	1.58E-10		9683	Hs.511839
428	214088_s_at	FUT3	fucosyltransferase 3 (galactoside 3(4)-L-	1.38	2.6	2.55E-07	2.47E-06		2525	Hs.169238
429	204103_at	CCL4	chemokine (C-C motif) ligand 4	1.38	2.6	0.00021551	0.0007823		6351	Hs.75703
430	60528_at			1.38	2.6	2.39E-11	1.20E-09			
431	212800_at	STX6	syntaxin 6	1.37	2.59	2.43E-08	3.54E-07		10228	Hs.518417
432	210148_at	HIPK3	homeodomain interacting protein kinase	1.37	2.58	0.00943394	0.0207131		10114	Hs.201918
433	211668_s_at	PLAU	plasminogen activator, urokinase	1.37	2.58	4.95E-06	3.13E-05		5328	Hs.77274
434	202787_s_at	MAPKAPK3	mitogen-activated protein kinase-activate	1.36	2.57	8.37E-12	5.39E-10		7867	Hs.234521
435	216598_s_at	CCL2	chemokine (C-C motif) ligand 2	1.36	2.56	6.96E-06	4.14E-05		6347	Hs.303649
436	206911_at	TRIM25	tripartite motif-containing 25	1.35	2.55	1.18E-07	1.29E-06		7706	Hs.528952
437	202083_s_at	SEC14L1	SEC14-like 1 (S. cerevisiae)	1.35	2.55	1.04E-10	4.02E-09		6397	Hs.464184
438	200841_s_at	EPRS	glutamyl-prolyl-tRNA synthetase	1.35	2.54	2.49E-06	1.74E-05		2058	Hs.497788
439	213548_s_at	CDV3	CDV3 homolog (mouse)	1.35	2.54	0.00018426	0.0006845		55573	Hs.518265
440	219856_at	C1orf116	chromosome 1 open reading frame 116	1.34	2.54	6.36E-08	7.73E-07		79098	Hs.32417
441	219148_at	PBK	PDZ binding kinase	1.34	2.53	0.00025954	0.0009172		55872	Hs.104741
442	214382_at	UNC93A	unc-93 homolog A (C. elegans)	1.33	2.52	2.21E-07	2.19E-06		54346	Hs.567508
443	213421_x_at	PRSS3	protease, serine, 3	1.33	2.51	3.40E-10	1.07E-08		5646	Hs.654513
444	218498_s_at	ERO1L	ERO1-like (S. cerevisiae)	1.32	2.5	1.58E-06	1.18E-05		30001	Hs.592304
445	200648_s_at	GLUL	glutamate-ammonia ligase (glutamine sy	1.32	2.5	4.05E-06	2.63E-05		2752	Hs.518525
446	211622_s_at	ARF3	ADP-ribosylation factor 3	1.32	2.49	3.60E-06	2.36E-05		377	Hs.119177, Hs.
447	211194_s_at	TP63	tumor protein p63	1.32	2.49	0.000774	0.0023639		8626	Hs.137569
448	201649_at	UBE2L6	ubiquitin-conjugating enzyme E2L 6	1.32	2.49	5.36E-09	1.00E-07		9246	Hs.425777
449	212220_at	PSME4	proteasome (prosome, macropain) activa	1.31	2.49	1.08E-09	2.68E-08		23198	Hs.413801
450	217921_at	MAN1A2	mannosidase, alpha, class 1A, member	1.31	2.49	3.36E-05	0.0001584		10905	Hs.435938
451	212246_at	MCFD2	multiple coagulation factor deficiency 2	1.31	2.48	3.73E-10	1.15E-08		90411	Hs.293689
452	213358_at	KIAA0802	KIAA0802	1.31	2.48	2.98E-10	9.54E-09		23255	Hs.650822
453	203233_at	IL4R	interleukin 4 receptor	1.31	2.48	2.41E-11	1.21E-09		3566	Hs.513457
454	209236_at	SLC23A2	solute carrier family 23 (nucleobase tran	1.31	2.48	4.31E-07	3.89E-06		9962	Hs.516866
455	205832_at	CPA4	carboxypeptidase A4	1.31	2.48	7.20E-10	1.92E-08		51200	Hs.93764

	Probe	Symbol	Description	lgFCH	FCH	p	FDR		ENTREZ	UniGene
456	201735_s_at	CLCN3	chloride channel 3	1.31	2.48	6.69E-13	7.30E-11		1182	Hs.481186
457	217202_s_at	GLUL	glutamate-ammonia ligase (glutamine sy	1.31	2.47	3.52E-06	2.32E-05		2752	Hs.518525
458	213131_at	OLFM1	olfactomedin 1	1.31	2.47	1.05E-08	1.77E-07		10439	Hs.522484
459	209408_at	KIF2C	kinesin family member 2C	1.3	2.47	1.13E-06	8.91E-06		11004	Hs.69360
460	207620_s_at	CASK	calcium/calmodulin-dependent serine pro	1.3	2.47	5.67E-07	4.92E-06		8573	Hs.495984
461	219680_at	NLRX1	NLR family member X1	1.3	2.46	6.75E-11	2.80E-09		79671	Hs.524082
462	216125_s_at	RANBP9	RAN binding protein 9	1.3	2.46	3.49E-08	4.76E-07		10048	Hs.708182
463	218663_at	NCAPG	non-SMC condensin I complex, subunit G	1.3	2.46	6.13E-05	0.0002648		64151	Hs.567567
464	207339_s_at	LTB	lymphotoxin beta (TNF superfamily, mem	1.3	2.46	1.80E-05	9.28E-05		4050	Hs.376208
465	205034_at	CCNE2	cyclin E2	1.29	2.45	0.00016211	0.0006132		9134	Hs.567387
466	202430_s_at	PLSCR1	phospholipid scramblase 1	1.29	2.45	9.61E-07	7.73E-06		5359	Hs.130759
467	208744_x_at	HSPH1	heat shock 105kDa/110kDa protein 1	1.29	2.45	1.02E-05	5.69E-05		10808	Hs.36927
468	220104_at	ZC3HAV1	zinc finger CCCH-type, antiviral 1	1.29	2.44	1.63E-08	2.53E-07		56829	Hs.133512
469	218120_s_at	HMOX2	heme oxygenase (decycling) 2	1.29	2.44	2.13E-13	3.15E-11		3163	Hs.284279
470	206877_at	MXD1	MAX dimerization protein 1	1.29	2.44	7.68E-06	4.49E-05		4084	Hs.468908
471	206364_at	KIF14	kinesin family member 14	1.29	2.44	0.00010822	0.0004317		9928	Hs.3104
472	219544_at	C13orf34	chromosome 13 open reading frame 34	1.29	2.44	1.75E-07	1.80E-06		79866	Hs.714340
473	203964_at	NMI	N-myc (and STAT) interactor	1.29	2.44	2.35E-12	1.96E-10		9111	Hs.54483
474	202064_s_at	SEL1L	sel-1 suppressor of lin-12-like (C. elegan	1.29	2.44	1.31E-05	7.07E-05		6400	Hs.181300
475	218986_s_at	DDX60	DEAD (Asp-Glu-Ala-Asp) box polypeptid	1.29	2.44	7.46E-06	4.39E-05		55601	Hs.591710
476	211113_s_at	ABCG1	ATP-binding cassette, sub-family G (WH	1.28	2.43	7.59E-09	1.34E-07		9619	Hs.124649
477	215395_x_at			1.28	2.43	1.66E-09	3.81E-08			
478	208708_x_at	EIF5	eukaryotic translation initiation factor 5	1.28	2.43	1.26E-13	2.11E-11		1983	Hs.433702
479	205046_at	CENPE	centromere protein E, 312kDa	1.28	2.43	9.43E-06	5.33E-05		1062	Hs.75573
480	222039_at	KIF18B	kinesin family member 18B	1.28	2.43	9.16E-07	7.43E-06		146909	Hs.135094
481	217496_s_at	IDE	insulin-degrading enzyme	1.28	2.43	1.05E-08	1.78E-07		3416	Hs.500546
482	203213_at	CDC2	cell division cycle 2, G1 to S and G2 to M	1.28	2.43	5.90E-05	0.0002563		983	Hs.334562
483	204962_s_at	CENPA	centromere protein A	1.28	2.43	0.00016974	0.0006381		1058	Hs.1594
484	201469_s_at	SHC1	SHC (Src homology 2 domain containing	1.28	2.42	1.21E-05	6.65E-05		6464	Hs.433795
485	202479_s_at	TRIB2	tribbles homolog 2 (Drosophila)	1.28	2.42	2.22E-07	2.20E-06		28951	Hs.467751
486	221416_at	PLA2G2F	phospholipase A2, group IIF	1.28	2.42	0.00030481	0.0010521		64600	Hs.302034
487	209761_s_at	SP110	SP110 nuclear body protein	1.28	2.42	3.94E-08	5.26E-07		3431	Hs.145150
488	220620_at	CRCT1	cysteine-rich C-terminal 1	1.27	2.42	8.44E-11	3.36E-09		54544	Hs.110196
489	204268_at	S100A2	S100 calcium binding protein A2	1.27	2.42	2.60E-10	8.52E-09		6273	Hs.516484
490	208937_s_at	ID1	inhibitor of DNA binding 1, dominant neg	1.27	2.42	6.36E-08	7.73E-07		3397	Hs.504609
491	201739_at	SGK1	serum/glucocorticoid regulated kinase 1	1.27	2.41	7.58E-13	8.19E-11		6446	Hs.510078
492	205287_s_at	TFAP2C	transcription factor AP-2 gamma (activat	1.27	2.41	0.00017972	0.0006705		7022	Hs.473152
493	207254_at	SLC15A1	solute carrier family 15 (oligopeptide tran	1.27	2.41	2.95E-10	9.53E-09		6564	Hs.436893
494	218951_s_at	PLCXD1	phosphatidylinositol-specific phospholipa	1.27	2.41	1.96E-07	1.99E-06		55344	Hs.522568
495	48030_i_at	C5orf4	chromosome 5 open reading frame 4	1.27	2.41	0.00019493	0.0007177		10826	Hs.519694, Hs.
496	202625_at	LYN	v-yes-1 Yamaguchi sarcoma viral related	1.27	2.41	2.99E-10	9.56E-09		4067	Hs.699154
497	209825_s_at	UCK2	uridine-cytidine kinase 2	1.26	2.4	1.58E-06	1.18E-05		7371	Hs.458360
498	207165_at	HMMR	hyaluronan-mediated motility receptor (R	1.26	2.4	0.00063949	0.0020001		3161	Hs.72550
499	204478_s_at	RABIF	RAB interacting factor	1.26	2.39	1.33E-10	4.89E-09		5877	Hs.90875
500	208309_s_at	MALT1	mucosa associated lymphoid tissue lymp	1.26	2.39	1.19E-05	6.53E-05		10892	Hs.601217
501	212107_s_at	DHX9	DEAH (Asp-Glu-Ala-His) box polypeptide	1.26	2.39	0.00014987	0.0005727		1660	Hs.191518
502	214155_s_at	LARP4	La ribonucleoprotein domain family, mem	1.26	2.39	5.73E-06	3.53E-05		113251	Hs.26613
503	205194_at	PSPH	phosphoserine phosphatase	1.26	2.39	1.83E-06	1.33E-05		5723	Hs.512656
504	212290_at	SLC7A1	solute carrier family 7 (cationic amino ac	1.25	2.38	5.29E-05	0.0002336		6541	Hs.14846
505	219934_s_at	SULT1E1	sulfotransferase family 1E, estrogen-pre	1.25	2.37	0.00235522	0.0061971		6783	Hs.479898
506	214599_at	IVL	involucrin	1.25	2.37	3.65E-12	2.73E-10		3713	Hs.516439
507	207291_at	PRRG4	proline rich Gla (G-carboxyglutamic acid	1.25	2.37	1.09E-08	1.82E-07		79056	Hs.471695
508	210458_s_at	TANK	TRAF family member-associated NFKB	1.24	2.37	1.04E-06	8.33E-06		10010	Hs.132257
509	206632_s_at	APOBEC3B	apolipoprotein B mRNA editing enzyme,	1.24	2.36	9.42E-05	0.0003833		9582	Hs.226307
510	205774_at	F12	coagulation factor XII (Hageman factor)	1.24	2.36	1.27E-06	9.82E-06		2161	Hs.1321
511	211806_s_at	KCNJ15	potassium inwardly-rectifying channel, su	1.24	2.36	8.91E-10	2.28E-08		3772	Hs.411299
512	219412_at	RAB38	RAB38, member RAS oncogene family	1.24	2.35	2.65E-11	1.29E-09		23682	Hs.591975
513	203328_x_at	IDE	insulin-degrading enzyme	1.23	2.35	2.63E-09	5.59E-08		3416	Hs.500546
514	206562_s_at	CSNK1A1	casein kinase 1, alpha 1	1.23	2.35	5.45E-07	4.76E-06		1452	Hs.529862, Hs.
515	204891_s_at	LCK	lymphocyte-specific protein tyrosine kina	1.23	2.35	0.00822796	0.0183949		3932	Hs.470627
516	219017_at	ETNK1	ethanolamine kinase 1	1.23	2.35	1.27E-05	6.89E-05		55500	Hs.29464
517	209853_s_at	PSME3	proteasome (prosome, macropain) activa	1.23	2.34	1.56E-10	5.49E-09		10197	Hs.152978
518	205479_s_at	PLAU	plasminogen activator, urokinase	1.23	2.34	3.52E-06	2.32E-05		5328	Hs.77274
519	221539_at	EIF4EBP1	eukaryotic translation initiation factor 4E	1.23	2.34	1.13E-09	2.77E-08		1978	Hs.411641
520	206683_at	ZNF165	zinc finger protein 165	1.23	2.34	9.24E-07	7.49E-06		7718	Hs.535177

	Probe	Symbol	Description	lgFCH	FCH	p	FDR		ENTREZ	UniGene
521	218209_s_at	RPRD1A	regulation of nuclear pre-mRNA domain	1.23	2.34	7.65E-05	0.0003215		55197	Hs.464912
522	203573_s_at	RABGGTA	Rab geranylgeranyltransferase, alpha su	1.23	2.34	5.70E-09	1.06E-07		5875	Hs.377992
523	218562_s_at	TMEM57	transmembrane protein 57	1.23	2.34	3.02E-11	1.45E-09		55219	Hs.189782
524	202095_s_at	BIRC5	baculoviral IAP repeat-containing 5	1.23	2.34	3.89E-06	2.53E-05		332	Hs.514527
525	213857_s_at	CD47	CD47 molecule	1.22	2.34	1.23E-16	1.54E-13		961	Hs.446414
526	219211_at	USP18	ubiquitin specific peptidase 18	1.22	2.34	0.00067655	0.0021007		11274	Hs.38260
527	218073_s_at	TMEM48	transmembrane protein 48	1.22	2.33	0.00013796	0.0005325		55706	Hs.476525
528	203471_s_at	PLEK	pleckstrin	1.22	2.33	0.00033179	0.00113		5341	Hs.468840
529	207414_s_at	PCSK6	proprotein convertase subtilisin/kexin typ	1.22	2.33	5.90E-08	7.27E-07		5046	Hs.498494, Hs.
530	205008_s_at	CIB2	calcium and integrin binding family mem	1.22	2.33	4.65E-06	2.96E-05		10518	Hs.129867
531	209744_x_at	ITCH	itchy E3 ubiquitin protein ligase homolog	1.22	2.32	8.43E-09	1.46E-07		83737	Hs.632272
532	209025_s_at	SYNCRIP	synaptotagmin binding, cytoplasmic RNA	1.22	2.32	1.57E-05	8.25E-05		10492	Hs.571177
533	212142_at	MCM4	minichromosome maintenance complex	1.21	2.32	1.62E-05	8.44E-05		4173	Hs.460184
534	209925_at	OCLN	occludin	1.21	2.31	8.20E-06	4.75E-05		4950	Hs.592605
535	213875_x_at	C6orf62	chromosome 6 open reading frame 62	1.21	2.31	0.00393013	0.0096853		81688	Hs.519930
536	202070_s_at	IDH3A	isocitrate dehydrogenase 3 (NAD+) alph	1.21	2.31	1.48E-08	2.34E-07		3419	Hs.591110
537	201435_s_at	EIF4E	eukaryotic translation initiation factor 4E	1.21	2.31	2.02E-05	0.0001023		1977	Hs.249718
538	207017_at	RAB27B	RAB27B, member RAS oncogene family	1.21	2.31	7.84E-08	9.14E-07		5874	Hs.25318
539	205349_at	GNA15	guanine nucleotide binding protein (G pr	1.21	2.31	1.55E-09	3.58E-08		2769	Hs.73797
540	209949_at	NCF2	neutrophil cytosolic factor 2	1.2	2.3	2.60E-06	1.80E-05		4688	Hs.587558
541	203560_at	GGH	gamma-glutamyl hydrolase (conjugase,	1.2	2.3	3.05E-09	6.27E-08		8836	Hs.78619
542	221521_s_at	GINS2	GINS complex subunit 2 (Psf2 homolog)	1.2	2.3	0.00027172	0.0009519		51659	Hs.433180
543	216399_s_at	SCAPER	S-phase cyclin A-associated protein in th	1.2	2.3	0.0001995	0.0007318		49855	Hs.458986
544	220941_s_at	C21orf91	chromosome 21 open reading frame 91	1.2	2.3	2.58E-05	0.0001265		54149	Hs.293811
545	219187_at	FKBPL	FK506 binding protein like	1.2	2.3	3.72E-10	1.15E-08		63943	Hs.520042
546	205015_s_at	TGFA	transforming growth factor, alpha	1.2	2.3	6.41E-05	0.0002752		7039	Hs.170009
547	212479_s_at	RMND5A	required for meiotic nuclear division 5 ho	1.2	2.29	6.72E-11	2.80E-09		64795	Hs.75277
548	213927_at	MAP3K9	mitogen-activated protein kinase kinase	1.19	2.29	2.98E-06	2.02E-05		4293	Hs.445496, Hs.
549	55081_at	MICALL1	MICAL-like 1	1.19	2.29	2.05E-09	4.53E-08		85377	Hs.517610
550	217764_s_at	RAB31	RAB31, member RAS oncogene family	1.19	2.28	4.21E-13	5.31E-11		11031	Hs.714730, Hs.
551	216607_s_at	CYP51A1	cytochrome P450, family 51, subfamily A	1.19	2.28	2.30E-05	0.0001144		1595	Hs.417077
552	219428_s_at	PXMP4	peroxisomal membrane protein 4, 24kDa	1.19	2.28	3.25E-06	2.17E-05		11264	Hs.654857
553	203770_s_at	STS	steroid sulfatase (microsomal), isozyme	1.19	2.28	3.21E-06	2.15E-05		412	Hs.522578, Hs.
554	219657_s_at	KLF3	Kruppel-like factor 3 (basic)	1.18	2.27	2.19E-10	7.39E-09		51274	Hs.298658
555	212514_x_at	DDX3X	DEAD (Asp-Glu-Ala-Asp) box polypeptid	1.18	2.27	0.00040281	0.0013314		1654	Hs.719127
556	202058_s_at	KPNA1	karyopherin alpha 1 (importin alpha 5)	1.18	2.27	3.23E-06	2.16E-05		3836	Hs.161008
557	211924_s_at	PLAUR	plasminogen activator, urokinase recepto	1.18	2.27	0.00013101	0.0005099		5329	Hs.466871
558	220013_at	EPHX3	epoxide hydrolase 3	1.18	2.26	1.48E-11	8.15E-10		79852	Hs.156457
559	221059_s_at			1.18	2.26	2.38E-07	2.32E-06			
560	202338_at	TK1	thymidine kinase 1, soluble	1.18	2.26	3.23E-06	2.16E-05		7083	Hs.515122
561	210052_s_at	TPX2	TPX2, microtubule-associated, homolog	1.18	2.26	2.30E-06	1.63E-05		22974	Hs.708960, Hs.
562	202595_s_at	LEPROTL1	leptin receptor overlapping transcript-like	1.18	2.26	1.14E-11	6.79E-10		23484	Hs.146585
563	209976_s_at	CYP2E1	cytochrome P450, family 2, subfamily E,	1.17	2.26	0.00033675	0.0011436		1571	Hs.12907
564	203595_s_at	IFIT5	interferon-induced protein with tetratricop	1.17	2.26	6.64E-05	0.0002839		24138	Hs.252839
565	219267_at	GLTP	glycolipid transfer protein	1.17	2.25	1.75E-09	4.01E-08		51228	Hs.381256
566	217919_s_at	MRPL42	mitochondrial ribosomal protein L42	1.17	2.25	1.66E-06	1.23E-05		28977	Hs.199579
567	60474_at	FERMT1	fermitin family homolog 1 (Drosophila)	1.17	2.25	1.17E-07	1.29E-06		55612	Hs.472054
568	214869_x_at	GAPVD1	GTPase activating protein and VPS9 do	1.17	2.25	0.00043263	0.001419		26130	Hs.495134
569	218748_s_at	EXOC5	exocyst complex component 5	1.17	2.25	0.01057272	0.0228738		10640	Hs.715522
570	203416_at	CD53	CD53 molecule	1.17	2.25	1.22E-05	6.66E-05		963	Hs.443057
571	204826_at	CCNF	cyclin F	1.17	2.24	1.03E-06	8.26E-06		899	Hs.1973
572	203755_at	BUB1B	budding uninhibited by benzimidazoles 1	1.17	2.24	0.00069907	0.0021628		701	Hs.631699
573	218095_s_at	TMEM165	transmembrane protein 165	1.16	2.24	8.74E-12	5.54E-10		55858	Hs.479766
574	213603_s_at	RAC2	ras-related C3 botulinum toxin substrate	1.16	2.24	2.77E-05	0.0001345		5880	Hs.517601
575	216905_s_at	ST14	suppression of tumorigenicity 14 (colon c	1.16	2.23	3.95E-09	7.69E-08		6768	Hs.504315
576	205269_at	LCP2	lymphocyte cytosolic protein 2 (SH2 dom	1.16	2.23	0.000772	0.0023589		3937	Hs.304475
577	212527_at	PPPDE2	PPPDE peptidase domain containing 2	1.16	2.23	1.75E-06	1.28E-05		27351	Hs.570455
578	209872_s_at	PKP3	plakophilin 3	1.16	2.23	0.00052605	0.0016813		11187	Hs.534395
579	208952_s_at	LARP4B	La ribonucleoprotein domain family, mer	1.16	2.23	5.78E-10	1.62E-08		23185	Hs.681734, Hs.
580	213599_at	OIP5	Opa interacting protein 5	1.15	2.22	8.84E-06	5.06E-05		11339	Hs.661645
581	220285_at	FAM108B1	family with sequence similarity 108, mer	1.15	2.22	7.93E-08	9.23E-07		51104	Hs.380389
582	202671_s_at	PDXK	pyridoxal (pyridoxine, vitamin B6) kinase	1.15	2.22	3.65E-06	2.39E-05		8566	Hs.284491
583	200987_x_at	PSME3	proteasome (prosome, macropain) activa	1.15	2.22	4.95E-08	6.35E-07		10197	Hs.152978
584	202199_s_at	SRPK1	SFRS protein kinase 1	1.15	2.22	3.20E-06	2.15E-05		6732	Hs.443861
585	205534_at	PCDH7	protocadherin 7	1.15	2.22	9.02E-05	0.0003696		5099	Hs.479439

	Probe	Symbol	Description	lgFCH	FCH	p	FDR		ENTREZ	UniGene
586	200889_s_at	SSR1	signal sequence receptor, alpha	1.15	2.22	2.82E-09	5.91E-08		6745	Hs.114033
587	218327_s_at	SNAP29	synaptosomal-associated protein, 29kDa	1.15	2.22	7.02E-10	1.90E-08		9342	Hs.108002, Hs.
588	200798_x_at	MCL1	myeloid cell leukemia sequence 1 (BCL2	1.15	2.22	0.00041505	0.001368		4170	Hs.719112
589	201291_s_at	TOP2A	topoisomerase (DNA) II alpha 170kDa	1.15	2.22	0.00252516	0.006589		7153	Hs.156346
590	219662_at	C2orf49	chromosome 2 open reading frame 49	1.15	2.21	1.91E-08	2.88E-07		79074	Hs.549577
591	205809_s_at	WASL	Wiskott-Aldrich syndrome-like	1.14	2.21	0.00596718	0.0139011		8976	Hs.143728, Hs.
592	207126_x_at			1.14	2.21	4.31E-06	2.77E-05			
593	213577_at	SQLE	squalene epoxidase	1.14	2.21	0.0002828	0.0009872		6713	Hs.71465
594	205282_at	LRP8	low density lipoprotein receptor-related p	1.14	2.21	6.73E-07	5.72E-06		7804	Hs.719248
595	210935_s_at	WDR1	WD repeat domain 1	1.14	2.21	7.53E-06	4.42E-05		9948	Hs.128548, Hs.
596	219296_at	ZDHHC13	zinc finger, DHHC-type containing 13	1.14	2.21	6.60E-10	1.82E-08		54503	Hs.188569
597	209679_s_at	SMAGP	small trans-membrane and glycosylated	1.14	2.2	1.14E-08	1.88E-07		57228	Hs.652389
598	221477_s_at	SOD2	superoxide dismutase 2, mitochondrial	1.14	2.2	4.63E-06	2.94E-05		6648	Hs.487046
599	203554_x_at	PTTG1	pituitary tumor-transforming 1	1.14	2.2	2.31E-06	1.63E-05		9232	Hs.350966
600	203276_at	LMNB1	lamin B1	1.14	2.2	8.69E-05	0.0003588		4001	Hs.89497
601	211150_s_at	DLAT	dihydrolipoamide S-acetyltransferase	1.14	2.2	4.33E-05	0.0001967		1737	Hs.335551
602	212374_at	FEM1B	fem-1 homolog b (C. elegans)	1.14	2.2	7.51E-06	4.41E-05		10116	Hs.362733
603	202068_s_at	LDLR	low density lipoprotein receptor	1.14	2.2	3.61E-06	2.37E-05		3949	Hs.213289, Hs.
604	206164_at	CLCA2	chloride channel accessory 2	1.14	2.2	1.45E-10	5.18E-09		9635	Hs.241551
605	217762_s_at	RAB31	RAB31, member RAS oncogene family	1.14	2.2	7.29E-10	1.94E-08		11031	Hs.714730, Hs.
606	217741_s_at	ZFAND5	zinc finger, AN1-type domain 5	1.14	2.2	9.01E-09	1.55E-07		7763	Hs.406096
607	207992_s_at	AMPD3	adenosine monophosphate deaminase (	1.14	2.2	3.59E-09	7.16E-08		272	Hs.501890
608	204033_at	TRIP13	thyroid hormone receptor interactor 13	1.13	2.2	0.00034133	0.0011558		9319	Hs.436187
609	209159_s_at	NDRG4	NDRG family member 4	1.13	2.19	0.00011072	0.0004401		65009	Hs.322430
610	210594_x_at	MPZL1	myelin protein zero-like 1	1.13	2.19	1.31E-07	1.41E-06		9019	Hs.493919, Hs.
611	219918_s_at	ASPM	asp (abnormal spindle) homolog, microc	1.13	2.19	0.00406273	0.0099719		259266	Hs.121028
612	206765_at	KCNJ2	potassium inwardly-rectifying channel, s	1.13	2.19	9.69E-05	0.0003926		3759	Hs.1547
613	206094_x_at			1.13	2.19	6.69E-06	4.01E-05			
614	208116_s_at	MAN1A1	mannosidase, alpha, class 1A, member	1.13	2.18	0.01079148	0.0232649		4121	Hs.102788
615	212587_s_at	PTPRC	protein tyrosine phosphatase, receptor ty	1.13	2.18	0.02139525	0.0421354		5788	Hs.654514
616	209457_at	DUSP5	dual specificity phosphatase 5	1.13	2.18	0.0017629	0.0048251		1847	Hs.2128
617	202531_at	IRF1	interferon regulatory factor 1	1.13	2.18	5.27E-08	6.65E-07		3659	Hs.436061
618	211549_s_at	HPGD	hydroxyprostaglandin dehydrogenase 15	1.12	2.18	0.00012066	0.0004739		3248	Hs.596913
619	202345_s_at	FABP5	fatty acid binding protein 5 (psoriasis-ass	1.12	2.18	9.45E-16	7.15E-13		2171	Hs.408061
620	211559_s_at	CCNG2	cyclin G2	1.12	2.18	0.0136355	0.0285261		901	Hs.13291
621	201543_s_at	SAR1A	SAR1 homolog A (S. cerevisiae)	1.12	2.18	1.57E-07	1.65E-06		56681	Hs.499960, Hs.
622	221843_s_at	KIAA1609	KIAA1609	1.12	2.18	5.43E-06	3.37E-05		57707	Hs.288274
623	209237_s_at	SLC23A2	solute carrier family 23 (nucleobase tran	1.12	2.17	0.0028101	0.0072284		9962	Hs.516866
624	45633_at	GINS3	GINS complex subunit 3 (Psf3 homolog)	1.12	2.17	1.91E-11	9.93E-10		64785	Hs.47125, Hs.6
625	202444_s_at	ERLIN1	ER lipid raft associated 1	1.12	2.17	5.38E-06	3.35E-05		10613	Hs.150087
626	209417_s_at	IFI35	interferon-induced protein 35	1.12	2.17	0.00152464	0.0042618		3430	Hs.632258
627	205767_at	EREG	epiregulin	1.12	2.17	5.01E-05	0.0002221		2069	Hs.115263
628	220780_at	PLA2G3	phospholipase A2, group III	1.12	2.17	1.31E-05	7.09E-05		50487	Hs.149623
629	200923_at	LGALS3BP	lectin, galactoside-binding, soluble, 3 bir	1.12	2.17	3.02E-07	2.87E-06		3959	Hs.514535
630	212379_at	GART	phosphoribosylglycinamide formyltransfe	1.11	2.17	2.39E-07	2.33E-06		2618	Hs.473648
631	213193_x_at			1.11	2.17	0.00126576	0.0036394			
632	206109_at	FUT1	fucosyltransferase 1 (galactoside 2-alpha	1.11	2.17	1.76E-05	9.12E-05		2523	Hs.69747
633	202497_x_at	SLC2A3	solute carrier family 2 (facilitated glucose	1.11	2.16	6.55E-05	0.0002808		6515	Hs.419240
634	201912_s_at	GSPT1	G1 to S phase transition 1	1.11	2.16	2.59E-08	3.71E-07		2935	Hs.528780
635	208012_x_at	SP110	SP110 nuclear body protein	1.11	2.16	1.39E-06	1.06E-05		3431	Hs.145150
636	218890_x_at	MRPL35	mitochondrial ribosomal protein L35	1.11	2.15	0.00014126	0.0005436		51318	Hs.433439
637	214499_s_at	BCLAF1	BCL2-associated transcription factor 1	1.11	2.15	1.17E-05	6.44E-05		9774	Hs.486542
638	203021_at	SLPI	secretory leukocyte peptidase inhibitor	1.1	2.15	7.49E-07	6.26E-06		6590	Hs.517070
639	212009_s_at	STIP1	stress-induced-phosphoprotein 1	1.1	2.15	4.43E-05	0.0002004		10963	Hs.337295
640	212978_at	LRRC8B	leucine rich repeat containing 8 family, m	1.1	2.15	5.18E-06	3.25E-05		23507	Hs.482017, Hs.
641	220603_s_at	MCTP2	multiple C2 domains, transmembrane 2	1.1	2.14	4.86E-06	3.08E-05		55784	Hs.33368, Hs.5
642	210048_at	NAPG	N-ethylmaleimide-sensitive factor attach	1.1	2.14	8.84E-08	1.01E-06		8774	Hs.464622
643	205778_at	KLK7	kallikrein-related peptidase 7	1.1	2.14	2.58E-09	5.50E-08		5650	Hs.151254
644	209142_s_at	UBE2G1	ubiquitin-conjugating enzyme E2G 1 (UE	1.1	2.14	3.83E-09	7.51E-08		7326	Hs.714345
645	206125_s_at	KLK8	kallikrein-related peptidase 8	1.09	2.13	1.36E-09	3.22E-08		11202	Hs.104570
646	207540_s_at	SYK	spleen tyrosine kinase	1.09	2.13	1.08E-07	1.19E-06		6850	Hs.371720
647	217448_s_at	TOX4	TOX high mobility group box family mem	1.09	2.13	1.90E-09	4.25E-08		9878	Hs.555910
648	209762_x_at	SP110	SP110 nuclear body protein	1.09	2.13	1.26E-05	6.87E-05		3431	Hs.145150
649	217043_s_at	MFN1	mitofusin 1	1.09	2.13	1.13E-10	4.31E-09		55669	Hs.478383
650	200628_s_at	WARS	tryptophanyl-tRNA synthetase	1.09	2.13	5.77E-06	3.55E-05		7453	Hs.497599

	Probe	Symbol	Description	lgFCH	FCH	p	FDR		ENTREZ	UniGene
651	214512_s_at	SUB1	SUB1 homolog (S. cerevisiae)	1.09	2.13	1.87E-10	6.41E-09		10923	Hs.229641, Hs.
652	204092_s_at	AURKA	aurora kinase A	1.09	2.13	1.90E-08	2.88E-07		6790	Hs.250822
653	218662_s_at	NCAPG	non-SMC condensin I complex, subunit	1.09	2.13	6.86E-06	4.09E-05		64151	Hs.567567
654	219399_at	LIN7C	lin-7 homolog C (C. elegans)	1.09	2.13	0.00029619	0.0010265		55327	Hs.693840, Hs.
655	212588_at	PTPRC	protein tyrosine phosphatase, receptor ty	1.09	2.13	0.0014886	0.0041722		5788	Hs.654514
656	200838_at	CTSB	cathepsin B	1.09	2.13	8.12E-10	2.11E-08		1508	Hs.520898
657	211367_s_at	CASP1	caspase 1, apoptosis-related cysteine pe	1.09	2.12	1.35E-07	1.44E-06		834	Hs.2490
658	214698_at	ROD1	ROD1 regulator of differentiation 1 (S. po	1.08	2.12	3.97E-10	1.20E-08		9991	Hs.269988
659	201298_s_at	MOBKL1B	MOB1, Mps One Binder kinase activator	1.08	2.12	1.83E-05	9.40E-05		55233	Hs.196437
660	220655_at	TNIP3	TNFAIP3 interacting protein 3	1.08	2.11	0.01167324	0.0249236		79931	Hs.208206
661	206332_s_at	IFI16	interferon, gamma-inducible protein 16	1.08	2.11	2.89E-12	2.29E-10		3428	Hs.380250
662	217835_x_at	C20orf24	chromosome 20 open reading frame 24	1.08	2.11	6.54E-12	4.33E-10		55969	Hs.584985
663	202954_at	UBE2C	ubiquitin-conjugating enzyme E2C	1.08	2.11	1.36E-05	7.28E-05		11065	Hs.93002
664	218796_at	FERMT1	fermitin family homolog 1 (Drosophila)	1.08	2.11	4.63E-07	4.13E-06		55612	Hs.472054
665	209954_x_at	SS18	synovial sarcoma translocation, chromos	1.08	2.11	3.60E-10	1.12E-08		6760	Hs.404263
666	222309_at	C6orf62	chromosome 6 open reading frame 62	1.08	2.11	0.00176234	0.0048246		81688	Hs.519930
667	204769_s_at	TAP2	transporter 2, ATP-binding cassette, sub	1.08	2.11	5.82E-07	5.05E-06		6891	Hs.502
668	201274_at	PSMA5	proteasome (prosome, macropain) subu	1.08	2.11	7.80E-09	1.37E-07		5686	Hs.712557
669	205402_x_at	PRSS2	protease, serine, 2 (trypsin 2)	1.07	2.1	3.65E-09	7.27E-08		5645	Hs.622865
670	206912_at	FOXE1	forkhead box E1 (thyroid transcription fa	1.07	2.1	1.53E-05	8.08E-05		2304	Hs.159234
671	204881_s_at	UGCG	UDP-glucose ceramide glucosyltransfera	1.07	2.1	3.12E-06	2.10E-05		7357	Hs.304249, Hs.
672	201663_s_at	SMC4	structural maintenance of chromosomes	1.07	2.1	0.00015469	0.0005889		10051	Hs.58992
673	221903_s_at	CYLD	cylindromatosis (turban tumor syndrome	1.07	2.1	4.94E-10	1.41E-08		1540	Hs.578973
674	203293_s_at	LMAN1	lectin, mannose-binding, 1	1.07	2.1	0.00025792	0.000912		3998	Hs.465295
675	212657_s_at	IL1RN	interleukin 1 receptor antagonist	1.07	2.1	5.96E-09	1.10E-07		3557	Hs.81134
676	200734_s_at	ARF3	ADP-ribosylation factor 3	1.07	2.1	1.59E-08	2.49E-07		377	Hs.119177, Hs.
677	219272_at	TRIM62	tripartite motif-containing 62	1.07	2.1	2.10E-08	3.12E-07		55223	Hs.656006
678	211762_s_at	KPNA2	karyopherin alpha 2 (RAG cohort 1, impo	1.07	2.09	1.01E-08	1.72E-07		3838	Hs.594238
679	201201_at	CSTB	cystatin B (stefin B)	1.07	2.09	3.85E-10	1.18E-08		1476	Hs.695
680	204715_at	PANX1	pannexin 1	1.06	2.09	8.74E-05	0.00036		24145	Hs.591976
681	206114_at	EPHA4	EPH receptor A4	1.06	2.09	0.00142504	0.0040201		2043	Hs.371218
682	206075_s_at	CSNK2A1	casein kinase 2, alpha 1 polypeptide	1.06	2.09	4.53E-05	0.0002041		1457	Hs.644056
683	214895_s_at	ADAM10	ADAM metallopeptidase domain 10	1.06	2.09	5.03E-05	0.0002233		102	Hs.578508
684	213007_at	FANCI	Fanconi anemia, complementation group	1.06	2.08	0.00012033	0.0004731		55215	Hs.513126
685	209891_at	SPC25	SPC25, NDC80 kinetochore complex co	1.06	2.08	1.38E-05	7.39E-05		57405	Hs.421956
686	205270_s_at	LCP2	lymphocyte cytosolic protein 2 (SH2 dom	1.06	2.08	2.14E-05	0.0001075		3937	Hs.304475
687	206036_s_at	REL	v-rel reticuloendotheliosis viral oncogene	1.06	2.08	1.85E-06	1.34E-05		5966	Hs.631886
688	204059_s_at	ME1	malic enzyme 1, NADP(+)-dependent, cy	1.05	2.07	2.57E-06	1.78E-05		4199	Hs.21160
689	211715_s_at	BDH1	3-hydroxybutyrate dehydrogenase, type	1.05	2.07	6.43E-08	7.80E-07		622	Hs.274539
690	206421_s_at	SERPINB7	serpin peptidase inhibitor, clade B (ovalb	1.05	2.07	6.00E-06	3.67E-05		8710	Hs.138202
691	204444_at	KIF11	kinesin family member 11	1.05	2.07	0.00154613	0.0043133		3832	Hs.8878
692	200641_s_at	YWHAZ	tyrosine 3-monooxygenase/tryptophan 5	1.05	2.07	0.00460949	0.0111125		7534	Hs.492407, Hs.
693	216915_s_at	PTPN12	protein tyrosine phosphatase, non-recep	1.05	2.07	0.00070035	0.0021657		5782	Hs.61812
694	212807_s_at	SORT1	sortilin 1	1.05	2.07	1.93E-08	2.90E-07		6272	Hs.485195, Hs.
695	214960_at	API5	apoptosis inhibitor 5	1.05	2.07	4.20E-06	2.71E-05		8539	Hs.435771
696	201992_s_at	KIF5B	kinesin family member 5B	1.05	2.07	0.00176919	0.0048372		3799	Hs.644646
697	212432_at	GRPEL1	GrpE-like 1, mitochondrial (E. coli)	1.05	2.07	3.64E-06	2.38E-05		80273	Hs.443723
698	200815_s_at	PAFAH1B1	platelet-activating factor acetylhydrolase	1.05	2.07	7.42E-11	3.01E-09		5048	Hs.77318
699	213933_at	PTGER3	prostaglandin E receptor 3 (subtype EP3	1.05	2.07	0.00018002	0.0006714		5733	Hs.445000
700	205014_at	FGFBP1	fibroblast growth factor binding protein 1	1.05	2.06	0.00061742	0.0019372		9982	Hs.1690
701	208966_x_at	IFI16	interferon, gamma-inducible protein 16	1.05	2.06	8.12E-13	8.56E-11		3428	Hs.380250
702	204224_s_at	GCH1	GTP cyclohydrolase 1	1.04	2.05	3.36E-07	3.15E-06		2643	Hs.86724
703	203125_x_at	SLC11A2	solute carrier family 11 (proton-coupled	1.04	2.05	1.67E-05	8.68E-05		4891	Hs.505545
704	213448_at			1.04	2.05	2.46E-07	2.39E-06			
705	204118_at	CD48	CD48 molecule	1.04	2.05	0.00036641	0.0012291		962	Hs.243564
706	202533_s_at	DHFR	dihydrofolate reductase	1.04	2.05	1.39E-09	3.28E-08		1719	Hs.592364, Hs.
707	219648_at	MREG	melanoregulin	1.04	2.05	1.47E-08	2.33E-07		55686	Hs.707104, Hs.
708	207386_at	CYP7B1	cytochrome P450, family 7, subfamily B,	1.03	2.05	2.03E-05	0.0001025		9420	Hs.667720
709	202779_s_at	UBE2S	ubiquitin-conjugating enzyme E2S	1.03	2.04	2.11E-07	2.11E-06		27338	Hs.396393
710	219389_at	SUSD4	sushi domain containing 4	1.03	2.04	0.00027045	0.000949		55061	Hs.497841
711	201303_at	EIF4A3	eukaryotic translation initiation factor 4A	1.03	2.04	1.09E-12	1.08E-10		9775	Hs.389649
712	221896_s_at	HIGD1A	HIG1 hypoxia inducible domain family, m	1.03	2.04	3.93E-12	2.88E-10		25994	Hs.593134, Hs.
713	204026_s_at	ZWINT	ZW10 interactor	1.02	2.03	3.48E-05	0.0001631		11130	Hs.591363
714	200733_s_at	PTP4A1	protein tyrosine phosphatase type IVA, m	1.02	2.03	2.58E-08	3.70E-07		7803	Hs.227777, Hs.
715	204240_s_at	SMC2	structural maintenance of chromosomes	1.02	2.03	0.00077598	0.0023677		10592	Hs.119023

	Probe	Symbol	Description	lgFCH	FCH	p	FDR		ENTREZ	UniGene
716	202659_at	PSMB10	proteasome (prosome, macropain) subu	1.02	2.03	5.58E-08	6.93E-07		5699	Hs.9661
717	218448_at	C20orf11	chromosome 20 open reading frame 11	1.02	2.03	6.68E-13	7.30E-11		54994	Hs.353013
718	204656_at	SHB	Src homology 2 domain containing adap	1.02	2.03	1.42E-07	1.50E-06		6461	Hs.521482
719	201118_at	PGD	phosphogluconate dehydrogenase	1.02	2.02	3.62E-08	4.91E-07		5226	Hs.464071
720	205339_at	STIL	SCL/TAL1 interrupting locus	1.02	2.02	6.50E-08	7.85E-07		6491	Hs.525198
721	204058_at	ME1	malic enzyme 1, NADP(+)-dependent, c	1.02	2.02	2.40E-07	2.34E-06		4199	Hs.21160
722	210830_s_at	PON2	paraoxonase 2	1.02	2.02	2.49E-09	5.33E-08		5445	Hs.719159
723	201292_at	TOP2A	topoisomerase (DNA) II alpha 170kDa	1.01	2.02	0.00073683	0.0022649		7153	Hs.156346
724	219503_s_at	TMEM40	transmembrane protein 40	1.01	2.02	1.20E-08	1.96E-07		55287	Hs.475502
725	201853_s_at	CDC25B	cell division cycle 25 homolog B (S. pom	1.01	2.02	1.52E-07	1.60E-06		994	Hs.153752
726	212102_s_at	KPNA6	karyopherin alpha 6 (importin alpha 7)	1.01	2.01	4.03E-05	0.0001848		23633	Hs.470588, Hs.
727	201571_s_at	DCTD	dCMP deaminase	1.01	2.01	3.82E-05	0.0001761		1635	Hs.183850
728	201683_x_at	TOX4	TOX high mobility group box family mem	1.01	2.01	0.00568091	0.0133016		9878	Hs.555910
729	218800_at	SRD5A3	steroid 5 alpha-reductase 3	1.01	2.01	3.91E-05	0.0001797		79644	Hs.39311
730	213988_s_at	SAT1	spermidine/spermine N1-acetyltransfera	1.01	2.01	6.67E-05	0.0002849		6303	Hs.28491
731	210018_x_at	MALT1	mucosa associated lymphoid tissue lym	1	2.01	0.00020168	0.0007385		10892	Hs.601217
732	214205_x_at	GLRX3	glutaredoxin 3	1	2	2.10E-07	2.11E-06		10539	Hs.42644
733	218656_s_at	LHFP	lipoma HMGIC fusion partner	-1	0.5	1.44E-05	7.66E-05		10186	Hs.507798
734	212282_at	TMEM97	transmembrane protein 97	-1	0.5	0.00312662	0.0079315		27346	Hs.199695
735	222277_at			-1	0.5	1.63E-05	8.47E-05			
736	209005_at	FBXL5	F-box and leucine-rich repeat protein 5	-1	0.5	5.54E-05	0.0002424		26234	Hs.643433
737	205583_s_at	ALG13	asparagine-linked glycosylation 13 hom	-1	0.5	2.01E-06	1.45E-05		79868	Hs.443061
738	203556_at	ZHX2	zinc fingers and homeoboxes 2	-1	0.5	1.95E-05	9.90E-05		22882	Hs.658443, Hs.
739	204773_at	IL11RA	interleukin 11 receptor, alpha	-1	0.5	6.87E-05	0.000292		3590	Hs.591088
740	212775_at	OBSL1	obscurin-like 1	-1	0.5	6.51E-07	5.58E-06		23363	Hs.526594
741	37590_g_at			-1	0.5	6.13E-10	1.71E-08			
742	205739_x_at	ZNF107	zinc finger protein 107	-1	0.5	0.0001068	0.0004266		51427	Hs.50216
743	218831_s_at	FCGRT	Fc fragment of IgG, receptor, transporte	-1	0.5	2.35E-06	1.65E-05		2217	Hs.111903
744	204451_at	FZD1	frizzled homolog 1 (Drosophila)	-1	0.5	1.79E-08	2.73E-07		8321	Hs.94234
745	215355_at	POU2F3	POU class 2 homeobox 3	-1	0.5	0.00118776	0.0034421		25833	Hs.227115
746	213605_s_at	LOC100272216	hypothetical LOC100272216	-1	0.5	2.16E-05	0.000108		1E+08	Hs.631974
747	222258_s_at	SH3BP4	SH3-domain binding protein 4	-1.01	0.5	5.91E-09	1.09E-07		23677	Hs.516777
748	202254_at	SIPA1L1	signal-induced proliferation-associated 1	-1.01	0.5	3.38E-06	2.24E-05		26037	Hs.654657
749	204069_at	MEIS1	Meis homeobox 1	-1.01	0.5	4.29E-07	3.87E-06		4211	Hs.526754, Hs.
750	202177_at	GAS6	growth arrest-specific 6	-1.01	0.5	2.08E-08	3.10E-07		2621	Hs.646346
751	201189_s_at	ITPR3	inositol 1,4,5-triphosphate receptor, type	-1.01	0.5	1.09E-14	3.80E-12		3710	Hs.65758
752	203521_s_at	ZNF318	zinc finger protein 318	-1.01	0.5	7.26E-07	6.10E-06		24149	Hs.509718
753	213201_s_at	TNNT1	troponin T type 1 (skeletal, slow)	-1.01	0.5	2.74E-06	1.88E-05		7138	Hs.631558
754	212254_s_at	DST	dystonin	-1.01	0.5	2.04E-08	3.05E-07		667	Hs.631992, Hs.
755	201957_at	PPP1R12B	protein phosphatase 1, regulatory (inhibi	-1.01	0.5	0.00557584	0.0131033		4660	Hs.444403
756	212855_at	DCUN1D4	DCN1, defective in cullin neddylation 1,	-1.01	0.5	1.17E-07	1.28E-06		23142	Hs.605388
757	221958_s_at	GPR177	G protein-coupled receptor 177	-1.01	0.5	3.12E-12	2.45E-10		79971	Hs.647659
758	201506_at	TGFBI	transforming growth factor, beta-induced	-1.01	0.5	1.79E-08	2.73E-07		7045	Hs.369397, Hs.
759	201802_at	SLC29A1	solute carrier family 29 (nucleoside trans	-1.01	0.5	4.56E-05	0.0002053		2030	Hs.25450
760	222217_s_at	SLC27A3	solute carrier family 27 (fatty acid transp	-1.01	0.5	3.11E-09	6.34E-08		11000	Hs.438723
761	37462_i_at	SF3A2	splicing factor 3a, subunit 2, 66kDa	-1.01	0.5	0.00965479	0.0211511		8175	Hs.115232
762	219717_at	DCAF16	DDB1 and CUL4 associated factor 16	-1.01	0.5	0.002166	0.0057545		54876	Hs.614787
763	203509_at	SORL1	sortilin-related receptor, L(DLR class) A	-1.01	0.5	4.72E-07	4.20E-06		6653	Hs.368592
764	203414_at	MMD	monocyte to macrophage differentiation-	-1.01	0.5	0.00011631	0.0004594		23531	Hs.463483, Hs.
765	203510_at	MET	met proto-oncogene (hepatocyte growth	-1.01	0.5	3.28E-08	4.51E-07		4233	Hs.132966
766	222290_at			-1.01	0.5	8.61E-10	2.21E-08			
767	205878_at	POU6F1	POU class 6 homeobox 1	-1.01	0.5	2.60E-08	3.71E-07		5463	Hs.555886
768	48825_at	ING4	inhibitor of growth family, member 4	-1.01	0.5	1.08E-10	4.15E-09		51147	Hs.524210
769	201261_x_at	BGN	biglycan	-1.01	0.5	2.40E-05	0.000119		633	Hs.821
770	218959_at	HOXC10	homeobox C10	-1.01	0.5	0.00045479	0.0014841		3226	Hs.44276
771	208791_at	CLU	clusterin	-1.01	0.49	1.09E-05	6.02E-05		1191	Hs.436657
772	202940_at	WNK1	WNK lysine deficient protein kinase 1	-1.02	0.49	0.00767454	0.0173141		65125	Hs.709894
773	215333_x_at	GSTM1	glutathione S-transferase mu 1	-1.02	0.49	0.00060057	0.0018885		2944	Hs.301961
774	220936_s_at	H2AFJ	H2A histone family, member J	-1.02	0.49	7.82E-06	4.57E-05		55766	Hs.524280, Hs.
775	202732_at	PKIG	protein kinase (cAMP-dependent, cataly	-1.02	0.49	9.89E-10	2.51E-08		11142	Hs.472831
776	209375_at	XPC	xeroderma pigmentosum, complementat	-1.02	0.49	9.91E-07	7.95E-06		7508	Hs.475538
777	203872_at	ACTA1	actin, alpha 1, skeletal muscle	-1.02	0.49	0.01184542	0.0252242		58	Hs.1288
778	205304_s_at	KCNJ8	potassium inwardly-rectifying channel, s	-1.02	0.49	7.83E-05	0.0003283		3764	Hs.102308, Hs.
779	209272_at	NAB1	NGFI-A binding protein 1 (EGR1 binding	-1.02	0.49	4.24E-08	5.56E-07		4664	Hs.570078
780	203355_s_at	PSD3	pleckstrin and Sec7 domain containing 3	-1.02	0.49	0.00017497	0.000656		23362	Hs.434255

	Probe	Symbol	Description	lgFCH	FCH	p	FDR		ENTREZ	UniGene
781	45288_at	ABHD6	abhydrolase domain containing 6	-1.02	0.49	1.82E-07	1.86E-06		57406	Hs.476454
782	205076_s_at	MTMR11	myotubularin related protein 11	-1.02	0.49	2.79E-06	1.91E-05		10903	Hs.425144
783	209541_at	IGF1	insulin-like growth factor 1 (somatomedi	-1.02	0.49	0.02237042	0.0437412		3479	Hs.160562
784	219132_at	PELI2	pellino homolog 2 (Drosophila)	-1.02	0.49	5.19E-05	0.0002297		57161	Hs.657926
785	216264_s_at	LAMB2	laminin, beta 2 (laminin S)	-1.02	0.49	2.74E-07	2.64E-06		3913	Hs.439726
786	204345_at	COL16A1	collagen, type XVI, alpha 1	-1.02	0.49	7.03E-08	8.31E-07		1307	Hs.368921
787	215567_at	FCF1	FCF1 small subunit (SSU) processome	-1.02	0.49	0.00011486	0.0004542		51077	Hs.579828, Hs.
788	209200_at	MEF2C	myocyte enhancer factor 2C	-1.03	0.49	0.00018297	0.0006808		4208	Hs.653394
789	205011_at	VWA5A	von Willebrand factor A domain containi	-1.03	0.49	1.46E-06	1.11E-05		4013	Hs.152944
790	222125_s_at	P4HTM	prolyl 4-hydroxylase, transmembrane (e	-1.03	0.49	1.37E-07	1.46E-06		54681	Hs.654944
791	201008_s_at	TXNIP	thioredoxin interacting protein	-1.03	0.49	0.00039067	0.0012969		10628	Hs.533977, Hs.
792	203221_at	TLE1	transducin-like enhancer of split 1 (E(sp	-1.03	0.49	5.40E-12	3.70E-10		7088	Hs.197320, Hs.
793	201998_at	ST6GAL1	ST6 beta-galactosamide alpha-2,6-sialy	-1.03	0.49	5.15E-08	6.54E-07		6480	Hs.207459
794	212609_s_at	AKT3	v-akt murine thymoma viral oncogene ho	-1.03	0.49	4.34E-07	3.91E-06		10000	Hs.498292
795	211467_s_at	NFIB	nuclear factor I/B	-1.03	0.49	1.60E-05	8.38E-05		4781	Hs.644095
796	201753_s_at	ADD3	adducin 3 (gamma)	-1.03	0.49	6.73E-08	8.05E-07		120	Hs.501012
797	200747_s_at	NUMA1	nuclear mitotic apparatus protein 1	-1.03	0.49	1.82E-08	2.77E-07		4926	Hs.325978
798	218168_s_at	CABC1	chaperone, ABC1 activity of bc1 comple	-1.03	0.49	1.93E-08	2.90E-07		56997	Hs.118241
799	215617_at	SPATS2L	spermatogenesis associated, serine-rich	-1.03	0.49	0.00010248	0.0004116		26010	Hs.120323
800	209101_at	CTGF	connective tissue growth factor	-1.03	0.49	0.00673931	0.0154867		1490	Hs.591346
801	216840_s_at	LAMA2	laminin, alpha 2	-1.03	0.49	0.00253465	0.0066111		3908	Hs.200841
802	201129_at	SFRS7	splicing factor, arginine/serine-rich 7, 35	-1.03	0.49	2.08E-06	1.49E-05		6432	Hs.309090
803	201983_s_at	EGFR	epidermal growth factor receptor (erythr	-1.03	0.49	0.00219237	0.005815		1956	Hs.488293
804	213587_s_at	ATP6V0E2	ATPase, H+ transporting V0 subunit e2	-1.04	0.49	8.57E-08	9.84E-07		155066	Hs.556998, Hs.
805	203264_s_at	ARHGEF9	Cdc42 guanine nucleotide exchange fac	-1.04	0.49	3.75E-08	5.04E-07		23229	Hs.54697
806	205236_x_at	SOD3	superoxide dismutase 3, extracellular	-1.04	0.49	0.00018724	0.0006935		6649	Hs.2420
807	215513_at	HYMAI	hydatidiform mole associated and imprin	-1.04	0.49	0.00027906	0.0009747		57061	Hs.657760, Hs.
808	209737_at	MAGI2	membrane associated guanylate kinase,	-1.04	0.49	5.36E-08	6.72E-07		9863	Hs.603842
809	222121_at	SGEF	Src homology 3 domain-containing guan	-1.04	0.49	1.20E-07	1.30E-06		26084	Hs.240845
810	203801_at	MRPS14	mitochondrial ribosomal protein S14	-1.04	0.49	2.61E-07	2.52E-06		63931	Hs.702192
811	205620_at	F10	coagulation factor X	-1.04	0.49	0.0001504	0.000574		2159	Hs.361463
812	213116_at	NEK3	NIMA (never in mitosis gene a)-related k	-1.04	0.49	1.78E-07	1.83E-06		4752	Hs.409989
813	201188_s_at	ITPR3	inositol 1,4,5-triphosphate receptor, type	-1.04	0.49	1.70E-12	1.53E-10		3710	Hs.65758
814	221564_at	PRMT2	protein arginine methyltransferase 2	-1.04	0.49	4.15E-05	0.0001897		3275	Hs.154163
815	210105_s_at	FYN	FYN oncogene related to SRC, FGR, YE	-1.04	0.49	1.30E-08	2.11E-07		2534	Hs.390567
816	209566_at	INSIG2	insulin induced gene 2	-1.04	0.49	1.51E-08	2.37E-07		51141	Hs.7089
817	217593_at	ZSCAN18	zinc finger and SCAN domain containing	-1.04	0.49	1.15E-06	9.01E-06		65982	Hs.235390
818	202156_s_at	CUGBP2	CUG triplet repeat, RNA binding protein	-1.04	0.49	6.68E-05	0.000285		10659	Hs.309288
819	205528_s_at	RUNX1T1	runt-related transcription factor 1; transl	-1.04	0.49	0.00011226	0.0004452		862	Hs.368431
820	209496_at	RARRES2	retinoic acid receptor responder (tazarot	-1.04	0.49	4.67E-05	0.0002093		5919	Hs.647064
821	564_at	GNA11	guanine nucleotide binding protein (G pr	-1.04	0.49	6.02E-08	7.38E-07		2767	Hs.650575, Hs.
822	203813_s_at	SLIT3	slit homolog 3 (Drosophila)	-1.04	0.49	0.00038262	0.0012741		6586	Hs.604116
823	203874_s_at	SMARCA1	SWI/SNF related, matrix associated, act	-1.04	0.49	5.17E-05	0.0002287		6594	Hs.152292
824	209485_s_at	OSBPL1A	oxysterol binding protein-like 1A	-1.04	0.49	8.74E-05	0.00036		114876	Hs.370725
825	204718_at	EPHB6	EPH receptor B6	-1.05	0.48	1.57E-06	1.18E-05		2051	Hs.380089
826	204669_s_at	RNF24	ring finger protein 24	-1.05	0.48	8.47E-06	4.88E-05		11237	Hs.589884
827	204276_at	TK2	thymidine kinase 2, mitochondrial	-1.05	0.48	2.06E-06	1.48E-05		7084	Hs.512619
828	220486_x_at	TMEM164	transmembrane protein 164	-1.05	0.48	1.07E-07	1.19E-06		84187	Hs.496572, Hs.
829	204431_at	TLE2	transducin-like enhancer of split 2 (E(sp	-1.05	0.48	5.12E-08	6.51E-07		7089	Hs.332173
830	205381_at	LRRC17	leucine rich repeat containing 17	-1.05	0.48	0.00286525	0.0073526		10234	Hs.567412
831	202073_at	OPTN	optineurin	-1.05	0.48	1.06E-08	1.79E-07		10133	Hs.332706
832	213068_at	DPT	dermatopontin	-1.05	0.48	0.0139273	0.0290798		1805	Hs.80552
833	205871_at			-1.05	0.48	0.00373401	0.0092623			
834	202016_at	MEST	mesoderm specific transcript homolog (	-1.05	0.48	0.00144862	0.0040759		4232	Hs.270978
835	209199_s_at	MEF2C	myocyte enhancer factor 2C	-1.05	0.48	0.00956542	0.0209696		4208	Hs.653394
836	204684_at	NPTX1	neuronal pentraxin I	-1.05	0.48	1.82E-05	9.35E-05		4884	Hs.514556, Hs.
837	209169_at	GPM6B	glycoprotein M6B	-1.05	0.48	1.09E-06	8.60E-06		2824	Hs.495710
838	221814_at	GPR124	G protein-coupled receptor 124	-1.05	0.48	1.24E-05	6.75E-05		25960	Hs.274136
839	212930_at	ATP2B1	ATPase, Ca++ transporting, plasma me	-1.05	0.48	6.35E-05	0.0002729		490	Hs.506276
840	206580_s_at	EFEMP2	EGF-containing fibulin-like extracellular	-1.05	0.48	1.12E-05	6.17E-05		30008	Hs.170622
841	213033_s_at	NFIB	nuclear factor I/B	-1.05	0.48	0.00377978	0.009365		4781	Hs.644095
842	218665_at	FZD4	frizzled homolog 4 (Drosophila)	-1.05	0.48	5.81E-08	7.20E-07		8322	Hs.591968
843	204235_s_at	GULP1	GULP, engulfment adaptor PTB domain	-1.06	0.48	0.00166694	0.0045977		51454	Hs.470887, Hs.
844	212950_at	GPR116	G protein-coupled receptor 116	-1.06	0.48	0.00162395	0.0044955		221395	Hs.362806
845	202759_s_at			-1.06	0.48	5.92E-06	3.63E-05			

	Probe	Symbol	Description	lgFCH	FCH	p	FDR		ENTREZ	UniGene
846	214790_at	SENP6	SUMO1/sentrin specific peptidase 6	-1.06	0.48	1.10E-06	8.69E-06		26054	Hs.485784
847	201389_at	ITGA5	integrin, alpha 5 (fibronectin receptor, alp	-1.06	0.48	1.06E-05	5.86E-05		3678	Hs.505654
848	209688_s_at	CCDC93	coiled-coil domain containing 93	-1.06	0.48	1.45E-06	1.10E-05		54520	Hs.107845
849	203020_at	RABGAP1L	RAB GTPase activating protein 1-like	-1.06	0.48	1.14E-07	1.26E-06		9910	Hs.585378
850	35776_at	ITSN1	intersectin 1 (SH3 domain protein)	-1.06	0.48	4.26E-10	1.26E-08		6453	Hs.160324
851	200885_at			-1.06	0.48	3.35E-11	1.57E-09			
852	210964_s_at	GYG2	glycogenin 2	-1.06	0.48	0.00407487	0.0099941		8908	Hs.567381, Hs.
853	209431_s_at	PATZ1	POZ (BTB) and AT hook containing zinc	-1.06	0.48	8.08E-07	6.68E-06		23598	Hs.517557
854	215294_s_at	SMARCA1	SWI/SNF related, matrix associated, act	-1.06	0.48	4.92E-05	0.0002189		6594	Hs.152292
855	209356_x_at	EFEMP2	EGF-containing fibulin-like extracellular r	-1.07	0.48	0.00048049	0.0015534		30008	Hs.170622
856	213657_s_at			-1.07	0.48	4.61E-08	5.99E-07			
857	221870_at	EHD2	EH-domain containing 2	-1.07	0.48	1.62E-06	1.20E-05		30846	Hs.719225
858	211535_s_at	FGFR1	fibroblast growth factor receptor 1	-1.07	0.48	2.29E-07	2.25E-06		2260	Hs.264887
859	222266_at	C19orf2	chromosome 19 open reading frame 2	-1.07	0.48	3.86E-05	0.0001778		8725	Hs.466391
860	201602_s_at	PPP1R12A	protein phosphatase 1, regulatory (inhibi	-1.07	0.48	0.00034573	0.0011695		4659	Hs.49582
861	216037_x_at	TCF7L2	transcription factor 7-like 2 (T-cell specifi	-1.07	0.48	4.32E-09	8.28E-08		6934	Hs.593995
862	210987_x_at	TPM1	tropomyosin 1 (alpha)	-1.07	0.48	6.08E-06	3.71E-05		7168	Hs.133892
863	222116_s_at	TBC1D16	TBC1 domain family, member 16	-1.07	0.48	7.04E-10	1.90E-08		125058	Hs.369819
864	206042_x_at			-1.07	0.48	2.40E-09	5.17E-08			
865	205325_at	PHYHIP	phytanoyl-CoA 2-hydroxylase interacting	-1.07	0.48	3.25E-05	0.0001542		9796	Hs.334688
866	218066_at	SLC12A7	solute carrier family 12 (potassium/chlori	-1.07	0.48	5.26E-11	2.29E-09		10723	Hs.172613
867	209733_at	MID2	midline 2	-1.07	0.48	1.25E-07	1.35E-06		11043	Hs.12256
868	212498_at			-1.07	0.48	3.07E-09	6.29E-08			
869	211458_s_at			-1.07	0.48	1.49E-11	8.18E-10			
870	218613_at	PSD3	pleckstrin and Sec7 domain containing 3	-1.07	0.48	0.00024654	0.0008773		23362	Hs.434255
871	213479_at	NPTX2	neuronal pentraxin II	-1.07	0.48	1.66E-05	8.62E-05		4885	Hs.3281
872	218164_at	SPATA20	spermatogenesis associated 20	-1.07	0.48	3.45E-09	6.94E-08		64847	Hs.103147
873	217853_at	TNS3	tensin 3	-1.08	0.47	4.03E-08	5.33E-07		64759	Hs.520814
874	204066_s_at	AGAP1	ArfGAP with GTPase domain, ankyrin re	-1.08	0.47	4.55E-06	2.90E-05		116987	Hs.435039
875	208454_s_at	PGCP	plasma glutamate carboxypeptidase	-1.08	0.47	1.91E-06	1.39E-05		10404	Hs.156178
876	203159_at	GLS	glutaminase	-1.08	0.47	2.27E-09	4.93E-08		2744	Hs.116448
877	212923_s_at	C6orf145	chromosome 6 open reading frame 145	-1.08	0.47	1.01E-10	3.92E-09		221749	Hs.484500
878	219534_x_at	CDKN1C	cyclin-dependent kinase inhibitor 1C (p5	-1.08	0.47	1.29E-05	6.98E-05		1028	Hs.106070
879	201661_s_at	ACSL3	acyl-CoA synthetase long-chain family m	-1.08	0.47	1.39E-06	1.06E-05		2181	Hs.655772
880	211959_at	IGFBP5	insulin-like growth factor binding protein	-1.08	0.47	1.04E-05	5.80E-05		3488	Hs.607212
881	213258_at	TFPI	tissue factor pathway inhibitor (lipoprotei	-1.08	0.47	6.33E-05	0.0002722		7035	Hs.516578
882	204106_at	TESK1	testis-specific kinase 1	-1.08	0.47	1.60E-10	5.62E-09		7016	Hs.708096
883	206595_at	CST6	cystatin E/M	-1.08	0.47	8.31E-06	4.80E-05		1474	Hs.139389
884	218573_at	MAGEH1	melanoma antigen family H, 1	-1.08	0.47	3.69E-06	2.41E-05		28986	Hs.279819
885	201911_s_at	FARP1	FERM, RhoGEF (ARHGEF) and pleckst	-1.08	0.47	1.53E-05	8.05E-05		10160	Hs.403917
886	205290_s_at	BMP2	bone morphogenetic protein 2	-1.08	0.47	0.01994856	0.0396751		650	Hs.73853
887	36553_at	ASMTL	acetylserotonin O-methyltransferase-like	-1.08	0.47	1.46E-12	1.38E-10		8623	Hs.533514
888	207233_s_at	MITF	microphthalmia-associated transcription	-1.08	0.47	3.13E-06	2.10E-05		4286	Hs.166017, Hs.
889	201719_s_at	EPB41L2	erythrocyte membrane protein band 4.1-	-1.09	0.47	1.40E-08	2.23E-07		2037	Hs.486470
890	218149_s_at	ZNF395	zinc finger protein 395	-1.09	0.47	3.86E-10	1.18E-08		55893	Hs.695998, Hs.
891	212690_at	DDHD2	DDHD domain containing 2	-1.09	0.47	9.43E-06	5.33E-05		23259	Hs.434966
892	203373_at	SOCS2	suppressor of cytokine signaling 2	-1.09	0.47	1.14E-05	6.28E-05		8835	Hs.485572
893	217858_s_at	ARMCX3	armadillo repeat containing, X-linked 3	-1.09	0.47	9.63E-06	5.41E-05		51566	Hs.592225
894	209289_at	NFIB	nuclear factor I/B	-1.09	0.47	1.95E-09	4.34E-08		4781	Hs.644095
895	205042_at	GNE	glucosamine (UDP-N-acetyl)-2-epimeras	-1.09	0.47	2.96E-07	2.82E-06		10020	Hs.5920
896	213792_s_at	INSR	insulin receptor	-1.09	0.47	1.32E-07	1.41E-06		3643	Hs.465744
897	204085_s_at	CLN5	ceroid-lipofuscinosis, neuronal 5	-1.09	0.47	2.37E-05	0.0001178		1203	Hs.30213
898	221274_s_at	LMAN2L	lectin, mannose-binding 2-like	-1.09	0.47	2.70E-06	1.86E-05		81562	Hs.655743
899	210139_s_at	PMP22	peripheral myelin protein 22	-1.09	0.47	3.22E-09	6.54E-08		5376	Hs.372031
900	219436_s_at	EMCN	endomucin	-1.09	0.47	0.0021507	0.0057186		51705	Hs.152913
901	208790_s_at	PTRF	polymerase I and transcript release facto	-1.09	0.47	1.78E-05	9.19E-05		284119	Hs.437191
902	213742_at	SFRS11	splicing factor, arginine/serine-rich 11	-1.09	0.47	1.02E-05	5.67E-05		9295	Hs.479693
903	215016_x_at	DST	dystonin	-1.09	0.47	4.72E-10	1.36E-08		667	Hs.631992, Hs.
904	202561_at	TNKS	tankyrase, TRF1-interacting ankyrin-rela	-1.09	0.47	7.06E-07	5.96E-06		8658	Hs.370267
905	214920_at	THSD7A	thrombospondin, type I, domain containi	-1.1	0.47	0.00064464	0.0020147		221981	Hs.120855, Hs.
906	217164_at			-1.1	0.47	0.00079273	0.0024131			
907	212468_at	SPAG9	sperm associated antigen 9	-1.1	0.47	0.00104714	0.0030874		9043	Hs.463439
908	214925_s_at	SPTAN1	spectrin, alpha, non-erythrocytic 1 (alpha	-1.1	0.47	7.59E-06	4.45E-05		6709	Hs.372331
909	210847_x_at	TNFRSF25	tumor necrosis factor receptor superfam	-1.1	0.47	5.48E-09	1.02E-07		8718	Hs.462529
910	210002_at	GATA6	GATA binding protein 6	-1.1	0.47	0.00091785	0.0027513		2627	Hs.514746

	Probe	Symbol	Description	lgFCH	FCH	p	FDR		ENTREZ	UniGene
911	203929_s_at	MAPT	microtubule-associated protein tau	-1.1	0.47	9.41E-06	5.32E-05		4137	Hs.101174
912	212240_s_at	PIK3R1	phosphoinositide-3-kinase, regulatory su	-1.1	0.47	0.00023503	0.0008419		5295	Hs.132225, Hs.
913	205596_s_at	SMURF2	SMAD specific E3 ubiquitin protein ligase	-1.1	0.47	2.08E-06	1.49E-05		64750	Hs.705442
914	213652_at	PCSK5	proprotein convertase subtilisin/kexin typ	-1.1	0.47	0.0040929	0.0100269		5125	Hs.368542
915	213422_s_at	MXRA8	matrix-remodelling associated 8	-1.1	0.47	0.00023068	0.0008284		54587	Hs.558570
916	213029_at	NFIB	nuclear factor I/B	-1.1	0.47	1.12E-13	2.07E-11		4781	Hs.644095
917	213590_at			-1.1	0.47	9.95E-10	2.52E-08			
918	216331_at	ITGA7	integrin, alpha 7	-1.1	0.47	0.00045626	0.0014882		3679	Hs.524484
919	201842_s_at	EFEMP1	EGF-containing fibulin-like extracellular r	-1.1	0.47	4.38E-07	3.94E-06		2202	Hs.76224
920	65472_at	C2orf68	chromosome 2 open reading frame 68	-1.1	0.47	2.10E-08	3.11E-07		388969	Hs.516159
921	210664_s_at	TFPI	tissue factor pathway inhibitor (lipoprotei	-1.1	0.47	0.0001763	0.0006602		7035	Hs.516578
922	209524_at	HDGFRP3	hepatoma-derived growth factor, related	-1.1	0.47	6.74E-06	4.05E-05		50810	Hs.513954
923	212308_at	CLASP2	cytoplasmic linker associated protein 2	-1.11	0.46	3.97E-08	5.28E-07		23122	Hs.108614
924	220349_s_at	ENGASE	endo-beta-N-acetylglucosaminidase	-1.11	0.46	3.54E-08	4.81E-07		64772	Hs.29288
925	220016_at	AHNAK	AHNAK nucleoprotein	-1.11	0.46	3.89E-07	3.56E-06		79026	Hs.502756
926	218967_s_at	PTER	phosphotriesterase related	-1.11	0.46	0.00018911	0.0006992		9317	Hs.444321, Hs.
927	206884_s_at	SCEL	sciellin	-1.11	0.46	1.83E-08	2.78E-07		8796	Hs.534699
928	201626_at	INSIG1	insulin induced gene 1	-1.11	0.46	0.00016734	0.0006302		3638	Hs.520819
929	212226_s_at	PPAP2B	phosphatidic acid phosphatase type 2B	-1.11	0.46	7.96E-06	4.63E-05		8613	Hs.405156, Hs.
930	204796_at	EML1	echinoderm microtubule associated prot	-1.11	0.46	8.80E-05	0.0003621		2009	Hs.12451
931	220351_at	CCRL1	chemokine (C-C motif) receptor-like 1	-1.11	0.46	0.00014449	0.000554		51554	Hs.310512, Hs.
932	209209_s_at	FERMT2	fermitin family homolog 2 (Drosophila)	-1.12	0.46	0.02090994	0.0413125		10979	Hs.509343
933	218574_s_at	LMCD1	LIM and cysteine-rich domains 1	-1.12	0.46	2.30E-05	0.0001147		29995	Hs.475353
934	222282_at			-1.12	0.46	0.0006298	0.0019727			
935	222126_at	AGFG2	ArfGAP with FG repeats 2	-1.12	0.46	3.81E-06	2.48E-05		3268	Hs.521083
936	215388_s_at			-1.12	0.46	7.31E-07	6.14E-06			
937	201691_s_at	TPD52	tumor protein D52	-1.12	0.46	9.54E-08	1.08E-06		7163	Hs.368433
938	212061_at	SR140	U2-associated SR140 protein	-1.12	0.46	1.19E-07	1.29E-06		23350	Hs.596572
939	218490_s_at	ZNF302	zinc finger protein 302	-1.12	0.46	1.16E-09	2.83E-08		55900	Hs.436350
940	203989_x_at	F2R	coagulation factor II (thrombin) receptor	-1.12	0.46	5.28E-08	6.65E-07		2149	Hs.482562, Hs.
941	201825_s_at	SCCPDH	saccharopine dehydrogenase (putative)	-1.12	0.46	1.43E-09	3.35E-08		51097	Hs.498397
942	205198_s_at	ATP7A	ATPase, Cu++ transporting, alpha polyp	-1.12	0.46	4.60E-08	5.98E-07		538	Hs.496414
943	202512_s_at	ATG5	ATG5 autophagy related 5 homolog (S. c	-1.13	0.46	2.37E-06	1.67E-05		9474	Hs.486063
944	212483_at	NIPBL	Nipped-B homolog (Drosophila)	-1.13	0.46	5.06E-10	1.44E-08		25836	Hs.481927
945	212419_at	ZCCHC24	zinc finger, CCHC domain containing 24	-1.13	0.46	1.58E-07	1.65E-06		219654	Hs.523080
946	208078_s_at			-1.13	0.46	1.10E-05	6.05E-05			
947	218804_at	ANO1	anoctamin 1, calcium activated chloride	-1.13	0.46	8.89E-08	1.02E-06		55107	Hs.503074
948	1598_g_at	GAS6	growth arrest-specific 6	-1.13	0.46	1.09E-08	1.82E-07		2621	Hs.646346
949	218918_at	MAN1C1	mannosidase, alpha, class 1C, member	-1.13	0.46	0.00024451	0.0008708		57134	Hs.197043
950	220425_x_at			-1.13	0.46	0.0001109	0.0004405			
951	204036_at	LPAR1	lysophosphatidic acid receptor 1	-1.13	0.46	2.96E-07	2.82E-06		1902	Hs.126667
952	218471_s_at	BBS1	Bardet-Biedl syndrome 1	-1.13	0.46	4.61E-11	2.06E-09		582	Hs.502915
953	222111_at			-1.14	0.46	3.71E-07	3.43E-06			
954	201009_s_at	TXNIP	thioredoxin interacting protein	-1.14	0.46	8.66E-07	7.08E-06		10628	Hs.533977, Hs.
955	206315_at	CRLF1	cytokine receptor-like factor 1	-1.14	0.45	0.00010035	0.0004047		9244	Hs.114948
956	220170_at	FHL5	four and a half LIM domains 5	-1.14	0.45	0.00069939	0.0021632		9457	Hs.632608
957	221868_at	PAIP2B	poly(A) binding protein interacting protei	-1.14	0.45	2.53E-11	1.24E-09		400961	Hs.416735
958	209866_s_at	LPHN3	latrophilin 3	-1.14	0.45	0.00034068	0.0011544		23284	Hs.28391, Hs.6
959	204290_s_at	ALDH6A1	aldehyde dehydrogenase 6 family, meml	-1.14	0.45	2.76E-05	0.0001342		4329	Hs.293970
960	219563_at	C14orf139	chromosome 14 open reading frame 139	-1.14	0.45	1.69E-08	2.60E-07		79686	Hs.41502
961	200685_at	SFRS11	splicing factor, arginine/serine-rich 11	-1.14	0.45	2.13E-06	1.52E-05		9295	Hs.479693
962	206059_at	ZNF91	zinc finger protein 91	-1.14	0.45	2.46E-08	3.57E-07		7644	Hs.654471
963	212761_at	TCF7L2	transcription factor 7-like 2 (T-cell specifi	-1.14	0.45	6.80E-08	8.11E-07		6934	Hs.593995
964	202668_at	EFNB2	ephrin-B2	-1.14	0.45	6.01E-06	3.67E-05		1948	Hs.149239
965	40524_at	PTPN21	protein tyrosine phosphatase, non-recep	-1.14	0.45	4.70E-08	6.07E-07		11099	Hs.437040
966	222275_at			-1.14	0.45	1.99E-07	2.02E-06			
967	218723_s_at	C13orf15	chromosome 13 open reading frame 15	-1.14	0.45	6.60E-08	7.96E-07		28984	Hs.507866
968	208611_s_at	SPTAN1	spectrin, alpha, non-erythrocytic 1 (alpha	-1.14	0.45	1.78E-07	1.83E-06		6709	Hs.372331
969	202756_s_at	GPC1	glypican 1	-1.14	0.45	2.76E-08	3.92E-07		2817	Hs.328232
970	219999_at	MAN2A2	mannosidase, alpha, class 2A, member	-1.14	0.45	1.49E-12	1.38E-10		4122	Hs.116459
971	204992_s_at	PFN2	profilin 2	-1.15	0.45	5.02E-08	6.41E-07		5217	Hs.91747
972	214783_s_at	ANXA11	annexin A11	-1.15	0.45	7.79E-11	3.13E-09		311	Hs.530291
973	213306_at	MPDZ	multiple PDZ domain protein	-1.15	0.45	0.00048191	0.0015576		8777	Hs.169378
974	204418_x_at	GSTM2	glutathione S-transferase mu 2 (muscle)	-1.15	0.45	9.51E-06	5.36E-05		2946	Hs.279837
975	212594_at	PDCD4	programmed cell death 4 (neoplastic trar	-1.15	0.45	2.22E-10	7.46E-09		27250	Hs.711490

	Probe	Symbol	Description	lgFCH	FCH	p	FDR		ENTREZ	UniGene
976	212509_s_at	MXRA7	matrix-remodelling associated 7	-1.15	0.45	1.23E-09	2.96E-08		439921	Hs.250723, Hs.
977	207177_at	PTGFR	prostaglandin F receptor (FP)	-1.15	0.45	0.00241259	0.0063299		5737	Hs.654365
978	209782_s_at	DBP	D site of albumin promoter (albumin D-b	-1.15	0.45	0.00047891	0.001549		1628	Hs.414480, Hs.
979	209540_at	IGF1	insulin-like growth factor 1 (somatomedi	-1.15	0.45	0.01473907	0.0305565		3479	Hs.160562
980	216339_s_at			-1.15	0.45	0.00120307	0.003481			
981	215768_at			-1.15	0.45	0.00759701	0.0171573			
982	203188_at	B3GNT1	UDP-GlcNAc:betaGal beta-1,3-N-acetylg	-1.15	0.45	2.24E-11	1.14E-09		11041	Hs.8526
983	212239_at	PIK3R1	phosphoinositide-3-kinase, regulatory su	-1.15	0.45	1.31E-09	3.12E-08		5295	Hs.132225, Hs.
984	213290_at	COL6A2	collagen, type VI, alpha 2	-1.15	0.45	2.38E-08	3.47E-07		1292	Hs.420269
985	207711_at	C20orf117	chromosome 20 open reading frame 117	-1.15	0.45	0.00654008	0.0150773		140710	Hs.460807, Hs.
986	212936_at	FAM172A	family with sequence similarity 172, mem	-1.15	0.45	0.00015652	0.0005955		83989	Hs.600086
987	203657_s_at	CTSF	cathepsin F	-1.16	0.45	1.35E-07	1.44E-06		8722	Hs.11590
988	206414_s_at	ASAP2	ArfGAP with SH3 domain, ankyrin repea	-1.16	0.45	2.67E-12	2.18E-10		8853	Hs.555902
989	204404_at	SLC12A2	solute carrier family 12 (sodium/potassiu	-1.16	0.45	0.00035305	0.0011892		6558	Hs.162585, Hs.
990	219778_at	ZFPM2	zinc finger protein, multitype 2	-1.16	0.45	2.81E-06	1.92E-05		23414	Hs.431009
991	202686_s_at	AXL	AXL receptor tyrosine kinase	-1.16	0.45	1.02E-05	5.66E-05		558	Hs.590970
992	219517_at	ELL3	elongation factor RNA polymerase II-like	-1.16	0.45	3.81E-09	7.50E-08		80237	Hs.706346
993	203249_at	EZH1	enhancer of zeste homolog 1 (Drosophil	-1.16	0.45	3.81E-09	7.50E-08		2145	Hs.194669
994	212533_at	WEE1	WEE1 homolog (S. pombe)	-1.16	0.45	8.44E-05	0.0003509		7465	Hs.249441
995	204063_s_at	ULK2	unc-51-like kinase 2 (C. elegans)	-1.16	0.45	2.48E-07	2.41E-06		9706	Hs.168762
996	220197_at	ATP6V0A4	ATPase, H+ transporting, lysosomal V0 s	-1.16	0.45	8.53E-07	6.99E-06		50617	Hs.98967
997	203329_at	PTPRM	protein tyrosine phosphatase, receptor ty	-1.16	0.45	2.02E-07	2.04E-06		5797	Hs.49774
998	204199_at	RALGPS1	Ral GEF with PH domain and SH3 bindi	-1.16	0.45	3.59E-07	3.33E-06		9649	Hs.648175
999	212154_at	SDC2	syndecan 2	-1.16	0.45	0.00104363	0.0030792		6383	Hs.1501, Hs.59
1000	211890_x_at	CAPN3	calpain 3, (p94)	-1.17	0.45	7.31E-08	8.57E-07		825	Hs.143261
1001	204287_at	SYNGR1	synaptogyrin 1	-1.17	0.45	4.33E-09	8.28E-08		9145	Hs.216226
1002	219848_s_at	ZNF432	zinc finger protein 432	-1.17	0.45	2.15E-05	0.0001076		9668	Hs.655934
1003	40446_at	PHF1	PHD finger protein 1	-1.17	0.44	6.77E-08	8.10E-07		5252	Hs.166204
1004	204259_at	MMP7	matrix metallopeptidase 7 (matrilysin, ute	-1.17	0.44	9.37E-05	0.0003818		4316	Hs.2256
1005	203501_at	PGCP	plasma glutamate carboxypeptidase	-1.17	0.44	3.28E-05	0.0001552		10404	Hs.156178
1006	213018_at	GATAD1	GATA zinc finger domain containing 1	-1.17	0.44	3.89E-08	5.22E-07		57798	Hs.21145
1007	203723_at	ITPKB	inositol 1,4,5-trisphosphate 3-kinase B	-1.17	0.44	1.33E-07	1.43E-06		3707	Hs.528087, Hs.
1008	213905_x_at	BGN	biglycan	-1.17	0.44	0.00021754	0.0007887		633	Hs.821
1009	210986_s_at	TPM1	tropomyosin 1 (alpha)	-1.17	0.44	2.22E-07	2.20E-06		7168	Hs.133892
1010	211737_x_at	PTN	pleiotrophin	-1.17	0.44	0.00036296	0.0012181		5764	Hs.371249
1011	204671_s_at	ANKRD6	ankyrin repeat domain 6	-1.17	0.44	1.52E-06	1.15E-05		22881	Hs.702213
1012	200974_at	ACTA2	actin, alpha 2, smooth muscle, aorta	-1.18	0.44	1.61E-05	8.41E-05		59	Hs.500483
1013	208016_s_at	AGTR1	angiotensin II receptor, type 1	-1.18	0.44	0.00045882	0.0014954		185	Hs.477887, Hs.
1014	205110_s_at	FGF13	fibroblast growth factor 13	-1.18	0.44	1.41E-05	7.49E-05		2258	Hs.6540, Hs.71
1015	212423_at	ZCCHC24	zinc finger, CCHC domain containing 24	-1.18	0.44	1.96E-07	1.99E-06		219654	Hs.523080
1016	203167_at	TIMP2	TIMP metallopeptidase inhibitor 2	-1.18	0.44	5.92E-05	0.0002571		7077	Hs.633514
1017	202036_s_at	SFRP1	secreted frizzled-related protein 1	-1.18	0.44	0.00283031	0.0072731		6422	Hs.713546
1018	202125_s_at	TRAK2	trafficking protein, kinesin binding 2	-1.18	0.44	6.34E-06	3.84E-05		66008	Hs.152774
1019	203464_s_at	EPN2	epsin 2	-1.18	0.44	9.60E-12	5.94E-10		22905	Hs.715517
1020	206637_at	P2RY14	purinergic receptor P2Y, G-protein coupl	-1.18	0.44	0.00013225	0.0005138		9934	Hs.2465
1021	220692_at			-1.18	0.44	1.52E-06	1.15E-05			
1022	219949_at	LRRC2	leucine rich repeat containing 2	-1.18	0.44	0.0013719	0.0038904		79442	Hs.657345
1023	212695_at	CRY2	cryptochrome 2 (photolyase-like)	-1.19	0.44	9.78E-14	1.96E-11		1408	Hs.532491
1024	212813_at	JAM3	junctional adhesion molecule 3	-1.19	0.44	1.01E-10	3.94E-09		83700	Hs.150718
1025	210944_s_at	CAPN3	calpain 3, (p94)	-1.19	0.44	3.30E-08	4.53E-07		825	Hs.143261
1026	213939_s_at	RUFY3	RUN and FYVE domain containing 3	-1.19	0.44	7.04E-05	0.000298		22902	Hs.713172
1027	202660_at	ITPR2	inositol 1,4,5-triphosphate receptor, type	-1.19	0.44	9.29E-07	7.52E-06		3709	Hs.512235
1028	219025_at	CD248	CD248 molecule, endosialin	-1.19	0.44	0.0001171	0.0004619		57124	Hs.195727
1029	205455_at	MST1R	macrophage stimulating 1 receptor (c-me	-1.19	0.44	1.72E-06	1.27E-05		4486	Hs.517973
1030	214909_s_at	DDAH2	dimethylarginine dimethylaminohydrolas	-1.19	0.44	6.73E-10	1.85E-08		23564	Hs.247362
1031	209865_at	SLC35A3	solute carrier family 35 (UDP-N-acetylglu	-1.19	0.44	4.12E-05	0.0001883		23443	Hs.448979
1032	212458_at	SPRED2	sprouty-related, EVH1 domain containing	-1.19	0.44	1.08E-06	8.56E-06		200734	Hs.59332
1033	202630_at	APPBP2	amyloid beta precursor protein (cytoplas	-1.19	0.44	5.48E-08	6.85E-07		10513	Hs.84084
1034	208789_at	PTRF	polymerase I and transcript release facto	-1.19	0.44	1.94E-11	1.00E-09		284119	Hs.437191
1035	205006_s_at	NMT2	N-myristoyltransferase 2	-1.19	0.44	6.91E-09	1.23E-07		9397	Hs.60339
1036	202149_at	NEDD9	neural precursor cell expressed, develop	-1.2	0.44	1.43E-06	1.09E-05		4739	Hs.37982
1037	209590_at	BMP7	bone morphogenetic protein 7	-1.2	0.44	2.77E-06	1.90E-05		655	Hs.473163
1038	212343_at	YIPF6	Yip1 domain family, member 6	-1.2	0.44	1.50E-12	1.38E-10		286451	Hs.700646, Hs.
1039	45297_at	EHD2	EH-domain containing 2	-1.2	0.44	7.62E-06	4.47E-05		30846	Hs.719225
1040	213317_at	CLIC5	chloride intracellular channel 5	-1.2	0.44	0.00021875	0.0007929		53405	Hs.485489

	Probe	Symbol	Description	lgFCH	FCH	p	FDR		ENTREZ	UniGene
1041	204846_at	CP	ceruloplasmin (ferroxidase)	-1.2	0.44	0.00224308	0.0059372		1356	Hs.558314
1042	202350_s_at	MATN2	matrilin 2	-1.2	0.44	1.40E-08	2.24E-07		4147	Hs.189445, Hs.
1043	211538_s_at	HSPA2	heat shock 70kDa protein 2	-1.2	0.43	2.42E-08	3.52E-07		3306	Hs.719230
1044	204793_at	GPRASP1	G protein-coupled receptor associated s	-1.2	0.43	1.05E-10	4.02E-09		9737	Hs.710048
1045	212080_at	MLL	myeloid/lymphoid or mixed-lineage leuke	-1.2	0.43	3.95E-08	5.27E-07		4297	Hs.258855
1046	212731_at	ANKRD46	ankyrin repeat domain 46	-1.2	0.43	1.82E-05	9.36E-05		157567	Hs.530199
1047	202920_at	ANK2	ankyrin 2, neuronal	-1.2	0.43	0.00011412	0.0004516		287	Hs.620557
1048	209120_at	NR2F2	nuclear receptor subfamily 2, group F, m	-1.2	0.43	2.46E-06	1.72E-05		7026	Hs.657455
1049	215268_at	KIAA0754	KIAA0754	-1.2	0.43	0.00046129	0.0015019		643314	Hs.658760
1050	212992_at	AHNAK2	AHNAK nucleoprotein 2	-1.2	0.43	4.65E-13	5.75E-11		113146	Hs.441783
1051	31637_s_at			-1.21	0.43	5.95E-05	0.0002579			
1052	205752_s_at	GSTM5	glutathione S-transferase mu 5	-1.21	0.43	3.97E-06	2.58E-05		2949	Hs.75652
1053	217891_at	C16orf58	chromosome 16 open reading frame 58	-1.21	0.43	6.18E-06	3.76E-05		64755	Hs.9003
1054	203794_at	CDC42BPA	CDC42 binding protein kinase alpha (DM	-1.21	0.43	1.67E-08	2.57E-07		8476	Hs.35433
1055	213658_at			-1.21	0.43	7.04E-09	1.25E-07			
1056	202609_at	EPS8	epidermal growth factor receptor pathwa	-1.21	0.43	2.60E-06	1.80E-05		2059	Hs.591160
1057	207302_at	SGCG	sarcoglycan, gamma (35kDa dystrophin-	-1.21	0.43	1.62E-06	1.20E-05		6445	Hs.37167
1058	202662_s_at	ITPR2	inositol 1,4,5-triphosphate receptor, type	-1.21	0.43	1.02E-09	2.56E-08		3709	Hs.512235
1059	32259_at	EZH1	enhancer of zeste homolog 1 (Drosophil	-1.21	0.43	1.35E-10	4.89E-09		2145	Hs.194669
1060	218370_s_at	S100PBP	S100P binding protein	-1.22	0.43	7.86E-05	0.0003293		64766	Hs.440880
1061	217862_at	PIAS1	protein inhibitor of activated STAT, 1	-1.22	0.43	5.97E-05	0.0002587		8554	Hs.162458
1062	207057_at	SLC16A7	solute carrier family 16, member 7 (mon	-1.22	0.43	0.00267417	0.0069203		9194	Hs.439643
1063	203381_s_at	APOE	apolipoprotein E	-1.22	0.43	1.43E-09	3.35E-08		348	Hs.654439
1064	209335_at	DCN	decorin	-1.22	0.43	1.25E-07	1.35E-06		1634	Hs.718429
1065	219511_s_at	SNCAIP	synuclein, alpha interacting protein	-1.22	0.43	2.14E-08	3.17E-07		9627	Hs.426463
1066	201810_s_at	SH3BP5	SH3-domain binding protein 5 (BTK-ass	-1.22	0.43	7.69E-05	0.0003226		9467	Hs.257761, Hs.
1067	215129_at	PIK3C2G	phosphoinositide-3-kinase, class 2, gam	-1.22	0.43	3.77E-06	2.46E-05		5288	Hs.22500
1068	209355_s_at	PPAP2B	phosphatidic acid phosphatase type 2B	-1.22	0.43	9.23E-06	5.23E-05		8613	Hs.405156, Hs.
1069	221589_s_at	ALDH6A1	aldehyde dehydrogenase 6 family, mem	-1.22	0.43	6.65E-06	4.00E-05		4329	Hs.293970
1070	205463_s_at	PDGFA	platelet-derived growth factor alpha poly	-1.22	0.43	3.12E-08	4.32E-07		5154	Hs.535898
1071	217901_at	DSG2	desmoglein 2	-1.23	0.43	1.19E-08	1.95E-07		1829	Hs.412597
1072	219525_at	SLC47A1	solute carrier family 47, member 1	-1.23	0.43	3.04E-07	2.88E-06		55244	Hs.232054
1073	213032_at	NFIB	nuclear factor I/B	-1.23	0.43	2.00E-06	1.44E-05		4781	Hs.644095
1074	203812_at	SLIT3	slit homolog 3 (Drosophila)	-1.23	0.43	3.03E-05	0.0001454		6586	Hs.604116
1075	202724_s_at	FOXO1	forkhead box O1	-1.23	0.43	3.06E-11	1.45E-09		2308	Hs.370666
1076	203542_s_at	KLF9	Kruppel-like factor 9	-1.23	0.43	0.00012868	0.0005021		687	Hs.150557, Hs.
1077	218437_s_at	LZTFL1	leucine zipper transcription factor-like 1	-1.23	0.43	1.38E-07	1.47E-06		54585	Hs.30824
1078	204755_x_at	HLF	hepatic leukemia factor	-1.23	0.43	8.57E-05	0.0003552		3131	Hs.196952
1079	202071_at	SDC4	syndecan 4	-1.23	0.43	7.28E-14	1.62E-11		6385	Hs.632267
1080	215017_s_at	FNBP1L	formin binding protein 1-like	-1.23	0.43	1.76E-08	2.69E-07		54874	Hs.134060
1081	221599_at	C11orf67	chromosome 11 open reading frame 67	-1.23	0.43	8.77E-13	9.17E-11		28971	Hs.503357
1082	202972_s_at	FAM13A	family with sequence similarity 13, mem	-1.23	0.43	7.72E-10	2.03E-08		10144	Hs.97270
1083	216080_s_at	FADS3	fatty acid desaturase 3	-1.23	0.43	0.00017752	0.0006638		3995	Hs.21765
1084	202022_at	ALDOC	aldolase C, fructose-bisphosphate	-1.24	0.42	7.14E-07	6.01E-06		230	Hs.155247
1085	213703_at	LOC150759	hypothetical protein LOC150759	-1.24	0.42	4.30E-06	2.76E-05		150759	Hs.646318, Hs.
1086	217627_at	ZNF573	zinc finger protein 573	-1.24	0.42	7.88E-08	9.18E-07		126231	Hs.531262
1087	202328_s_at	PKD1	polycystic kidney disease 1 (autosomal d	-1.24	0.42	4.47E-08	5.83E-07		5310	Hs.75813
1088	204589_at	NUAK1	NUAK family, SNF1-like kinase, 1	-1.24	0.42	1.13E-07	1.25E-06		9891	Hs.719171
1089	205383_s_at	ZBTB20	zinc finger and BTB domain containing 2	-1.24	0.42	7.39E-07	6.20E-06		26137	Hs.655108
1090	219093_at	PID1	phosphotyrosine interaction domain con	-1.24	0.42	1.00E-07	1.13E-06		55022	Hs.715695
1091	207996_s_at	C18orf1	chromosome 18 open reading frame 1	-1.24	0.42	3.37E-06	2.24E-05		753	Hs.149363
1092	212993_at	NACC2	NACC family member 2, BEN and BTB (	-1.24	0.42	6.26E-07	5.39E-06		138151	Hs.112895
1093	207669_at	KRT83	keratin 83	-1.24	0.42	0.01440389	0.0299292		3889	Hs.658118
1094	212675_s_at	CEP68	centrosomal protein 68kDa	-1.25	0.42	3.98E-07	3.62E-06		23177	Hs.709257
1095	205730_s_at	ABLIM3	actin binding LIM protein family, member	-1.25	0.42	5.22E-07	4.57E-06		22885	Hs.49688
1096	221523_s_at	RRAGD	Ras-related GTP binding D	-1.25	0.42	2.09E-07	2.09E-06		58528	Hs.31712
1097	212442_s_at	LASS6	LAG1 homolog, ceramide synthase 6	-1.25	0.42	2.88E-07	2.75E-06		253782	Hs.506829, Hs.
1098	212099_at	RHOB	ras homolog gene family, member B	-1.25	0.42	3.20E-08	4.42E-07		388	Hs.502876
1099	213348_at	CDKN1C	cyclin-dependent kinase inhibitor 1C (p5	-1.25	0.42	1.25E-05	6.83E-05		1028	Hs.106070
1100	213329_at	SRGAP2	SLIT-ROBO Rho GTPase activating prot	-1.25	0.42	2.81E-09	5.90E-08		23380	Hs.497575
1101	212446_s_at	LASS6	LAG1 homolog, ceramide synthase 6	-1.25	0.42	1.46E-10	5.18E-09		253782	Hs.506829, Hs.
1102	212741_at	MAOA	monoamine oxidase A	-1.25	0.42	1.30E-08	2.10E-07		4128	Hs.183109
1103	209068_at	HNRPDL	heterogeneous nuclear ribonucleoprotei	-1.25	0.42	2.64E-13	3.73E-11		9987	Hs.527105
1104	222101_s_at	DCHS1	dachsous 1 (Drosophila)	-1.25	0.42	5.20E-07	4.57E-06		8642	Hs.199850
1105	203151_at	MAP1A	microtubule-associated protein 1A	-1.25	0.42	1.97E-06	1.42E-05		4130	Hs.194301, Hs.

	Probe	Symbol	Description	lgFCH	FCH	p	FDR		ENTREZ	UniGene
1106	201117_s_at	CPE	carboxypeptidase E	-1.25	0.42	2.87E-06	1.96E-05		1363	Hs.75360
1107	218162_at	OLFML3	olfactomedin-like 3	-1.26	0.42	0.00014111	0.0005432		56944	Hs.9315
1108	211354_s_at	LEPR	leptin receptor	-1.26	0.42	0.0002985	0.0010333		3953	Hs.705413
1109	215303_at	DCLK1	doublecortin-like kinase 1	-1.26	0.42	0.00083446	0.0025234		9201	Hs.507755, Hs.
1110	218211_s_at	MLPH	melanophilin	-1.26	0.42	1.51E-08	2.37E-07		79083	Hs.102406
1111	214235_at			-1.26	0.42	0.00134909	0.0038393			
1112	221016_s_at	TCF7L1	transcription factor 7-like 1 (T-cell specifi	-1.26	0.42	1.89E-06	1.37E-05		83439	Hs.516297
1113	218934_s_at	HSPB7	heat shock 27kDa protein family, membe	-1.26	0.42	1.86E-06	1.35E-05		27129	Hs.502612
1114	219167_at	RASL12	RAS-like, family 12	-1.26	0.42	1.18E-06	9.19E-06		51285	Hs.27018
1115	203757_s_at	CEACAM6	carcinoembryonic antigen-related cell ad	-1.27	0.42	0.00828618	0.0185026		4680	Hs.466814
1116	203404_at	ARMCX2	armadillo repeat containing, X-linked 2	-1.27	0.42	3.44E-05	0.0001615		9823	Hs.48924
1117	204037_at	LPAR1	lysophosphatidic acid receptor 1	-1.27	0.42	9.01E-08	1.03E-06		1902	Hs.126667
1118	202500_at	DNAJB2	DnaJ (Hsp40) homolog, subfamily B, me	-1.27	0.41	9.83E-09	1.68E-07		3300	Hs.77768
1119	209522_s_at	CRAT	carnitine acetyltransferase	-1.27	0.41	9.32E-05	0.0003802		1384	Hs.12068
1120	206695_x_at	ZNF43	zinc finger protein 43	-1.27	0.41	5.08E-07	4.48E-06		7594	Hs.534365
1121	202172_at	VEZF1	vascular endothelial zinc finger 1	-1.27	0.41	9.55E-08	1.08E-06		7716	Hs.463569
1122	216320_x_at	MST1	macrophage stimulating 1 (hepatocyte g	-1.27	0.41	0.00012603	0.0004923		4485	Hs.349110, Hs.
1123	202054_s_at	ALDH3A2	aldehyde dehydrogenase 3 family, meml	-1.28	0.41	2.91E-11	1.40E-09		224	Hs.499886
1124	203543_s_at	KLF9	Kruppel-like factor 9	-1.28	0.41	2.95E-06	2.00E-05		687	Hs.150557, Hs.
1125	212486_s_at	FYN	FYN oncogene related to SRC, FGR, YE	-1.28	0.41	0.00104417	0.0030801		2534	Hs.390567
1126	209210_s_at	FERMT2	fermitin family homolog 2 (Drosophila)	-1.28	0.41	5.16E-05	0.0002283		10979	Hs.509343
1127	204501_at	NOV	nephroblastoma overexpressed gene	-1.28	0.41	2.53E-05	0.0001246		4856	Hs.235935
1128	220917_s_at	WDR19	WD repeat domain 19	-1.29	0.41	3.73E-07	3.44E-06		57728	Hs.438482
1129	221552_at	ABHD6	abhydrolase domain containing 6	-1.29	0.41	6.79E-06	4.07E-05		57406	Hs.476454
1130	202548_s_at	ARHGEF7	Rho guanine nucleotide exchange factor	-1.29	0.41	1.80E-09	4.10E-08		8874	Hs.508738
1131	203088_at	FBLN5	fibulin 5	-1.29	0.41	5.47E-05	0.00024		10516	Hs.332708
1132	209867_s_at	LPHN3	latrophilin 3	-1.29	0.41	8.67E-05	0.0003584		23284	Hs.28391, Hs.6
1133	204401_at	KCNN4	potassium intermediate/small conductan	-1.29	0.41	1.08E-07	1.20E-06		3783	Hs.10082
1134	221748_s_at	TNS1	tensin 1	-1.29	0.41	1.10E-08	1.83E-07		7145	Hs.471381
1135	213693_s_at	MUC1	mucin 1, cell surface associated	-1.29	0.41	0.00024957	0.0008866		4582	Hs.89603
1136	211998_at	H3F3B	H3 histone, family 3B (H3.3B)	-1.29	0.41	1.08E-07	1.19E-06		3021	Hs.180877, Hs.
1137	219682_s_at	TBX3	T-box 3	-1.29	0.41	1.13E-07	1.25E-06		6926	Hs.714737
1138	219825_at	CYP26B1	cytochrome P450, family 26, subfamily E	-1.3	0.41	1.32E-08	2.12E-07		56603	Hs.91546
1139	220161_s_at	EPB41L4B	erythrocyte membrane protein band 4.1	-1.3	0.41	3.83E-08	5.14E-07		54566	Hs.591901
1140	214234_s_at			-1.3	0.41	2.11E-05	0.000106			
1141	203081_at	CTNNBIP1	catenin, beta interacting protein 1	-1.3	0.41	7.53E-10	1.99E-08		56998	Hs.463759
1142	203705_s_at	FZD7	frizzled homolog 7 (Drosophila)	-1.31	0.4	4.75E-05	0.000212		8324	Hs.173859
1143	209604_s_at	GATA3	GATA binding protein 3	-1.31	0.4	1.15E-11	6.80E-10		2625	Hs.524134
1144	219423_x_at	TNFRSF25	tumor necrosis factor receptor superfam	-1.31	0.4	2.92E-08	4.11E-07		8718	Hs.462529
1145	205969_at	AADAC	arylacetamide deacetylase (esterase)	-1.31	0.4	0.01254786	0.0265172		13	Hs.506908
1146	202995_s_at	FBLN1	fibulin 1	-1.31	0.4	1.20E-05	6.57E-05		2192	Hs.24601, Hs.5
1147	213895_at	EMP1	epithelial membrane protein 1	-1.31	0.4	3.64E-06	2.39E-05		2012	Hs.707901
1148	221675_s_at	CHPT1	choline phosphotransferase 1	-1.31	0.4	8.57E-12	5.49E-10		56994	Hs.293077
1149	213150_at	HOXA10	homeobox A10	-1.31	0.4	7.00E-07	5.90E-06		3206	Hs.110637
1150	208868_s_at	GABARAPL1	GABA(A) receptor-associated protein lik	-1.31	0.4	5.66E-07	4.92E-06		23710	Hs.524250
1151	203304_at	BAMBI	BMP and activin membrane-bound inhib	-1.31	0.4	1.35E-06	1.03E-05		25805	Hs.533336
1152	203698_s_at	FRZB	frizzled-related protein	-1.31	0.4	7.70E-07	6.41E-06		2487	Hs.128453
1153	205525_at	CALD1	caldesmon 1	-1.31	0.4	0.00042141	0.0013865		800	Hs.490203
1154	203628_at	IGF1R	insulin-like growth factor 1 receptor	-1.31	0.4	0.00033541	0.00114		3480	Hs.643120, Hs.
1155	204788_s_at	PPOX	protoporphyrinogen oxidase	-1.31	0.4	7.30E-11	2.97E-09		5498	Hs.517373
1156	203571_s_at	C10orf116	chromosome 10 open reading frame 116	-1.31	0.4	3.73E-09	7.39E-08		10974	Hs.642660, Hs.
1157	212914_at	CBX7	chromobox homolog 7	-1.32	0.4	4.13E-09	7.97E-08		23492	Hs.356416
1158	204604_at	PFTK1	PFTAIRE protein kinase 1	-1.32	0.4	5.25E-08	6.63E-07		5218	Hs.430742
1159	203096_s_at	RAPGEF2	Rap guanine nucleotide exchange factor	-1.32	0.4	8.32E-05	0.0003464		9693	Hs.113912
1160	205277_at	PRDM2	PR domain containing 2, with ZNF doma	-1.32	0.4	5.58E-08	6.93E-07		7799	Hs.371823
1161	202291_s_at	MGP	matrix Gla protein	-1.32	0.4	4.01E-06	2.61E-05		4256	Hs.365706
1162	204800_s_at	DHRS12	dehydrogenase/reductase (SDR family)	-1.32	0.4	3.81E-09	7.50E-08		79758	Hs.266728
1163	212560_at	SORL1	sortilin-related receptor, L(DLR class) A r	-1.32	0.4	3.58E-05	0.0001668		6653	Hs.368592
1164	204686_at	IRS1	insulin receptor substrate 1	-1.32	0.4	1.39E-06	1.06E-05		3667	Hs.471508
1165	219615_s_at	KCNK5	potassium channel, subfamily K, membe	-1.32	0.4	5.50E-07	4.80E-06		8645	Hs.444448
1166	204422_s_at	FGF2	fibroblast growth factor 2 (basic)	-1.32	0.4	1.21E-05	6.63E-05		2247	Hs.284244
1167	205933_at	SETBP1	SET binding protein 1	-1.32	0.4	1.32E-07	1.42E-06		26040	Hs.435458
1168	206858_s_at	HOXC6	homeobox C6	-1.33	0.4	1.03E-07	1.15E-06		3223	Hs.549040
1169	205802_at	TRPC1	transient receptor potential cation chann	-1.33	0.4	4.01E-09	7.77E-08		7220	Hs.250687
1170	213071_at	DPT	dermatopontin	-1.33	0.4	0.0001352	0.0005232		1805	Hs.80552

	Probe	Symbol	Description	lgFCH	FCH	p	FDR		ENTREZ	UniGene
1171	208335_s_at	DARC	Duffy blood group, chemokine receptor	-1.33	0.4	5.35E-08	6.72E-07		2532	Hs.153381
1172	219970_at	GIPC2	GIPC PDZ domain containing family, me	-1.33	0.4	3.03E-07	2.87E-06		54810	Hs.659356
1173	204352_at	TRAF5	TNF receptor-associated factor 5	-1.33	0.4	5.47E-06	3.39E-05		7188	Hs.523930
1174	205609_at	ANGPT1	angiopoietin 1	-1.33	0.4	0.00019677	0.0007238		284	Hs.369675
1175	202982_s_at			-1.34	0.4	1.25E-05	6.83E-05			
1176	209185_s_at	IRS2	insulin receptor substrate 2	-1.34	0.4	5.07E-10	1.44E-08		8660	Hs.442344
1177	202342_s_at	TRIM2	tripartite motif-containing 2	-1.34	0.4	7.64E-06	4.47E-05		23321	Hs.435711
1178	212651_at	RHOBTB1	Rho-related BTB domain containing 1	-1.34	0.39	7.26E-09	1.29E-07		9886	Hs.148670
1179	205422_s_at	ITGBL1	integrin, beta-like 1 (with EGF-like repea	-1.34	0.39	7.29E-05	0.0003076		9358	Hs.696554
1180	209121_x_at	NR2F2	nuclear receptor subfamily 2, group F, m	-1.34	0.39	7.34E-06	4.33E-05		7026	Hs.657455
1181	206159_at	GDF10	growth differentiation factor 10	-1.34	0.39	0.00106127	0.0031233		2662	Hs.2171
1182	220432_s_at	CYP39A1	cytochrome P450, family 39, subfamily A	-1.35	0.39	4.56E-06	2.91E-05		51302	Hs.387367
1183	204894_s_at	AOC3	amine oxidase, copper containing 3 (vas	-1.35	0.39	0.00015829	0.0006012		8639	Hs.198241
1184	205594_at	ZNF652	zinc finger protein 652	-1.35	0.39	9.68E-05	0.0003923		22834	Hs.463375
1185	36554_at	ASMTL	acetylserotonin O-methyltransferase-like	-1.35	0.39	3.99E-10	1.20E-08		8623	Hs.533514
1186	218618_s_at	FNDC3B	fibronectin type III domain containing 3B	-1.35	0.39	0.00605592	0.0140849		64778	Hs.159430
1187	205051_s_at	KIT	v-kit Hardy-Zuckerman 4 feline sarcoma	-1.36	0.39	3.48E-07	3.25E-06		3815	Hs.479754
1188	210517_s_at	AKAP12	A kinase (PRKA) anchor protein 12	-1.36	0.39	6.60E-05	0.0002825		9590	Hs.371240
1189	205614_x_at	MST1	macrophage stimulating 1 (hepatocyte g	-1.36	0.39	5.80E-06	3.56E-05		4485	Hs.349110, Hs.
1190	209184_s_at	IRS2	insulin receptor substrate 2	-1.36	0.39	6.00E-12	4.02E-10		8660	Hs.442344
1191	207961_x_at	MYH11	myosin, heavy chain 11, smooth muscle	-1.36	0.39	2.95E-06	2.00E-05		4629	Hs.460109
1192	213093_at	PRKCA	protein kinase C, alpha	-1.36	0.39	9.07E-08	1.03E-06		5578	Hs.531704, Hs.
1193	211745_x_at			-1.36	0.39	0.0251448	0.0482924			
1194	216699_s_at	KLK1	kallikrein 1	-1.36	0.39	5.21E-11	2.28E-09		3816	Hs.123107
1195	204032_at	BCAR3	breast cancer anti-estrogen resistance 3	-1.36	0.39	4.55E-13	5.68E-11		8412	Hs.36958
1196	218638_s_at	SPON2	spondin 2, extracellular matrix protein	-1.36	0.39	1.44E-07	1.52E-06		10417	Hs.302963
1197	206373_at	ZIC1	Zic family member 1 (odd-paired homolo	-1.36	0.39	0.00098418	0.0029252		7545	Hs.598590, Hs.
1198	219263_at	RNF128	ring finger protein 128	-1.36	0.39	6.89E-06	4.11E-05		79589	Hs.496542
1199	205792_at	WISP2	WNT1 inducible signaling pathway prote	-1.36	0.39	0.0007646	0.0023391		8839	Hs.592145
1200	215537_x_at	DDAH2	dimethylarginine dimethylaminohydrolas	-1.36	0.39	1.50E-07	1.58E-06		23564	Hs.247362
1201	204964_s_at	SSPN	sarcospan (Kras oncogene-associated g	-1.37	0.39	1.36E-05	7.30E-05		8082	Hs.183428
1202	215073_s_at	NR2F2	nuclear receptor subfamily 2, group F, m	-1.37	0.39	8.84E-06	5.06E-05		7026	Hs.657455
1203	209406_at	BAG2	BCL2-associated athanogene 2	-1.37	0.39	0.00146191	0.0041079		9532	Hs.719303
1204	210809_s_at	POSTN	periostin, osteoblast specific factor	-1.37	0.39	1.89E-07	1.93E-06		10631	Hs.136348
1205	202259_s_at	N4BP2L2	NEDD4 binding protein 2-like 2	-1.37	0.39	3.62E-07	3.36E-06		10443	Hs.507680
1206	208733_at	RAB2A	RAB2A, member RAS oncogene family	-1.37	0.39	0.00024157	0.0008615		5862	Hs.369017
1207	217087_at	C1orf68	chromosome 1 open reading frame 68	-1.37	0.39	1.34E-06	1.03E-05		1E+08	Hs.601077
1208	212504_at	DIP2C	DIP2 disco-interacting protein 2 homolog	-1.37	0.39	2.42E-05	0.0001196		22982	Hs.432397
1209	210762_s_at	DLC1	deleted in liver cancer 1	-1.37	0.39	1.05E-05	5.82E-05		10395	Hs.134296
1210	203006_at	INPP5A	inositol polyphosphate-5-phosphatase, 4	-1.37	0.39	1.42E-11	7.91E-10		3632	Hs.523360, Hs.
1211	212764_at	ZEB1	zinc finger E-box binding homeobox 1	-1.38	0.39	6.19E-07	5.34E-06		6935	Hs.124503
1212	214036_at	EFNA5	ephrin-A5	-1.38	0.38	9.24E-06	5.23E-05		1946	Hs.288741
1213	204749_at	NAP1L3	nucleosome assembly protein 1-like 3	-1.38	0.38	5.43E-05	0.0002388		4675	Hs.21365
1214	205328_at	CLDN10	claudin 10	-1.38	0.38	0.0011419	0.0033256		9071	Hs.534377, Hs.
1215	219935_at	ADAMTS5	ADAM metallopeptidase with thrombosp	-1.38	0.38	0.00317951	0.008053		11096	Hs.58324
1216	205433_at	BCHE	butyrylcholinesterase	-1.38	0.38	0.0010599	0.00312		590	Hs.420483
1217	209758_s_at	MFAP5	microfibrillar associated protein 5	-1.38	0.38	0.00289381	0.0074155		8076	Hs.512842
1218	220037_s_at	LYVE1	lymphatic vessel endothelial hyaluronan	-1.38	0.38	0.00074364	0.0022831		10894	Hs.655332, Hs.
1219	216511_s_at	TCF7L2	transcription factor 7-like 2 (T-cell specifi	-1.38	0.38	7.70E-06	4.50E-05		6934	Hs.593995
1220	214764_at	RRP15	ribosomal RNA processing 15 homolog (	-1.38	0.38	3.38E-09	6.82E-08		51018	Hs.660109
1221	205407_at	RECK	reversion-inducing-cysteine-rich protein	-1.39	0.38	7.40E-05	0.0003115		8434	Hs.388918
1222	202242_at	TSPAN7	tetraspanin 7	-1.39	0.38	1.07E-05	5.93E-05		7102	Hs.441664
1223	209842_at	SOX10	SRY (sex determining region Y)-box 10	-1.39	0.38	1.62E-05	8.46E-05		6663	Hs.376984
1224	212158_at	SDC2	syndecan 2	-1.39	0.38	5.42E-06	3.37E-05		6383	Hs.1501, Hs.59
1225	213260_at	FOXC1	forkhead box C1	-1.39	0.38	4.45E-10	1.30E-08		2296	Hs.348883
1226	204731_at	TGFBR3	transforming growth factor, beta receptor	-1.39	0.38	4.43E-10	1.30E-08		7049	Hs.482390
1227	204784_s_at	MLF1	myeloid leukemia factor 1	-1.39	0.38	2.72E-06	1.87E-05		4291	Hs.85195
1228	222102_at	GSTA3	glutathione S-transferase alpha 3	-1.39	0.38	8.95E-07	7.28E-06		2940	Hs.102484
1229	213375_s_at	N4BP2L1	NEDD4 binding protein 2-like 1	-1.39	0.38	1.11E-06	8.78E-06		90634	Hs.161220
1230	205127_at	PTGS1	prostaglandin-endoperoxide synthase 1	-1.39	0.38	0.00105369	0.0031046		5742	Hs.201978
1231	213158_at			-1.4	0.38	0.00012348	0.0004832			
1232	212518_at	PIP5K1C	phosphatidylinositol-4-phosphate 5-kinas	-1.4	0.38	8.03E-06	4.67E-05		23396	Hs.282177
1233	203485_at	RTN1	reticulon 1	-1.4	0.38	0.00021359	0.000777		6252	Hs.368626
1234	201660_at	ACSL3	acyl-CoA synthetase long-chain family m	-1.4	0.38	5.52E-08	6.88E-07		2181	Hs.655772
1235	209501_at	CDR2	cerebellar degeneration-related protein 2	-1.4	0.38	6.26E-06	3.80E-05		1039	Hs.513430

	Probe	Symbol	Description	lgFCH	FCH	p	FDR		ENTREZ	UniGene
1236	219908_at	DKK2	dickkopf homolog 2 (Xenopus laevis)	-1.4	0.38	0.00130869	0.0037444		27123	Hs.211869
1237	221123_x_at	ZNF395	zinc finger protein 395	-1.4	0.38	8.72E-07	7.13E-06		55893	Hs.695998, Hs.
1238	220065_at	TNMD	tenomodulin	-1.4	0.38	0.00367028	0.0091342		64102	Hs.132957
1239	211282_x_at	TNFRSF25	tumor necrosis factor receptor superfam	-1.4	0.38	3.00E-08	4.20E-07		8718	Hs.462529
1240	213058_at	TTC28	tetratricopeptide repeat domain 28	-1.41	0.38	5.63E-07	4.90E-06		23331	Hs.387856, Hs.
1241	219833_s_at	EFHC1	EF-hand domain (C-terminal) containing	-1.41	0.38	0.00022443	0.0008101		114327	Hs.403171
1242	204018_x_at			-1.41	0.38	0.02374118	0.0459863			
1243	204284_at	PPP1R3C	protein phosphatase 1, regulatory (inhibi	-1.41	0.38	8.77E-06	5.03E-05		5507	Hs.303090
1244	212964_at	HIC2	hypermethylated in cancer 2	-1.41	0.38	7.85E-13	8.35E-11		23119	Hs.632767
1245	205857_at	SLC18A2	solute carrier family 18 (vesicular monoa	-1.41	0.38	3.75E-08	5.04E-07		6571	Hs.654476
1246	205382_s_at	CFD	complement factor D (adipsin)	-1.41	0.38	1.44E-07	1.52E-06		1675	Hs.155597
1247	208760_at	UBE2I	ubiquitin-conjugating enzyme E2I (UBC9	-1.42	0.37	1.43E-09	3.35E-08		7329	Hs.302903
1248	202262_x_at	DDAH2	dimethylarginine dimethylaminohydrolas	-1.42	0.37	1.71E-10	5.96E-09		23564	Hs.247362
1249	215235_at	SPTAN1	spectrin, alpha, non-erythrocytic 1 (alpha	-1.42	0.37	2.43E-11	1.21E-09		6709	Hs.372331
1250	219317_at	POLI	polymerase (DNA directed) iota	-1.42	0.37	9.21E-08	1.04E-06		11201	Hs.438533
1251	201540_at	FHL1	four and a half LIM domains 1	-1.42	0.37	6.65E-08	7.99E-07		2273	Hs.435369
1252	212713_at	MFAP4	microfibrillar-associated protein 4	-1.43	0.37	0.00140063	0.0039591		4239	Hs.296049
1253	212762_s_at	TCF7L2	transcription factor 7-like 2 (T-cell specifi	-1.43	0.37	5.85E-08	7.24E-07		6934	Hs.593995
1254	220615_s_at	FAR2	fatty acyl CoA reductase 2	-1.43	0.37	0.02302306	0.0447926		55711	Hs.719237
1255	209651_at	TGFB1I1	transforming growth factor beta 1 induce	-1.43	0.37	1.06E-09	2.64E-08		7041	Hs.513530
1256	214850_at	LOC100170939	glucuronidase, beta pseudogene	-1.43	0.37	3.11E-06	2.10E-05		1E+08	Hs.654588
1257	202519_at	MLXIP	MLX interacting protein	-1.43	0.37	3.82E-12	2.82E-10		22877	Hs.437153
1258	209293_x_at	ID4	inhibitor of DNA binding 4, dominant neg	-1.43	0.37	9.21E-08	1.04E-06		3400	Hs.519601
1259	212310_at	MIA3	melanoma inhibitory activity family, mem	-1.43	0.37	8.44E-09	1.47E-07		375056	Hs.118474
1260	213489_at	MAPRE2	microtubule-associated protein, RP/EB f	-1.44	0.37	1.65E-08	2.55E-07		10982	Hs.532824
1261	212427_at	KIAA0368	KIAA0368	-1.44	0.37	2.49E-08	3.61E-07		23392	Hs.368255
1262	219127_at	ATAD4	ATPase family, AAA domain containing 4	-1.44	0.37	3.72E-07	3.43E-06		79170	Hs.368260
1263	201952_at	ALCAM	activated leukocyte cell adhesion molecu	-1.44	0.37	6.92E-14	1.59E-11		214	Hs.591293
1264	201787_at	FBLN1	fibulin 1	-1.44	0.37	2.98E-09	6.16E-08		2192	Hs.24601, Hs.5
1265	201996_s_at	SPEN	spen homolog, transcriptional regulator (	-1.44	0.37	0.00638877	0.014779		23013	Hs.558463
1266	205380_at	PDZK1	PDZ domain containing 1	-1.44	0.37	0.00171288	0.0047072		5174	Hs.444751
1267	221272_s_at	C1orf21	chromosome 1 open reading frame 21	-1.45	0.37	2.12E-08	3.15E-07		81563	Hs.497159, Hs.
1268	208763_s_at	TSC22D3	TSC22 domain family, member 3	-1.45	0.37	2.96E-10	9.54E-09		1831	Hs.716410
1269	219440_at	RAI2	retinoic acid induced 2	-1.45	0.37	4.77E-08	6.14E-07		10742	Hs.446680
1270	211355_x_at	LEPR	leptin receptor	-1.45	0.37	0.00286982	0.0073629		3953	Hs.705413
1271	221834_at			-1.45	0.37	2.38E-10	7.92E-09			
1272	212510_at	GPD1L	glycerol-3-phosphate dehydrogenase 1-l	-1.45	0.37	1.08E-09	2.68E-08		23171	Hs.82432
1273	205932_s_at	MSX1	msh homeobox 1	-1.45	0.37	7.40E-07	6.20E-06		4487	Hs.424414
1274	221264_s_at	TARDBP	TAR DNA binding protein	-1.45	0.37	4.61E-07	4.12E-06		23435	Hs.300624, Hs.
1275	219188_s_at	MACROD1	MACRO domain containing 1	-1.45	0.37	2.10E-10	7.11E-09		28992	Hs.602898
1276	204919_at	PRR4	proline rich 4 (lacrimal)	-1.45	0.37	0.00095769	0.0028538		11272	Hs.408153
1277	209298_s_at	ITSN1	intersectin 1 (SH3 domain protein)	-1.45	0.37	1.66E-06	1.23E-05		6453	Hs.160324
1278	203919_at	TCEA2	transcription elongation factor A (SII), 2	-1.45	0.37	3.18E-07	2.99E-06		6919	Hs.505004, Hs.
1279	203706_s_at	FZD7	frizzled homolog 7 (Drosophila)	-1.46	0.36	7.23E-08	8.49E-07		8324	Hs.173859
1280	215358_x_at	ZNF37B	zinc finger protein 37B (pseudogene)	-1.46	0.36	1.25E-07	1.35E-06		1E+08	Hs.646695, Hs.
1281	205003_at	DOCK4	dedicator of cytokinesis 4	-1.46	0.36	2.15E-05	0.0001076		9732	Hs.654652
1282	206093_x_at			-1.46	0.36	1.02E-07	1.14E-06			
1283	202218_s_at	FADS2	fatty acid desaturase 2	-1.46	0.36	0.00434842	0.0105863		9415	Hs.502745
1284	202994_s_at	FBLN1	fibulin 1	-1.46	0.36	5.91E-09	1.09E-07		2192	Hs.24601, Hs.5
1285	204997_at	GPD1	glycerol-3-phosphate dehydrogenase 1 (	-1.47	0.36	0.00029443	0.0010217		2819	Hs.524418
1286	212503_s_at	DIP2C	DIP2 disco-interacting protein 2 homolog	-1.47	0.36	9.04E-08	1.03E-06		22982	Hs.432397
1287	208383_s_at	PCK1	phosphoenolpyruvate carboxykinase 1 (s	-1.47	0.36	0.00238693	0.006269		5105	Hs.1872
1288	202661_at	ITPR2	inositol 1,4,5-triphosphate receptor, type	-1.47	0.36	2.60E-09	5.55E-08		3709	Hs.512235
1289	201951_at	ALCAM	activated leukocyte cell adhesion molecu	-1.47	0.36	6.21E-14	1.48E-11		214	Hs.591293
1290	205547_s_at	TAGLN	transgelin	-1.48	0.36	2.68E-05	0.000131		6876	Hs.410977
1291	213675_at			-1.48	0.36	3.85E-09	7.55E-08			
1292	208741_at	SAP18	Sin3A-associated protein, 18kDa	-1.48	0.36	2.94E-10	9.53E-09		10284	Hs.524899
1293	209183_s_at	C10orf10	chromosome 10 open reading frame 10	-1.49	0.36	1.92E-07	1.95E-06		11067	Hs.93675
1294	220940_at	ANKRD36B	ankyrin repeat domain 36B	-1.49	0.36	0.0001766	0.000661		57730	Hs.532921
1295	221019_s_at	COLEC12	collectin sub-family member 12	-1.49	0.36	0.00030917	0.0010628		81035	Hs.464422
1296	208869_s_at	GABARAPL1	GABA(A) receptor-associated protein lik	-1.49	0.36	6.26E-11	2.64E-09		23710	Hs.524250
1297	210198_s_at	PLP1	proteolipid protein 1	-1.49	0.36	5.08E-06	3.20E-05		5354	Hs.1787
1298	204154_at	CDO1	cysteine dioxygenase, type I	-1.49	0.36	3.15E-05	0.0001502		1036	Hs.442378
1299	209621_s_at	PDLIM3	PDZ and LIM domain 3	-1.49	0.36	0.00023642	0.0008462		27295	Hs.85862
1300	218510_x_at	FAM134B	family with sequence similarity 134, men	-1.49	0.36	8.94E-13	9.28E-11		54463	Hs.481704

	Probe	Symbol	Description	lgFCH	FCH	p	FDR		ENTREZ	UniGene
1301	220038_at			-1.5	0.35	9.26E-11	3.68E-09			
1302	207283_at	RPL23AP32	ribosomal protein L23a pseudogene 32	-1.5	0.35	4.74E-08	6.11E-07		56969	Hs.657366
1303	201150_s_at	TIMP3	TIMP metallopeptidase inhibitor 3	-1.5	0.35	1.91E-09	4.28E-08		7078	Hs.644633, Hs.
1304	222362_at	AGFG2	ArfGAP with FG repeats 2	-1.5	0.35	1.18E-07	1.29E-06		3268	Hs.521083
1305	201149_s_at	TIMP3	TIMP metallopeptidase inhibitor 3	-1.5	0.35	0.00034074	0.0011544		7078	Hs.644633, Hs.
1306	211958_at	IGFBP5	insulin-like growth factor binding protein	-1.5	0.35	0.00076132	0.0023301		3488	Hs.607212
1307	202363_at	SPOCK1	sparc/osteonectin, cwcv and kazal-like d	-1.5	0.35	0.00013032	0.0005079		6695	Hs.643338
1308	218418_s_at	KANK2	KN motif and ankyrin repeat domains 2	-1.5	0.35	1.81E-07	1.85E-06		25959	Hs.284208, Hs.
1309	208131_s_at	PTGIS	prostaglandin I2 (prostacyclin) synthase	-1.5	0.35	3.32E-07	3.12E-06		5740	Hs.302085
1310	213924_at	MPPE1	metallophosphoesterase 1	-1.51	0.35	5.65E-07	4.91E-06		65258	Hs.712666
1311	201116_s_at	CPE	carboxypeptidase E	-1.51	0.35	0.00044109	0.0014452		1363	Hs.75360
1312	204985_s_at	TRAPPC6A	trafficking protein particle complex 6A	-1.51	0.35	1.16E-10	4.34E-09		79090	Hs.466929
1313	205258_at	INHBB	inhibin, beta B	-1.51	0.35	3.32E-12	2.56E-10		3625	Hs.1735
1314	205794_s_at	NOVA1	neuro-oncological ventral antigen 1	-1.51	0.35	2.58E-07	2.49E-06		4857	Hs.31588
1315	209016_s_at	KRT7	keratin 7	-1.51	0.35	7.03E-05	0.0002978		3855	Hs.411501, Hs.
1316	211356_x_at	LEPR	leptin receptor	-1.52	0.35	0.00245161	0.006427		3953	Hs.705413
1317	213611_at	AQP5	aquaporin 5	-1.52	0.35	5.51E-05	0.0002415		362	Hs.298023
1318	204777_s_at	MAL	mal, T-cell differentiation protein	-1.52	0.35	2.83E-09	5.91E-08		4118	Hs.80395
1319	219429_at	FA2H	fatty acid 2-hydroxylase	-1.52	0.35	0.0003621	0.0012159		79152	Hs.461329
1320	201737_s_at	6-Mar	membrane-associated ring finger (C3HC	-1.52	0.35	1.46E-06	1.11E-05		10299	Hs.432862
1321	203680_at	PRKAR2B	protein kinase, cAMP-dependent, regula	-1.52	0.35	4.08E-05	0.0001867		5577	Hs.433068
1322	214175_x_at	PDLIM4	PDZ and LIM domain 4	-1.53	0.35	1.31E-16	1.54E-13		8572	Hs.424312
1323	205200_at	CLEC3B	C-type lectin domain family 3, member B	-1.53	0.35	4.32E-07	3.89E-06		7123	Hs.476092
1324	202436_s_at	CYP1B1	cytochrome P450, family 1, subfamily B,	-1.54	0.35	0.00011075	0.0004401		1545	Hs.154654, Hs.
1325	214761_at	ZNF423	zinc finger protein 423	-1.54	0.34	6.35E-07	5.47E-06		23090	Hs.530930
1326	204754_at	HLF	hepatic leukemia factor	-1.54	0.34	1.94E-08	2.92E-07		3131	Hs.196952
1327	213413_at			-1.54	0.34	0.00010292	0.000413			
1328	214102_at	ARAP2	ArfGAP with RhoGAP domain, ankyrin re	-1.54	0.34	4.92E-07	4.35E-06		116984	Hs.479451
1329	213568_at	OSR2	odd-skipped related 2 (Drosophila)	-1.54	0.34	2.33E-07	2.28E-06		116039	Hs.253247
1330	216333_x_at			-1.54	0.34	3.08E-08	4.29E-07			
1331	203382_s_at	APOE	apolipoprotein E	-1.55	0.34	1.18E-10	4.40E-09		348	Hs.654439
1332	212233_at	MAP1B	microtubule-associated protein 1B	-1.55	0.34	6.95E-10	1.89E-08		4131	Hs.335079
1333	213686_at			-1.55	0.34	1.04E-08	1.76E-07			
1334	213380_x_at	MSTP9	macrophage stimulating, pseudogene 9	-1.55	0.34	5.28E-07	4.62E-06		11223	Hs.349110, Hs.
1335	220276_at	RERGL	RERG/RAS-like	-1.56	0.34	4.04E-05	0.000185		79785	Hs.115497
1336	206898_at	CDH19	cadherin 19, type 2	-1.56	0.34	2.60E-08	3.72E-07		28513	Hs.42771
1337	203335_at	PHYH	phytanoyl-CoA 2-hydroxylase	-1.56	0.34	2.48E-09	5.30E-08		5264	Hs.498732
1338	215536_at	HLA-DQB2	major histocompatibility complex, class I	-1.56	0.34	1.71E-05	8.83E-05		3120	Hs.409934
1339	213364_s_at	SNX1	sorting nexin 1	-1.56	0.34	5.93E-13	7.07E-11		6642	Hs.188634
1340	202222_s_at	DES	desmin	-1.56	0.34	0.00010259	0.0004119		1674	Hs.594952
1341	207912_s_at			-1.56	0.34	5.07E-05	0.0002247			
1342	221645_s_at	ZNF83	zinc finger protein 83	-1.57	0.34	1.43E-11	7.95E-10		55769	Hs.467210, Hs.
1343	203881_s_at	DMD	dystrophin	-1.57	0.34	2.93E-08	4.12E-07		1756	Hs.495912
1344	210299_s_at	FHL1	four and a half LIM domains 1	-1.57	0.34	6.03E-06	3.68E-05		2273	Hs.435369
1345	219368_at	NAP1L2	nucleosome assembly protein 1-like 2	-1.57	0.34	1.26E-05	6.88E-05		4674	Hs.66180, Hs.7
1346	213451_x_at			-1.58	0.33	3.68E-08	4.97E-07			
1347	208978_at	CRIP2	cysteine-rich protein 2	-1.58	0.33	6.13E-09	1.12E-07		1397	Hs.534309
1348	205399_at	DCLK1	doublecortin-like kinase 1	-1.58	0.33	5.48E-05	0.0002403		9201	Hs.507755, Hs.
1349	204466_s_at	SNCA	synuclein, alpha (non A4 component of a	-1.59	0.33	5.99E-08	7.36E-07		6622	Hs.21374
1350	204688_at	SGCE	sarcoglycan, epsilon	-1.59	0.33	3.86E-07	3.55E-06		8910	Hs.371199
1351	209074_s_at	FAM107A	family with sequence similarity 107, men	-1.59	0.33	2.08E-06	1.49E-05		11170	Hs.506357
1352	201801_s_at	SLC29A1	solute carrier family 29 (nucleoside trans	-1.59	0.33	1.33E-05	7.18E-05		2030	Hs.25450
1353	203903_s_at	HEPH	hephaestin	-1.59	0.33	2.92E-06	1.99E-05		9843	Hs.31720
1354	204570_at	COX7A1	cytochrome c oxidase subunit VIIa polyp	-1.59	0.33	6.56E-10	1.81E-08		1346	Hs.421621
1355	204719_at	ABCA8	ATP-binding cassette, sub-family A (ABC	-1.6	0.33	5.62E-06	3.47E-05		10351	Hs.58351
1356	205478_at	PPP1R1A	protein phosphatase 1, regulatory (inhibi	-1.6	0.33	1.10E-05	6.07E-05		5502	Hs.505662
1357	209602_s_at	GATA3	GATA binding protein 3	-1.6	0.33	3.92E-10	1.19E-08		2625	Hs.524134
1358	204083_s_at	TPM2	tropomyosin 2 (beta)	-1.6	0.33	1.35E-05	7.25E-05		7169	Hs.300772
1359	204223_at	PRELP	proline/arginine-rich end leucine-rich rep	-1.6	0.33	7.47E-10	1.98E-08		5549	Hs.632481
1360	208711_s_at	CCND1	cyclin D1	-1.6	0.33	1.89E-07	1.93E-06		595	Hs.523852
1361	205498_at	GHR	growth hormone receptor	-1.6	0.33	8.90E-07	7.24E-06		2690	Hs.125180, Hs.
1362	201313_at	ENO2	enolase 2 (gamma, neuronal)	-1.6	0.33	2.18E-07	2.17E-06		2026	Hs.511915
1363	221527_s_at	PARD3	par-3 partitioning defective 3 homolog (C	-1.6	0.33	3.16E-11	1.48E-09		56288	Hs.131489
1364	214243_s_at			-1.6	0.33	1.64E-05	8.54E-05			
1365	202274_at	ACTG2	actin, gamma 2, smooth muscle, enteric	-1.6	0.33	0.00018377	0.0006832		72	Hs.516105

	Probe	Symbol	Description	lgFCH	FCH	p	FDR		ENTREZ	UniGene
1366	202555_s_at	MYLK	myosin light chain kinase	-1.6	0.33	3.03E-05	0.0001454		4638	Hs.477375
1367	212677_s_at	CEP68	centrosomal protein 68kDa	-1.6	0.33	1.51E-06	1.14E-05		23177	Hs.709257
1368	215239_x_at	ZNF273	zinc finger protein 273	-1.6	0.33	3.38E-07	3.17E-06		10793	Hs.520889
1369	212326_at	VPS13D	vacuolar protein sorting 13 homolog D (S	-1.61	0.33	3.87E-07	3.55E-06		55187	Hs.439381
1370	205438_at	PTPN21	protein tyrosine phosphatase, non-recep	-1.61	0.33	6.18E-08	7.57E-07		11099	Hs.437040
1371	209283_at	CRYAB	crystallin, alpha B	-1.61	0.33	3.59E-11	1.64E-09		1410	Hs.408767
1372	217025_s_at	DBN1	drebrin 1	-1.61	0.33	2.34E-07	2.29E-06		1627	Hs.130316
1373	208712_at	CCND1	cyclin D1	-1.62	0.33	1.01E-17	2.61E-14		595	Hs.523852
1374	221796_at	NTRK2	neurotrophic tyrosine kinase, receptor, ty	-1.62	0.33	3.31E-05	0.0001566		4915	Hs.494312, Hs.
1375	207522_s_at	ATP2A3	ATPase, Ca++ transporting, ubiquitous	-1.62	0.32	2.53E-06	1.76E-05		489	Hs.513870
1376	213030_s_at	PLXNA2	plexin A2	-1.62	0.32	2.97E-08	4.17E-07		5362	Hs.497626
1377	209603_at	GATA3	GATA binding protein 3	-1.63	0.32	1.88E-08	2.85E-07		2625	Hs.524134
1378	204442_x_at	LTBP4	latent transforming growth factor beta bi	-1.63	0.32	5.27E-11	2.29E-09		8425	Hs.466766
1379	205765_at	CYP3A5	cytochrome P450, family 3, subfamily A,	-1.63	0.32	3.33E-08	4.58E-07		1577	Hs.695915
1380	201525_at	APOD	apolipoprotein D	-1.63	0.32	1.35E-06	1.04E-05		347	Hs.522555
1381	219059_s_at	LYVE1	lymphatic vessel endothelial hyaluronan	-1.64	0.32	0.00026752	0.0009398		10894	Hs.655332, Hs.
1382	202747_s_at	ITM2A	integral membrane protein 2A	-1.64	0.32	1.76E-09	4.01E-08		9452	Hs.17109, Hs.6
1383	209581_at	PLA2G16	phospholipase A2, group XVI	-1.64	0.32	0.00030885	0.0010621		11145	Hs.502775
1384	210298_x_at	FHL1	four and a half LIM domains 1	-1.64	0.32	0.0003673	0.0012317		2273	Hs.435369
1385	214071_at	GNAL	guanine nucleotide binding protein (G pr	-1.64	0.32	7.66E-05	0.0003217		2774	Hs.136295
1386	209242_at	PEG3	paternally expressed 3	-1.64	0.32	1.87E-08	2.84E-07		5178	Hs.719209
1387	221747_at	TNS1	tensin 1	-1.64	0.32	3.05E-08	4.25E-07		7145	Hs.471381
1388	221234_s_at	BACH2	BTB and CNC homology 1, basic leucine	-1.64	0.32	2.86E-09	5.94E-08		60468	Hs.269764, Hs.
1389	212494_at	TENC1	tensin like C1 domain containing phosph	-1.65	0.32	1.21E-09	2.94E-08		23371	Hs.343334, Hs.
1390	218532_s_at	FAM134B	family with sequence similarity 134, mem	-1.65	0.32	9.38E-11	3.71E-09		54463	Hs.481704
1391	209616_s_at	CES1	carboxylesterase 1 (monocyte/macropha	-1.65	0.32	0.00046427	0.0015085		1066	Hs.558865
1392	213765_at	MFAP5	microfibrillar associated protein 5	-1.65	0.32	0.00057189	0.0018094		8076	Hs.512842
1393	212850_s_at	LRP4	low density lipoprotein receptor-related p	-1.66	0.32	4.82E-07	4.27E-06		4038	Hs.4930
1394	202669_s_at	EFNB2	ephrin-B2	-1.66	0.32	5.41E-05	0.000238		1948	Hs.149239
1395	219407_s_at	LAMC3	laminin, gamma 3	-1.66	0.32	6.90E-07	5.84E-06		10319	Hs.201805
1396	215695_s_at	GYG2	glycogenin 2	-1.66	0.32	0.00015823	0.0006011		8908	Hs.567381, Hs.
1397	202760_s_at	PALM2-AKAP2	PALM2-AKAP2 readthrough transcript	-1.66	0.32	1.24E-07	1.34E-06		445815	Hs.591908
1398	209655_s_at	TMEM47	transmembrane protein 47	-1.67	0.32	9.61E-06	5.41E-05		83604	Hs.8769
1399	214724_at	DIXDC1	DIX domain containing 1	-1.67	0.31	2.01E-07	2.02E-06		85458	Hs.655626
1400	221833_at			-1.67	0.31	6.70E-07	5.71E-06			
1401	219518_s_at	ELL3	elongation factor RNA polymerase II-like	-1.67	0.31	2.31E-09	5.00E-08		80237	Hs.706346
1402	205097_at	SLC26A2	solute carrier family 26 (sulfate transport	-1.67	0.31	0.0026087	0.0067712		1836	Hs.302738
1403	214505_s_at	FHL1	four and a half LIM domains 1	-1.67	0.31	0.00024306	0.0008661		2273	Hs.435369
1404	203697_at	FRZB	frizzled-related protein	-1.67	0.31	6.87E-07	5.82E-06		2487	Hs.128453
1405	202973_x_at	FAM13A	family with sequence similarity 13, meml	-1.67	0.31	6.78E-09	1.22E-07		10144	Hs.97270
1406	211105_s_at	NFATC1	nuclear factor of activated T-cells, cytopl	-1.68	0.31	1.35E-08	2.17E-07		4772	Hs.534074, Hs.
1407	218876_at	TPPP3	tubulin polymerization-promoting protein	-1.68	0.31	0.00050254	0.0016166		51673	Hs.534458
1408	213106_at	ATP8A1	ATPase, aminophospholipid transporter	-1.69	0.31	4.74E-08	6.11E-07		10396	Hs.435052
1409	204948_s_at	FST	follistatin	-1.69	0.31	2.28E-12	1.94E-10		10468	Hs.9914
1410	217525_at	OLFML1	olfactomedin-like 1	-1.69	0.31	6.29E-05	0.0002706		283298	Hs.503500
1411	204288_s_at	SORBS2	sorbin and SH3 domain containing 2	-1.69	0.31	7.13E-10	1.91E-08		8470	Hs.619806, Hs.
1412	213075_at	OLFML2A	olfactomedin-like 2A	-1.69	0.31	1.13E-11	6.78E-10		169611	Hs.357004
1413	209763_at	CHRDL1	chordin-like 1	-1.69	0.31	6.52E-07	5.58E-06		91851	Hs.496587
1414	213122_at	TSPYL5	TSPY-like 5	-1.7	0.31	7.01E-09	1.25E-07		85453	Hs.173094
1415	201431_s_at	DPYSL3	dihydropyrimidinase-like 3	-1.7	0.31	8.23E-08	9.50E-07		1809	Hs.519659
1416	220744_s_at	IFT122	intraflagellar transport 122 homolog (Chl	-1.7	0.31	3.64E-10	1.12E-08		55764	Hs.655284
1417	212660_at	PHF15	PHD finger protein 15	-1.71	0.31	9.94E-10	2.52E-08		23338	Hs.483419
1418	58780_s_at	FLJ10357	hypothetical protein FLJ10357	-1.71	0.3	1.19E-11	6.89E-10		55701	Hs.35125
1419	202435_s_at	CYP1B1	cytochrome P450, family 1, subfamily B,	-1.71	0.3	0.00075566	0.0023156		1545	Hs.154654, Hs.
1420	221950_at	EMX2	empty spiracles homeobox 2	-1.71	0.3	1.13E-07	1.25E-06		2018	Hs.202095
1421	206101_at	ECM2	extracellular matrix protein 2, female org	-1.72	0.3	6.41E-06	3.88E-05		1842	Hs.117060
1422	218692_at	GOLSYN	Golgi-localized protein	-1.73	0.3	1.05E-10	4.02E-09		55638	Hs.390738
1423	213388_at	PDE4DIP	phosphodiesterase 4D interacting protei	-1.73	0.3	3.81E-11	1.74E-09		9659	Hs.584841, Hs.
1424	201058_s_at	MYL9	myosin, light chain 9, regulatory	-1.73	0.3	5.30E-05	0.0002337		10398	Hs.504687
1425	201843_s_at	EFEMP1	EGF-containing fibulin-like extracellular r	-1.73	0.3	3.96E-06	2.57E-05		2202	Hs.76224
1426	200962_at	RPL31	ribosomal protein L31	-1.73	0.3	3.95E-07	3.60E-06		6160	Hs.469473
1427	221249_s_at	FAM117A	family with sequence similarity 117, mem	-1.73	0.3	5.82E-12	3.92E-10		81558	Hs.514308
1428	205549_at	PCP4	Purkinje cell protein 4	-1.74	0.3	4.32E-06	2.78E-05		5121	Hs.80296
1429	208003_s_at	NFAT5	nuclear factor of activated T-cells 5, tonic	-1.74	0.3	0.00094738	0.0028289		10725	Hs.371987
1430	212980_at	USP34	ubiquitin specific peptidase 34	-1.74	0.3	9.42E-11	3.72E-09		9736	Hs.644708

	Probe	Symbol	Description	lgFCH	FCH	p	FDR		ENTREZ	UniGene
1431	218974_at	SOBP	sine oculis binding protein homolog (Dro	-1.74	0.3	2.64E-08	3.77E-07		55084	Hs.445244
1432	201147_s_at	TIMP3	TIMP metallopeptidase inhibitor 3	-1.74	0.3	2.28E-07	2.24E-06		7078	Hs.644633, Hs.
1433	208964_s_at	FADS1	fatty acid desaturase 1	-1.74	0.3	6.38E-05	0.0002744		3992	Hs.503546
1434	209897_s_at	SLIT2	slit homolog 2 (Drosophila)	-1.74	0.3	1.68E-05	8.71E-05		9353	Hs.29802
1435	206726_at	HPGDS	hematopoietic prostaglandin D synthase	-1.74	0.3	1.46E-06	1.10E-05		27306	Hs.128433
1436	201148_s_at	TIMP3	TIMP metallopeptidase inhibitor 3	-1.75	0.3	3.57E-07	3.31E-06		7078	Hs.644633, Hs.
1437	206030_at	ASPA	aspartoacylase (Canavan disease)	-1.75	0.3	6.85E-10	1.87E-08		443	Hs.171142
1438	204938_s_at	PLN	phospholamban	-1.75	0.3	0.00101683	0.0030112		5350	Hs.170839
1439	209869_at	ADRA2A	adrenergic, alpha-2A-, receptor	-1.75	0.3	5.11E-06	3.21E-05		150	Hs.249159
1440	214475_x_at	CAPN3	calpain 3, (p94)	-1.76	0.3	2.19E-07	2.18E-06		825	Hs.143261
1441	205440_s_at	NPY1R	neuropeptide Y receptor Y1	-1.76	0.3	3.73E-09	7.39E-08		4886	Hs.519057
1442	213156_at			-1.76	0.3	0.00018933	0.0006999			
1443	203951_at	CNN1	calponin 1, basic, smooth muscle	-1.76	0.3	1.79E-05	9.21E-05		1264	Hs.465929
1444	204940_at	PLN	phospholamban	-1.76	0.3	1.91E-05	9.74E-05		5350	Hs.170839
1445	212793_at	DAAM2	dishevelled associated activator of morp	-1.76	0.29	1.02E-07	1.14E-06		23500	Hs.357128
1446	205907_s_at	OMD	osteomodulin	-1.76	0.29	3.59E-05	0.0001674		4958	Hs.94070
1447	205259_at	NR3C2	nuclear receptor subfamily 3, group C, m	-1.77	0.29	1.63E-11	8.82E-10		4306	Hs.163924
1448	222357_at	ZBTB20	zinc finger and BTB domain containing 2	-1.77	0.29	2.22E-06	1.57E-05		26137	Hs.655108
1449	36829_at	PER1	period homolog 1 (Drosophila)	-1.77	0.29	1.73E-06	1.27E-05		5187	Hs.445534
1450	214451_at	TFAP2B	transcription factor AP-2 beta (activating	-1.78	0.29	4.43E-08	5.78E-07		7021	Hs.33102
1451	221988_at	C19orf42	chromosome 19 open reading frame 42	-1.78	0.29	9.32E-12	5.79E-10		79086	Hs.356467
1452	216614_at			-1.78	0.29	3.34E-06	2.22E-05			
1453	206465_at	ACSBG1	acyl-CoA synthetase bubblegum family r	-1.78	0.29	0.00194624	0.0052587		23205	Hs.655760
1454	214375_at			-1.78	0.29	3.52E-05	0.0001646			
1455	213397_x_at			-1.78	0.29	2.36E-09	5.09E-08			
1456	205141_at	ANG	angiogenin, ribonuclease, RNase A fami	-1.79	0.29	1.74E-11	9.24E-10		283	Hs.283749
1457	202992_at	C7	complement component 7	-1.79	0.29	1.08E-06	8.56E-06		730	Hs.78065
1458	213222_at	PLCB1	phospholipase C, beta 1 (phosphoinositi	-1.79	0.29	1.57E-07	1.65E-06		23236	Hs.431173
1459	205392_s_at			-1.79	0.29	3.02E-06	2.05E-05			
1460	40837_at	TLE2	transducin-like enhancer of split 2 (E(sp1	-1.79	0.29	1.23E-11	7.02E-10		7089	Hs.332173
1461	209447_at	SYNE1	spectrin repeat containing, nuclear enve	-1.8	0.29	1.90E-11	9.93E-10		23345	Hs.12967
1462	209543_s_at	CD34	CD34 molecule	-1.8	0.29	3.56E-08	4.83E-07		947	Hs.374990
1463	204160_s_at	ENPP4	ectonucleotide pyrophosphatase/phosph	-1.8	0.29	1.36E-07	1.45E-06		22875	Hs.643497
1464	214433_s_at	SELENBP1	selenium binding protein 1	-1.8	0.29	7.18E-10	1.92E-08		8991	Hs.632460
1465	201497_x_at	MYH11	myosin, heavy chain 11, smooth muscle	-1.8	0.29	3.16E-06	2.12E-05		4629	Hs.460109
1466	218517_at	PHF17	PHD finger protein 17	-1.81	0.29	3.94E-13	5.07E-11		79960	Hs.12420
1467	219229_at	SLCO3A1	solute carrier organic anion transporter f	-1.81	0.29	7.62E-11	3.07E-09		28232	Hs.311187
1468	201539_s_at	FHL1	four and a half LIM domains 1	-1.81	0.29	9.00E-05	0.000369		2273	Hs.435369
1469	206378_at	SCGB2A2	secretoglobin, family 2A, member 2	-1.81	0.28	0.00087552	0.0026352		4250	Hs.46452
1470	207092_at	LEP	leptin	-1.81	0.28	0.00943763	0.0207177		3952	Hs.194236
1471	201496_x_at	MYH11	myosin, heavy chain 11, smooth muscle	-1.81	0.28	4.14E-05	0.0001893		4629	Hs.460109
1472	211841_s_at	TNFRSF25	tumor necrosis factor receptor superfam	-1.82	0.28	4.94E-08	6.34E-07		8718	Hs.462529
1473	220559_at	EN1	engrailed homeobox 1	-1.83	0.28	6.59E-09	1.19E-07		2019	Hs.271977
1474	205226_at	PDGFRL	platelet-derived growth factor receptor-lil	-1.83	0.28	8.44E-05	0.0003507		5157	Hs.458573
1475	218718_at	PDGFC	platelet derived growth factor C	-1.84	0.28	1.17E-09	2.85E-08		56034	Hs.570855
1476	209815_at	PTCH1	patched homolog 1 (Drosophila)	-1.84	0.28	3.51E-12	2.67E-10		5727	Hs.494538, Hs.
1477	202746_at	ITM2A	integral membrane protein 2A	-1.84	0.28	1.51E-09	3.50E-08		9452	Hs.17109, Hs.6
1478	212157_at	SDC2	syndecan 2	-1.84	0.28	1.91E-08	2.88E-07		6383	Hs.1501, Hs.59
1479	205568_at	AQP9	aquaporin 9	-1.85	0.28	3.18E-08	4.40E-07		366	Hs.104624
1480	202806_at	DBN1	drebrin 1	-1.85	0.28	9.77E-09	1.67E-07		1627	Hs.130316
1481	206227_at	CILP	cartilage intermediate layer protein, nucl	-1.85	0.28	0.00359342	0.0089706		8483	Hs.442180
1482	205073_at	CYP2J2	cytochrome P450, family 2, subfamily J,	-1.85	0.28	1.11E-08	1.85E-07		1573	Hs.152096
1483	218546_at	C1orf115	chromosome 1 open reading frame 115	-1.86	0.28	3.06E-09	6.28E-08		79762	Hs.519839
1484	202409_at			-1.86	0.28	6.66E-06	4.00E-05			
1485	214734_at	EXPH5	exophilin 5	-1.86	0.27	0.00017883	0.0006676		23086	Hs.28540
1486	219295_s_at	PCOLCE2	procollagen C-endopeptidase enhancer	-1.87	0.27	0.00104958	0.0030933		26577	Hs.8944
1487	221276_s_at	SYNC	syncoilin, intermediate filament protein	-1.87	0.27	1.84E-13	2.93E-11		81493	Hs.712631
1488	203240_at			-1.87	0.27	3.01E-07	2.86E-06			
1489	222368_at			-1.87	0.27	5.09E-08	6.49E-07			
1490	206201_s_at	MEOX2	mesenchyme homeobox 2	-1.87	0.27	2.96E-07	2.82E-06		4223	Hs.170355
1491	205908_s_at	OMD	osteomodulin	-1.87	0.27	1.70E-05	8.79E-05		4958	Hs.94070
1492	217047_s_at	FAM13A	family with sequence similarity 13, meml	-1.87	0.27	1.31E-08	2.11E-07		10144	Hs.97270
1493	203685_at	BCL2	B-cell CLL/lymphoma 2	-1.88	0.27	5.65E-14	1.40E-11		596	Hs.150749
1494	219909_at	MMP28	matrix metallopeptidase 28	-1.88	0.27	2.24E-13	3.27E-11		79148	Hs.380710
1495	209291_at	ID4	inhibitor of DNA binding 4, dominant neg	-1.88	0.27	6.46E-14	1.51E-11		3400	Hs.519601

	Probe	Symbol	Description	lgFCH	FCH	p	FDR		ENTREZ	UniGene
1496	203146_s_at	GABBR1	gamma-aminobutyric acid (GABA) B rec	-1.89	0.27	1.89E-06	1.37E-05		2550	Hs.167017
1497	213764_s_at	MFAP5	microfibrillar associated protein 5	-1.89	0.27	2.37E-05	0.0001177		8076	Hs.512842
1498	205803_s_at	TRPC1	transient receptor potential cation chann	-1.89	0.27	4.11E-07	3.72E-06		7220	Hs.250687
1499	203296_s_at	ATP1A2	ATPase, Na+/K+ transporting, alpha 2 (+	-1.9	0.27	1.86E-09	4.20E-08		477	Hs.34114
1500	217897_at	FXYD6	FXYD domain containing ion transport re	-1.9	0.27	4.59E-10	1.34E-08		53826	Hs.713034
1501	208609_s_at	TNXB	tenascin XB	-1.9	0.27	2.21E-07	2.19E-06		7148	Hs.42853, Hs.4
1502	209656_s_at	TMEM47	transmembrane protein 47	-1.91	0.27	4.82E-07	4.27E-06		83604	Hs.8769
1503	209343_at	EFHD1	EF-hand domain family, member D1	-1.91	0.27	2.46E-09	5.27E-08		80303	Hs.516769, Hs.
1504	203980_at	FABP4	fatty acid binding protein 4, adipocyte	-1.93	0.26	5.56E-05	0.0002431		2167	Hs.391561
1505	218966_at	MYO5C	myosin VC	-1.93	0.26	5.39E-08	6.75E-07		55930	Hs.487036
1506	206167_s_at	ARHGAP6	Rho GTPase activating protein 6	-1.93	0.26	6.03E-07	5.21E-06		395	Hs.435291
1507	206873_at	CA6	carbonic anhydrase VI	-1.93	0.26	1.83E-07	1.87E-06		765	Hs.100322
1508	212805_at	PRUNE2	prune homolog 2 (Drosophila)	-1.94	0.26	2.97E-09	6.14E-08		158471	Hs.262857
1509	205529_s_at	RUNX1T1	runt-related transcription factor 1; translo	-1.94	0.26	1.06E-05	5.87E-05		862	Hs.368431
1510	209685_s_at	PRKCB	protein kinase C, beta	-1.96	0.26	6.96E-11	2.85E-09		5579	Hs.460355
1511	212845_at	SAMD4A	sterile alpha motif domain containing 4A	-1.96	0.26	5.97E-08	7.34E-07		23034	Hs.98259
1512	208962_s_at	FADS1	fatty acid desaturase 1	-1.96	0.26	0.00078931	0.0024032		3992	Hs.503546
1513	213234_at	KIAA1467	KIAA1467	-1.96	0.26	5.88E-14	1.43E-11		57613	Hs.132660
1514	214027_x_at			-1.97	0.26	5.62E-05	0.0002453			
1515	203851_at	IGFBP6	insulin-like growth factor binding protein	-1.97	0.26	6.48E-08	7.84E-07		3489	Hs.274313
1516	208096_s_at	COL21A1	collagen, type XXI, alpha 1	-1.97	0.25	2.25E-07	2.22E-06		81578	Hs.47629
1517	217284_x_at	SERHL2	serine hydrolase-like 2	-1.98	0.25	4.20E-05	0.0001919		253190	Hs.360940, Hs.
1518	204294_at	AMT	aminomethyltransferase	-1.98	0.25	3.78E-09	7.48E-08		275	Hs.102
1519	219689_at	SEMA3G	sema domain, immunoglobulin domain (	-1.98	0.25	2.07E-08	3.09E-07		56920	Hs.59729
1520	210078_s_at	KCNAB1	potassium voltage-gated channel, shake	-1.98	0.25	3.54E-08	4.81E-07		7881	Hs.654519, Hs.
1521	219087_at	ASPN	asporin	-1.98	0.25	6.62E-05	0.0002832		54829	Hs.435655
1522	215116_s_at	DNM1	dynamin 1	-1.98	0.25	1.09E-06	8.64E-06		1759	Hs.522413
1523	214434_at	HSPA12A	heat shock 70kDa protein 12A	-1.99	0.25	1.54E-12	1.41E-10		259217	Hs.654682
1524	201839_s_at	EPCAM	epithelial cell adhesion molecule	-2	0.25	2.55E-10	8.39E-09		4072	Hs.542050
1525	206243_at	TIMP4	TIMP metallopeptidase inhibitor 4	-2	0.25	2.10E-05	0.0001054		7079	Hs.591665
1526	212730_at	SYNM	synemin, intermediate filament protein	-2	0.25	9.20E-08	1.04E-06		23336	Hs.207106
1527	202908_at	WFS1	Wolfram syndrome 1 (wolframin)	-2.01	0.25	1.96E-14	6.17E-12		7466	Hs.518602, Hs.
1528	208963_x_at	FADS1	fatty acid desaturase 1	-2.01	0.25	0.00011199	0.0004442		3992	Hs.503546
1529	202861_at	PER1	period homolog 1 (Drosophila)	-2.01	0.25	2.31E-06	1.64E-05		5187	Hs.445534
1530	214927_at	ITGBL1	integrin, beta-like 1 (with EGF-like repea	-2.02	0.25	1.24E-05	6.79E-05		9358	Hs.696554
1531	217506_at	LOC339290	hypothetical LOC339290	-2.02	0.25	1.50E-09	3.50E-08		339290	Hs.643553, Hs.
1532	213706_at	GPD1	glycerol-3-phosphate dehydrogenase 1 (	-2.03	0.25	2.81E-05	0.0001364		2819	Hs.524418
1533	204607_at	HMGCS2	3-hydroxy-3-methylglutaryl-Coenzyme A	-2.03	0.24	1.73E-07	1.79E-06		3158	Hs.59889
1534	219895_at	FAM70A	family with sequence similarity 70, meml	-2.03	0.24	3.31E-10	1.04E-08		55026	Hs.437563
1535	211564_s_at	PDLIM4	PDZ and LIM domain 4	-2.05	0.24	5.48E-12	3.71E-10		8572	Hs.424312
1536	218087_s_at	SORBS1	sorbin and SH3 domain containing 1	-2.05	0.24	1.24E-07	1.35E-06		10580	Hs.719081
1537	208498_s_at			-2.05	0.24	4.79E-12	3.35E-10			
1538	210096_at	CYP4B1	cytochrome P450, family 4, subfamily B,	-2.06	0.24	6.91E-05	0.0002935		1580	Hs.436317
1539	218309_at	CAMK2N1	calcium/calmodulin-dependent protein ki	-2.06	0.24	5.36E-12	3.68E-10		55450	Hs.197922
1540	214823_at	ZNF204	zinc finger protein 204 pseudogene	-2.07	0.24	6.08E-13	7.10E-11		7754	Hs.8198
1541	218625_at	NRN1	neuritin 1	-2.07	0.24	8.94E-09	1.54E-07		51299	Hs.103291
1542	202437_s_at	CYP1B1	cytochrome P450, family 1, subfamily B,	-2.07	0.24	3.03E-05	0.0001454		1545	Hs.154654, Hs.
1543	201124_at	ITGB5	integrin, beta 5	-2.08	0.24	2.87E-08	4.05E-07		3693	Hs.536663
1544	49452_at	ACACB	acetyl-Coenzyme A carboxylase beta	-2.08	0.24	4.19E-09	8.06E-08		32	Hs.234898
1545	217276_x_at	SERHL2	serine hydrolase-like 2	-2.08	0.24	1.72E-05	8.91E-05		253190	Hs.360940, Hs.
1546	204519_s_at	PLLP	plasma membrane proteolipid (plasmolip	-2.09	0.24	1.37E-11	7.66E-10		51090	Hs.632215
1547	216620_s_at	ARHGEF10	Rho guanine nucleotide exchange factor	-2.09	0.24	6.00E-13	7.09E-11		9639	Hs.98594
1548	213217_at	ADCY2	adenylate cyclase 2 (brain)	-2.09	0.23	1.20E-09	2.92E-08		108	Hs.481545
1549	205081_at	CRIP1	cysteine-rich protein 1 (intestinal)	-2.1	0.23	1.59E-13	2.59E-11		1396	Hs.70327
1550	221795_at	NTRK2	neurotrophic tyrosine kinase, receptor, ty	-2.11	0.23	3.25E-05	0.0001542		4915	Hs.494312, Hs.
1551	205979_at	SCGB2A1	secretoglobin, family 2A, member 1	-2.12	0.23	9.14E-05	0.0003735		4246	Hs.97644
1552	205158_at	RNASE4	ribonuclease, RNase A family, 4	-2.13	0.23	1.01E-07	1.13E-06		6038	Hs.283749
1553	206677_at	KRT31	keratin 31	-2.15	0.23	0.00757876	0.0171341		3881	Hs.41696
1554	207430_s_at	MSMB	microseminoprotein, beta-	-2.15	0.23	2.40E-05	0.0001189		4477	Hs.255462
1555	207191_s_at	ISLR	immunoglobulin superfamily containing l	-2.15	0.23	1.22E-08	1.99E-07		3671	Hs.699822, Hs.
1556	204963_at	SSPN	sarcospan (Kras oncogene-associated g	-2.16	0.22	1.84E-10	6.32E-09		8082	Hs.183428
1557	219304_s_at	PDGFD	platelet derived growth factor D	-2.17	0.22	5.28E-05	0.0002331		80310	Hs.352298
1558	221646_s_at	ZDHHC11	zinc finger, DHHC-type containing 11	-2.19	0.22	7.57E-09	1.34E-07		79844	Hs.659832
1559	214203_s_at	PRODH	proline dehydrogenase (oxidase) 1	-2.19	0.22	2.12E-09	4.67E-08		5625	Hs.517352
1560	203498_at	RCAN2	regulator of calcineurin 2	-2.2	0.22	3.08E-09	6.29E-08		10231	Hs.440168

	Probe	Symbol	Description	lgFCH	FCH	p	FDR		ENTREZ	UniGene
1561	202975_s_at	RHOBTB3	Rho-related BTB domain containing 3	-2.21	0.22	1.26E-09	3.02E-08		22836	Hs.445030
1562	219195_at	PPARGC1A	peroxisome proliferator-activated recepto	-2.22	0.21	5.43E-12	3.70E-10		10891	Hs.527078
1563	207175_at	ADIPOQ	adiponectin, C1Q and collagen domain c	-2.25	0.21	0.00297599	0.0076019		9370	Hs.80485
1564	203523_at	LSP1	lymphocyte-specific protein 1	-2.25	0.21	3.60E-10	1.12E-08		4046	Hs.56729
1565	202052_s_at	RAI14	retinoic acid induced 14	-2.27	0.21	2.35E-12	1.96E-10		26064	Hs.431400
1566	217059_at	MUC7	mucin 7, secreted	-2.27	0.21	2.19E-05	0.0001096		4589	Hs.631946
1567	205044_at	GABRP	gamma-aminobutyric acid (GABA) A rec	-2.28	0.21	8.41E-07	6.91E-06		2568	Hs.26225
1568	216921_s_at	KRT35	keratin 35	-2.31	0.2	0.01661645	0.0339449		3886	Hs.73082
1569	201596_x_at	KRT18	keratin 18	-2.32	0.2	3.69E-08	4.97E-07		3875	Hs.406013
1570	218820_at	C14orf132	chromosome 14 open reading frame 132	-2.32	0.2	3.27E-13	4.29E-11		56967	Hs.6434
1571	204363_at	F3	coagulation factor III (thromboplastin, tis	-2.34	0.2	5.76E-17	9.27E-14		2152	Hs.62192
1572	202976_s_at	RHOBTB3	Rho-related BTB domain containing 3	-2.35	0.2	4.44E-10	1.30E-08		22836	Hs.445030
1573	210325_at	CD1A	CD1a molecule	-2.36	0.19	9.85E-09	1.68E-07		909	Hs.1309
1574	206509_at	PIP	prolactin-induced protein	-2.37	0.19	6.48E-05	0.0002783		5304	Hs.99949
1575	213800_at	CFH	complement factor H	-2.38	0.19	2.68E-09	5.70E-08		3075	Hs.363396
1576	209612_s_at	ADH1B	alcohol dehydrogenase 1B (class I), beta	-2.38	0.19	9.79E-07	7.86E-06		125	Hs.4
1577	209894_at	LEPR	leptin receptor	-2.39	0.19	1.03E-07	1.15E-06		3953	Hs.705413
1578	210571_s_at	CMAH	cytidine monophosphate-N-acetylneuran	-2.4	0.19	1.05E-08	1.78E-07		8418	Hs.484918
1579	204939_s_at	PLN	phospholamban	-2.4	0.19	8.15E-05	0.0003403		5350	Hs.170839
1580	203895_at	PLCB4	phospholipase C, beta 4	-2.41	0.19	1.05E-09	2.63E-08		5332	Hs.472101
1581	222288_at			-2.41	0.19	2.10E-06	1.50E-05			
1582	215516_at	LAMB4	laminin, beta 4	-2.42	0.19	2.71E-08	3.87E-07		22798	Hs.62022
1583	214240_at	GAL	galanin prepropeptide	-2.42	0.19	0.00788346	0.0177172		51083	Hs.278959
1584	214945_at			-2.44	0.18	7.75E-09	1.36E-07			
1585	203549_s_at	LPL	lipoprotein lipase	-2.45	0.18	0.00013485	0.0005225		4023	Hs.180878
1586	213900_at	FAM189A2	family with sequence similarity 189, mem	-2.45	0.18	7.69E-15	3.00E-12		9413	Hs.118003
1587	209094_at	DDAH1	dimethylarginine dimethylaminohydrolas	-2.46	0.18	6.65E-13	7.30E-11		23576	Hs.379858
1588	43427_at	ACACB	acetyl-Coenzyme A carboxylase beta	-2.47	0.18	2.68E-11	1.30E-09		32	Hs.234898
1589	213369_at	PCDH21	protocadherin 21	-2.48	0.18	4.73E-11	2.11E-09		92211	Hs.137556
1590	209613_s_at	ADH1B	alcohol dehydrogenase 1B (class I), beta	-2.48	0.18	4.91E-07	4.34E-06		125	Hs.4
1591	203766_s_at	LMOD1	leiomodin 1 (smooth muscle)	-2.5	0.18	1.59E-06	1.19E-05		25802	Hs.519075
1592	213880_at	LGR5	leucine-rich repeat-containing G protein-	-2.51	0.18	3.05E-05	0.0001462		8549	Hs.658889
1593	203824_at	TSPAN8	tetraspanin 8	-2.52	0.17	1.88E-09	4.22E-08		7103	Hs.170563
1594	206149_at	CHP2	calcineurin B homologous protein 2	-2.54	0.17	2.07E-11	1.07E-09		63928	Hs.178589
1595	205357_s_at	AGTR1	angiotensin II receptor, type 1	-2.58	0.17	8.15E-08	9.41E-07		185	Hs.477887, Hs.
1596	203548_s_at	LPL	lipoprotein lipase	-2.6	0.16	0.00019171	0.0007068		4023	Hs.180878
1597	219398_at	CIDEC	cell death-inducing DFFA-like effector c	-2.6	0.16	0.00037284	0.0012459		63924	Hs.567562, Hs.
1598	209292_at	ID4	inhibitor of DNA binding 4, dominant neg	-2.61	0.16	4.45E-14	1.12E-11		3400	Hs.519601
1599	213661_at	PAMR1	peptidase domain containing associated	-2.62	0.16	1.21E-14	4.10E-12		25891	Hs.55044
1600	215322_at			-2.63	0.16	1.56E-06	1.17E-05			
1601	207670_at	KRT85	keratin 85	-2.64	0.16	0.01695879	0.0345624		3891	Hs.182507
1602	204591_at	CHL1	cell adhesion molecule with homology to	-2.65	0.16	2.17E-09	4.75E-08		10752	Hs.148909
1603	221530_s_at	BHLHE41	basic helix-loop-helix family, member e4	-2.66	0.16	2.52E-13	3.60E-11		79365	Hs.177841
1604	206799_at	SCGB1D2	secretoglobin, family 1D, member 2	-2.72	0.15	0.00013982	0.0005387		10647	Hs.204096
1605	205139_s_at	UST	uronyl-2-sulfotransferase	-2.73	0.15	3.70E-09	7.36E-08		10090	Hs.657370
1606	219140_s_at	RBP4	retinol binding protein 4, plasma	-2.74	0.15	0.00045093	0.001473		5950	Hs.50223
1607	205913_at	PLIN1	perilipin 1	-2.76	0.15	0.00014357	0.000551		5346	Hs.103253
1608	220428_at	CD207	CD207 molecule, langerin	-2.78	0.15	9.07E-07	7.36E-06		50489	Hs.199731
1609	206170_at	ADRB2	adrenergic, beta-2-, receptor, surface	-2.79	0.14	5.66E-17	9.27E-14		154	Hs.591251
1610	205404_at	HSD11B1	hydroxysteroid (11-beta) dehydrogenase	-2.8	0.14	3.68E-08	4.97E-07		3290	Hs.195040
1611	218312_s_at	ZSCAN18	zinc finger and SCAN domain containing	-2.8	0.14	2.09E-13	3.13E-11		65982	Hs.235390
1612	201650_at	KRT19	keratin 19	-2.82	0.14	3.01E-08	4.20E-07		3880	Hs.654568
1613	211685_s_at	NCALD	neurocalcin delta	-2.83	0.14	2.29E-12	1.94E-10		83988	Hs.492427, Hs.
1614	207955_at	CCL27	chemokine (C-C motif) ligand 27	-2.94	0.13	2.06E-10	7.01E-09		10850	Hs.648124
1615	213050_at	COBL	cordon-bleu homolog (mouse)	-3.01	0.12	3.57E-12	2.70E-10		23242	Hs.99141
1616	210297_s_at	MSMB	microseminoprotein, beta-	-3.05	0.12	7.98E-08	9.27E-07		4477	Hs.255462
1617	221470_s_at	IL1F7	interleukin 1 family, member 7 (zeta)	-3.06	0.12	2.66E-12	2.18E-10		27178	Hs.166371
1618	205883_at	ZBTB16	zinc finger and BTB domain containing 1	-3.16	0.11	2.07E-09	4.56E-08		7704	Hs.591945, Hs.
1619	212913_at			-3.31	0.1	6.35E-20	8.17E-16			
1620	204712_at	WIF1	WNT inhibitory factor 1	-3.37	0.1	5.88E-08	7.25E-07		11197	Hs.284122
1621	214598_at	CLDN8	claudin 8	-3.44	0.09	2.14E-15	1.38E-12		9073	Hs.162209
1622	205518_s_at	CMAH	cytidine monophosphate-N-acetylneuran	-3.81	0.07	1.07E-13	2.05E-11		8418	Hs.484918

523847
715518
694721

663740

711982

710488

719474
708710

694721

593014

603116

706850

713967

712898

8114

694721

656247
591588

80976
99528
694721

716916

80976

655222
535586, Hs.591552, Hs.707804

694721

719274

05546

96221

706874

615289

684559

654498

694721

799
713967

239459, Hs.709169

696253
596972
700558, Hs.700559

8114

715518

686479

715625

713175

712555

665989

593542

99528

700558, Hs.700559

719145

713649

701035

708402

713658

713967

99528

603116

594910

05546

632420
92017

644972

91393

593014

715625

594673

703487

648635
714977

719119, Hs.7917

706850

711019

719079

603755

669931

645734

656753

718660

619408

711584

715525
689805

698060

719656

654784

659535

719680

712910

701629

618266

699209

669931

648482

604502

716660

715848

719388

679786

715525

94758

597019, Hs.707692

528006

604502

719179

712970

8247

659396

719071
1551

82719

660622

719150

715848

602267

651352

718403

718403

619338

717124

512587

602267

94758

533624, Hs.699316

93123

653608, Hs.714012

714477

512587

708867

647962

715308

656580

708807

8247

699209

719265

93123

719188

635053, Hs.716809

711562

648656, Hs.650553, Hs.684178

93123

714168

714168

719195

670221

715731

655432

655934, Hs.659798, Hs.710125

19281

717124

684631

712776

708807
94944

716609, Hs.716710
6147

701629

701518

655143

715731

613082, Hs.657186, Hs.657490, Hs.719077

714168

714168

638946
94944
8247

85104

708346

656672

703187

716556

700799

715731

656672

712776

710506

719071

635072

680132

682144
