# Supplementary material for: Evaluation of the Psoriasis Transcriptome across Different Studies by Gene Set Enrichment Analysis (GSEA)
Source: PLoS One. 2010 Apr 20;5(4):e10247. doi: 10.1371/journal.pone.0010247 (PMC2857878; doi:10.1371/journal.pone.0010247)
Supplement: Table S2 — Genes consistently identified by the four studies. (0.08 MB PDF) [file pone.0010247.s002.pdf]

Up in all 4 studies

SYMBOL	Description	Yao_lgFCH	Zou_lgFCH	Gudjonsson_lgFCH	Suarez-Farinas_lgFCH	average
ACPP	acid phosphatase, prostate	2.50	1.20	1.56	1.65	1.73
AKR1B10	aldo-keto reductase family 1, member B10 (aldose red	5.45	2.21	5.35	5.25	4.56
ALDH1A3	aldehyde dehydrogenase 1 family, member A3	2.66	1.63	1.64	2.40	2.08
ALOX12B	arachidonate 12-lipoxygenase, 12R type	2.10	1.54	1.78	1.75	1.79
APOL1	apolipoprotein L, 1	1.63	1.29	1.17	2.11	1.55
ARG1	arginase, liver	2.18	1.23	1.41	1.74	1.64
ARNTL2	aryl hydrocarbon receptor nuclear translocator-like 2	3.23	1.09	2.22	3.11	2.41
ATP12A	ATPase, H+/K+ transporting, nongastric, alpha polypep	5.35	1.18	3.01	5.71	3.81
CARHSP1	calcium regulated heat stable protein 1, 24kDa	2.77	2.11	2.02	2.47	2.34
CCNB1	cyclin B1	2.48	1.50	2.48	2.55	2.25
CCNE1	cyclin E1	2.12	14.47	1.23	1.71	4.88
CD24	CD24 molecule	3.44	2.04	2.36	3.15	2.75
CD47	CD47 molecule	2.27	1.51	1.64	1.75	1.79
CDC20	cell division cycle 20 homolog (S. cerevisiae)	1.75	14.75	1.76	2.19	5.11
CHI3L2	chitinase 3-like 2	5.12	1.65	3.82	4.27	3.72
CKS2	CDC28 protein kinase regulatory subunit 2	1.92	1.27	1.95	1.64	1.70
CLCA2	chloride channel accessory 2	1.28	1.16	1.37	1.58	1.35
CRABP2	cellular retinoic acid binding protein 2	2.31	1.66	1.81	2.24	2.00
CXCL1	chemokine (C-X-C motif) ligand 1 (melanoma growth s	2.83	2.81	2.50	3.60	2.93
CXCL10	chemokine (C-X-C motif) ligand 10	3.70	2.41	2.63	1.92	2.66
CYP7B1	cytochrome P450, family 7, subfamily B, polypeptide 1	1.42	12.23	1.10	1.03	3.94
DDX58	DEAD (Asp-Glu-Ala-Asp) box polypeptide 58	2.00	1.28	1.57	2.16	1.75
DEFB4	defensin, beta 4	7.34	1.96	7.07	5.96	5.58
DLGAP5	discs, large (Drosophila) homolog-associated protein 5	2.48	1.06	1.86	2.52	1.98
DSC2	desmocollin 2	2.07	2.33	2.58	3.27	2.56
DSG3	desmoglein 3 (pemphigus vulgaris antigen)	1.36	1.30	1.62	1.60	1.47
EHF	ets homologous factor	2.16	1.88	2.19	3.77	2.50
EIF5	eukaryotic translation initiation factor 5	1.23	1.10	1.15	1.41	1.22
ELL2	elongation factor, RNA polymerase II, 2	1.07	1.73	1.12	2.55	1.62
EPHA2	EPH receptor A2	1.41	1.24	1.28	1.62	1.38
EPHX3	epoxide hydrolase 3	1.91	1.15	1.23	1.18	1.37
EPN3	epsin 3	1.73	13.91	1.20	1.88	4.68
FANCI	Fanconi anemia, complementation group I	1.14	1.30	1.04	1.06	1.13
FCGR3B	Fc fragment of IgG, low affinity IIIb, receptor (CD16b)	1.81	1.01	1.17	1.48	1.37
FGFBP1	fibroblast growth factor binding protein 1	1.56	1.17	1.65	1.05	1.36
FOXE1	forkhead box E1 (thyroid transcription factor 2)	2.25	1.15	1.87	1.07	1.59
GBP1	guanylate binding protein 1, interferon-inducible, 67kDa	2.11	1.63	1.83	1.70	1.82
GCH1	GTP cyclohydrolase 1	1.19	1.03	1.24	1.04	1.12
GDPD3	glycerophosphodiester phosphodiesterase domain con	2.70	1.31	2.08	3.14	2.31
GGH	gamma-glutamyl hydrolase (conjugase, folylpolygamm	1.97	1.19	1.54	1.20	1.48
GK	glycerol kinase	2.16	1.19	1.96	3.39	2.17
GM2A	GM2 ganglioside activator	2.42	1.32	2.21	4.68	2.66
GZMB	granzyme B (granzyme 2, cytotoxic T-lymphocyte-asso	3.20	13.72	2.46	1.75	5.28
HAL	histidine ammonia-lyase	2.74	1.34	1.63	1.96	1.92
HERC6	hect domain and RLD 6	3.84	1.33	2.83	4.07	3.02
HIGD1A	HIG1 hypoxia inducible domain family, member 1A	1.68	1.09	1.32	1.03	1.28
HK2	hexokinase 2	1.43	1.11	1.40	1.68	1.40
HPSE	heparanase	4.60	2.23	3.75	4.43	3.75
HSPA4	heat shock 70kDa protein 4	1.42	1.07	1.07	2.13	1.42
HSPA4L	heat shock 70kDa protein 4-like	1.14	1.06	1.21	1.49	1.22
HYAL4	hyaluronoglucosaminidase 4	3.03	1.82	1.74	2.46	2.26
IFI16	interferon, gamma-inducible protein 16	1.06	1.50	1.01	1.67	1.31
IFI27	interferon, alpha-inducible protein 27	3.17	1.46	2.66	2.24	2.38
IFIT1	interferon-induced protein with tetratricopeptide repeats	2.35	1.30	1.70	2.53	1.97
IFIT3	interferon-induced protein with tetratricopeptide repeats	2.49	1.53	1.77	1.80	1.90
IL1F9	interleukin 1 family, member 9	4.83	3.48	4.60	4.37	4.32
IL4R	interleukin 4 receptor	1.74	1.07	1.17	1.31	1.32
IL8	interleukin 8	4.67	2.96	4.03	5.85	4.38

Up in all 4 studies

SYMBOL	Description	Yao_lgFCH	Zou_lgFCH	Gudjonsson_lgFCH	Suarez-Farinas_lgFCH	average
IL8RB	interleukin 8 receptor, beta	2.12	1.58	1.96	2.25	1.98
ISG15	ISG15 ubiquitin-like modifier	3.09	1.10	2.43	2.92	2.38
IVL	involucrin	2.02	1.05	1.21	1.25	1.38
KIAA0101	KIAA0101	2.21	1.30	1.89	1.72	1.78
KLK10	kallikrein-related peptidase 10	2.94	1.39	1.97	3.74	2.51
KLK13	kallikrein-related peptidase 13	3.97	2.52	3.24	4.96	3.67
KLK6	kallikrein-related peptidase 6	4.85	2.01	3.49	6.63	4.24
KRT16	keratin 16	4.57	1.99	4.11	3.68	3.59
KRT6B	keratin 6B	2.54	1.32	2.07	2.11	2.01
KYNU	kynureninase (L-kynurenine hydrolase)	5.35	2.79	4.63	6.16	4.73
LAMP3	lysosomal-associated membrane protein 3	2.57	1.17	2.19	1.97	1.97
LCN2	lipocalin 2	5.75	3.67	4.02	5.65	4.77
LDLR	low density lipoprotein receptor	1.54	1.01	1.23	1.94	1.43
LIPG	lipase, endothelial	1.20	1.31	1.44	1.63	1.39
MAD2L1	MAD2 mitotic arrest deficient-like 1 (yeast)	1.79	1.18	1.51	1.42	1.47
MMP9	matrix metallopeptidase 9 (gelatinase B, 92kDa gelatin	2.30	1.24	1.49	1.54	1.64
MPZL2	myelin protein zero-like 2	2.79	1.77	2.69	3.65	2.73
MX1	myxovirus (influenza virus) resistance 1, interferon-indu	3.24	1.45	2.32	3.23	2.56
MXD1	MAX dimerization protein 1	2.55	1.25	2.17	1.29	1.81
N4BP1	NEDD4 binding protein 1	1.41	1.05	1.06	1.66	1.30
NAMPT	nicotinamide phosphoribosyltransferase	2.36	1.69	2.54	1.82	2.10
NCAPG	non-SMC condensin I complex, subunit G	1.42	11.26	1.35	1.30	3.83
NMI	N-myc (and STAT) interactor	1.47	1.12	1.18	1.29	1.27
NUSAP1	nucleolar and spindle associated protein 1	1.20	1.12	1.22	1.66	1.30
OAS1	2',5'-oligoadenylate synthetase 1, 40/46kDa	3.22	1.26	2.55	3.70	2.68
OAS2	2'-5'-oligoadenylate synthetase 2, 69/71kDa	4.10	1.12	3.29	4.50	3.25
OAS3	2'-5'-oligoadenylate synthetase 3, 100kDa	2.40	1.02	2.06	2.99	2.12
PDZK1IP1	PDZK1 interacting protein 1	2.85	1.79	2.10	2.01	2.19
PI3	peptidase inhibitor 3, skin-derived	7.15	2.41	6.55	5.11	5.30
PLBD1	phospholipase B domain containing 1	2.22	1.30	1.66	1.81	1.75
PLSCR1	phospholipid scramblase 1	1.44	1.35	1.28	1.59	1.41
PRDM1	PR domain containing 1, with ZNF domain	2.00	1.08	1.07	2.08	1.56
PRRG4	proline rich Gla (G-carboxyglutamic acid) 4 (transmeml	1.44	10.32	1.16	1.25	3.54
PRSS3	protease, serine, 3	1.81	1.74	1.38	1.48	1.60
PTTG1	pituitary tumor-transforming 1	1.70	1.05	1.39	1.14	1.32
RRM2	ribonucleotide reductase M2	3.14	1.26	3.12	3.33	2.71
RSAD2	radical S-adenosyl methionine domain containing 2	3.40	1.51	2.56	3.48	2.74
S100A12	S100 calcium binding protein A12	6.96	2.66	5.68	7.58	5.72
S100A2	S100 calcium binding protein A2	1.60	1.22	1.46	1.27	1.39
S100A7	S100 calcium binding protein A7	3.46	1.41	3.37	1.78	2.50
S100A9	S100 calcium binding protein A9	6.11	1.67	5.18	3.90	4.22
SAMD9	sterile alpha motif domain containing 9	2.85	1.29	2.29	3.24	2.42
SERPINB1	serpin peptidase inhibitor, clade B (ovalbumin), membe	2.51	2.25	1.77	2.36	2.22
SERPINB13	serpin peptidase inhibitor, clade B (ovalbumin), membe	3.39	12.53	2.99	5.19	6.03
SERPINB3	serpin peptidase inhibitor, clade B (ovalbumin), membe	6.35	3.21	5.61	4.62	4.95
SLC23A2	solute carrier family 23 (nucleobase transporters), men	1.79	1.28	1.67	1.31	1.51
SLC5A1	solute carrier family 5 (sodium/glucose cotransporter),	3.27	14.29	1.90	2.97	5.61
SLC6A14	solute carrier family 6 (amino acid transporter), membe	4.10	2.04	3.21	3.46	3.20
SOD2	superoxide dismutase 2, mitochondrial	2.99	1.44	2.39	3.44	2.56
SPRR1A	small proline-rich protein 1A	3.20	1.58	2.48	2.22	2.37
SPRR1B	small proline-rich protein 1B (cornifin)	2.78	1.21	2.07	1.40	1.87
SPRR3	small proline-rich protein 3	3.72	1.13	2.22	2.21	2.32
SPTLC2	serine palmitoyltransferase, long chain base subunit 2	2.63	1.09	2.09	3.43	2.31
STAT1	signal transducer and activator of transcription 1, 91kD	2.99	13.28	2.81	3.16	5.56
STAT3	signal transducer and activator of transcription 3 (acute	1.63	1.00	1.13	1.40	1.29
STEAP4	STEAP family member 4	1.60	1.24	1.47	2.85	1.79
SYNCRIP	synaptotagmin binding, cytoplasmic RNA interacting pr	1.41	1.00	1.16	1.22	1.20
TCN1	transcobalamin I (vitamin B12 binding protein, R binder	7.20	2.44	6.03	7.45	5.78

SYMBOL	Description Up in all 4 studies	Yao_lgFCH	Zou_lgFCH	Gudjonsson_lgFCH	Suarez-Farinas_lgFCH	average
TGM1	transglutaminase 1 (K polypeptide epidermal type I, pr	3.07	1.52	2.19	2.45	2.31
TGM3	transglutaminase 3 (E polypeptide, protein-glutamine-g	2.49	1.50	1.95	1.87	1.95
TMEM165	transmembrane protein 165	1.29	1.14	1.50	1.16	1.27
TMPRSS11D	transmembrane protease, serine 11D	4.48	2.00	3.52	5.02	3.76
TNFSF10	tumor necrosis factor (ligand) superfamily, member 10	1.41	1.14	1.10	1.41	1.26
TOP2A	topoisomerase (DNA) II alpha 170kDa	1.30	1.14	1.45	1.15	1.26
TRIM22	tripartite motif-containing 22	1.89	1.49	1.24	1.49	1.53
TTC39A	tetratricopeptide repeat domain 39A	2.63	1.16	2.20	2.49	2.12
TYMP	thymidine phosphorylase	3.82	1.80	2.52	3.31	2.86
WNT5A	wingless-type MMTV integration site family, member 5/	2.52	1.99	2.38	3.44	2.58

SYMBOL	Description	Yao_lgFCH	Zhou_lgFCH	Gudjonsson_lgFCH	Suarez-Farinas_lgFCH	average
FA2H	fatty acid 2-hydroxylase	-1.40	-13.43	-1.17	-1.52	-5.33
WIF1	WNT inhibitory factor 1	-2.95	-2.45	-3.58	-3.37	-2.99
IL1F7	interleukin 1 family, member 7 (zeta)	-2.96	-1.64	-3.35	-3.06	-2.65
CCL27	chemokine (C-C motif) ligand 27	-2.85	-1.26	-3.10	-2.94	-2.40
CMAH	cytidine monophosphate-N-acetylneuraminic acid hydr	-2.48	-1.25	-1.36	-3.81	-1.69
ID4	inhibitor of DNA binding 4, dominant negative helix-loo	-2.37	-1.60	-2.09	-2.61	-2.02
GAL	galanin prepropeptide	-2.51	-1.77	-1.83	-2.42	-2.03
HSD11B1	hydroxysteroid (11-beta) dehydrogenase 1	-2.46	-1.00	-2.24	-2.80	-1.90
F3	coagulation factor III (thromboplastin, tissue factor)	-2.19	-1.99	-1.90	-2.34	-2.03
CHP2	calcineurin B homologous protein 2	-2.11	-1.46	-2.18	-2.54	-1.92
SCGB1D2	secretoglobin, family 1D, member 2	-2.09	-1.59	-1.31	-2.72	-1.66
ZDHHC11	zinc finger, DHHC-type containing 11	-2.62	-1.16	-1.62	-2.19	-1.80
FADS1	fatty acid desaturase 1	-1.56	-2.66	-1.17	-2.01	-1.80
PIP	prolactin-induced protein	-1.65	-1.75	-1.53	-2.37	-1.64
SORBS1	sorbin and SH3 domain containing 1	-2.11	-1.43	-1.47	-2.05	-1.67
RAI14	retinoic acid induced 14	-1.53	-1.93	-1.20	-2.27	-1.55
PTPN21	protein tyrosine phosphatase, non-receptor type 21	-1.53	-2.20	-1.57	-1.61	-1.76
AQP9	aquaporin 9	-1.29	-1.58	-1.99	-1.85	-1.62
CRIP1	cysteine-rich protein 1 (intestinal)	-2.10	-1.36	-1.13	-2.10	-1.53
PPARGC1A	peroxisome proliferator-activated receptor gamma, coa	-1.63	-1.43	-1.06	-2.22	-1.37
FAR2	fatty acyl CoA reductase 2	-2.04	-1.17	-1.50	-1.43	-1.57
NRN1	neuritin 1	-1.68	-1.18	-1.18	-2.07	-1.35
CA6	carbonic anhydrase VI	-1.45	-1.57	-1.16	-1.93	-1.39
FADS2	fatty acid desaturase 2	-1.70	-1.80	-1.08	-1.46	-1.53
MYLK	myosin light chain kinase	-1.42	-1.66	-1.24	-1.60	-1.44
GATA3	GATA binding protein 3	-1.35	-1.25	-1.57	-1.63	-1.39
CCND1	cyclin D1	-1.72	-1.39	-1.05	-1.62	-1.39
APOD	apolipoprotein D	-1.85	-1.23	-1.00	-1.63	-1.36
ITM2A	integral membrane protein 2A	-1.50	-1.03	-1.27	-1.84	-1.27
PCP4	Purkinje cell protein 4	-1.46	-1.01	-1.44	-1.74	-1.30
ADIPOQ	adiponectin, C1Q and collagen domain containing	-1.22	-1.04	-1.09	-2.25	-1.11
APOE	apolipoprotein E	-1.48	-1.50	-1.05	-1.55	-1.34
FOXC1	forkhead box C1	-1.85	-1.23	-1.02	-1.39	-1.37
CRAT	carnitine acetyltransferase	-1.19	-1.84	-1.08	-1.27	-1.37
CST6	cystatin E/M	-1.35	-1.41	-1.49	-1.08	-1.42
TGFBR3	transforming growth factor, beta receptor III	-1.54	-1.06	-1.02	-1.39	-1.21
ALDH3A2	aldehyde dehydrogenase 3 family, member A2	-1.20	-1.01	-1.07	-1.28	-1.09
MYH11	myosin, heavy chain 11, smooth muscle	-1.75	-1.06	-1.39	-1.81	-1.40
